# TANGO: direct optimization of constrained synthesizability for generative molecular design

**DOI:** 10.1038/s43588-026-00959-1

**Published:** 2026-03-03

**Authors:** Jeff Guo, Philippe Schwaller

**Affiliations:** 1 École Polytechnique Fédérale de Lausanne (EPFL), Lausanne, Switzerland; 2 National Centre of Competence in Research (NCCR) Catalysis, Zurich, Switzerland

**Keywords:** Machine learning, Cheminformatics

## Abstract

Constrained synthesizability is an unaddressed challenge in generative molecular design: in particular, designing molecules satisfying multi-parameter optimization objectives, while simultaneously being synthesizable and enforcing the presence of specific building blocks in the synthesis. This is practically important for molecule re-purposing, sustainability and efficiency. Here we propose the Tanimoto Group Overlap (TANGO) reward function, which uses chemistry principles to transform a binary reward function into a continuous reward function. TANGO can augment molecular generative models to directly optimize for constrained synthesizability using reinforcement learning (RL). Our framework is general and addresses starting-material, intermediate and divergent-synthesis constraints. Contrary to many existing works in the field, we show that incentivizing a general-purpose model with RL is a productive approach to navigating challenging synthesizability optimization scenarios.

## Abstract (web summary)

The authors propose the TANGO reward function, which enables the generation of property-optimized small molecules with predicted synthesis routes, incorporating a small set of shared precursors.

## Main

Synthesizable generative molecular design is becoming increasingly prevalent^1,2^, paralleling the rise in the number of experimentally validated generative-design case studies^3^. Controlling how generated molecules can be synthesized offers great potential for the push towards closed-loop discovery^4,5^, as molecules that can be made from specific reagents or reactions are naturally more amenable to robotic synthesis automation, which can be specialized for certain chemistries^6,7^. Moving beyond methods that optimize for synthesizability heuristics^2,8^, approaches that explicitly assess synthesizability can be broadly categorized into forward synthesis or retrosynthesis, which builds molecules from simple building blocks or recursively decomposes a target molecule into constituent building blocks, respectively. An example of forward synthesis in the context of molecular design is synthesizability-constrained molecular generation. These methods anchor molecular generation in viable chemical transformation rules, thus promoting synthesizability^9–15^. On the other hand, retrosynthesis planning^16–20^ proposes viable synthesis routes to a target molecule, and these models are often used as standalone tools to assess synthesizability. Such models have become increasingly adopted and are now routinely used to filter generated molecules^21^. Recent work has shown that generative models can directly generate molecules deemed synthesizable by retrosynthesis models by treating them as another oracle (computational prediction) to optimize for^22^. Subsequently, constrained synthesis planning has become a research focus, whereby proposed synthesis routes incorporate enforced building blocks (EBBs). This is especially relevant for sustainability and efficiency, and examples include semi-synthesis^23^ (starting from reagents isolated from natural sources) and divergent synthesis^24^ (passing through common intermediates).

More generally, existing works have introduced the notion of starting-material-constrained synthesis^25–27^, where a small set of fixed reagents is used in the first step of reaction pathways to probe reactivity. These reagents are specifically selected due to their reactivity^25^, as primordial substrates^26^ or as toxic wastes to be degraded ^27^. Drawing the connection to synthesis planning, existing works have explored reacting a small set of fixed reagents with a larger set of general reagents to achieve valorization. For example, Wołos et al.^28^ demonstrated that industrial waste reacted with ‘basic’ reagents can be valorized into known drug molecules. Similarly, the concept of ‘bioprivileged molecules’^29^ was introduced, where molecules specifically derived from biomass could be used to synthesize novel and useful chemicals. Therefore, exploring the ability to valorize a small set of specific reagents is a pertinent problem. More recently, constrained retrosynthesis algorithms have been proposed that guide the identification of synthesis pathways towards specific reagents^30–32^. However, to date, there are no molecular generative models that can enforce specific building blocks in the proposed routes.

**Fig. 1:**
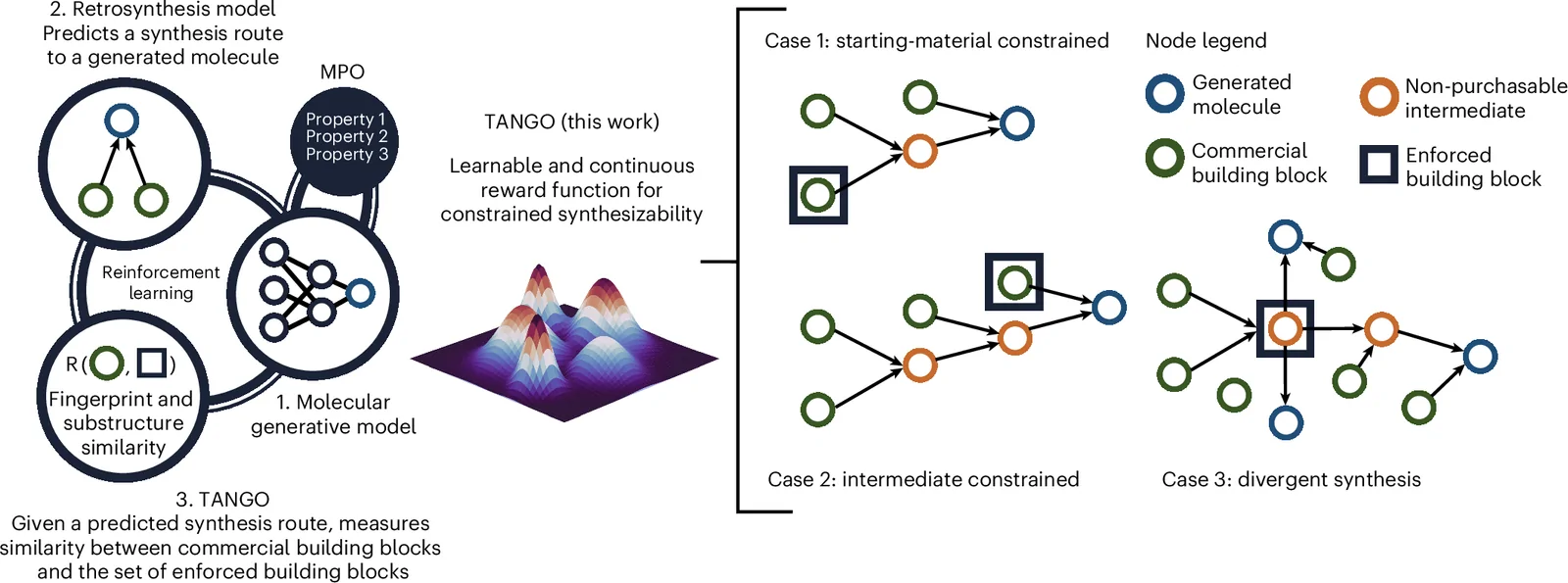
TANGO guides the generation of molecules directly optimized for constrained synthesizability with EBBs while simultaneously optimizing other properties. Our method generalizes across starting-material, intermediate and divergent-synthesis constraints.

In this work, we show that a general-purpose molecular generative model, without any hard constraints, can be incentivized to generate synthesizable molecules that satisfy multi-parameter optimization (MPO) objectives while jointly enforcing a set of building blocks (Fig. 1). To our knowledge, there are only a few works^30–33^ that enable some notion of building-block-constrained synthesis planning. In particular, the very recent DESP^32^ retrosynthesis search algorithm proposes a bidirectional search that can be constrained on a starting material. Our work differs in that we are proposing not a search algorithm, but rather a generative approach that jointly tackles constrained synthesizability and MPO. Our contribution is as follows. (1) We leverage chemistry principles and propose the Tanimoto Group Overlap (TANGO) reward function to generate molecules deemed synthesizable by retrosynthesis models with the presence of EBBs using reinforcement learning (RL). (2) We show that generated molecules satisfy MPO objectives, and by design enable the construction of synthesis networks where common intermediates branch towards diverse, high-reward molecules. (3) We show that letting a general-purpose model freely learn (using incentives) can be a productive approach to optimizing challenging synthesizability objectives.

## Results

### Making constrained synthesizability learnable

#### Understanding the optimization dynamics

In Supplementary Section 4, we perform ablation studies to understand the optimization dynamics that enable direct optimization of constrained synthesizability. We define fuzzy matching substructure (FMS) as the number of heavy atoms overlapping between a query molecule (molecule node in a synthesis graph) and an EBB. FMS_Block_ and FMS_Query_ denote whether the heavy-atom overlap is normalized by the EBB or the query molecule, that is, divided by the number of heavy atoms in the EBB or the query molecule, respectively. See ‘TANGO reward function’ for details of why we formulated two variants.

We summarize our observations around various reward functions and show synthesizability and molecular property and diversity metrics in Table 1 and Extended Data Table 1, respectively. (1) Standard Tanimoto similarity as the reward function can lead to successful runs, but it may be less stable as only 3/5 seeds were successful, with high variance (Solved (Enforced)). (2) Owing to false positives, FMS_Block_ is essentially unsuccessful, as on average it generates only one molecule satisfying constrained synthesizability (Solved (Enforced)). FMS_Query_ circumvents false positives and is more successful in generating Solved (Enforced) molecules. The docking scores (DSs) are much more optimized (lower values), but diversity can be lower (IntDiv1)^34^ (Extended Data Table 1). (3) TANGO combines Tanimoto similarity with FMS with equal weighting. TANGO-FMS_Block_ and TANGO-FMS_Query_ are the most consistent reward functions with 5/5 successful seeds. All seeds successfully generate Solved (Enforced) molecules, and much more than FMS_Query_ alone. Comparing the use of FMS_Block_ versus FMS_Query_ in TANGO, we find that FMS_Block_ is more performant, with better DSs, ligand efficiency and diversity (Extended Data Table 1). Considering these empirical results, the final implementation of TANGO defaults to computing FMS_Block_. Only in the event of a false-positive signal is FMS_Query_ computed instead. From here on, we refer to TANGO-FMS_Block_ as TANGO. (4) Through ablation studies, we show that ‘it takes two to tango’ because TANGO’s performance is much more consistent and robust (across seeds) than using just Tanimoto similarity or FMS (Block or Query) alone.

**Table 1:** Reward function experiments

Configuration *N*: no. of seeds/5 generating ≥1 Solved (Enforced)	Synthesizability			Solved (Enforced) molecules Reaction values: pooled (*M* pooled molecules) Unique enforced block values: per seed		Oracle budget (wall time)
	Non-solved	Solved	Solved (Enforced)	No. of reaction steps	No. of unique enforced blocks	
TanSim (*N* = 3)	2,275 ± 143	7,653 ± 142	1,864 ± 1,828	2.21 ± 1.16 (*M* = 8,731)	1.33 ± 0.47	10,000 (9 h 11 min ± 48 min)
FMS_Block_ (*N* = 4)	2,166 ± 293	7,792 ± 304	1 ± 0	2.14 ± 1.46 (*M* = 7)	1.50 ± 0.50	10,000 (7 h 35 min ± 22 min)
FMS_Query_ (*N* = 3)	1,967 ± 226	8,002 ± 241	315 ± 563	2.67 ± 1.13 (*M* = 1,546)	1.33 ± 0.47	10,000 (8 h 6 min ± 23 min)
TANGO-FMS_Block_ (*N* = 5)	2,229 ± 325	7,711 ± 333	1,744 ± 715	2.38 ± 1.25 (*M* = 8,315)	2.20 ± 0.75	10,000 (8 h 12 min ± 15 min)
TANGO-FMS_Query_ (*N* = 5)	2,000 ± 211	7,945 ± 217	1,864 ± 1,066	2.24 ± 1.18 (*M* = 8,472)	1.20 ± 0.40	10,000 (8 h 14 min ± 11 min)

Synthesizability metrics across different reward functions: Tanimoto similarity (TanSim), FMS normalized by the EBB (FMS_Block_) or query molecule (FMS_Query_), and TANGO-FMS_Block_ or TANGO-FMS_Query_, both with 0.50 FMS and 0.50 Tanimoto similarity weighting. All experiments were with 100 EBBs. The number of seeds (out of five) with at least one generated molecule satisfying constrained synthesizability is reported as *N*. Metrics (for constrained synthesizable molecules) are reported in two settings: (1) per seed, averaged across seeds; (2) pooled, pooling all molecules across seeds and de-duplicating. The number of pooled, de-duplicated molecules is denoted by *M*.

Source data

Thus far, TANGO was formulated with equal weighting to FMS_Block_ and Tanimoto similarity. We next investigated varying the weighting, assigning 0.75 to either FMS_Block_ or Tanimoto similarity and 0.25 to the other. The results in Supplementary Section 4.5 show that putting more weight on Tanimoto similarity leads to more constrained synthesizable molecules, but at the expense of worse MPO. Since MPO is vital for practicality, we chose to designate TANGO with equal weighting as the default reward function. Next, we found that the default hyperparameters of Saturn^35^ are too exploitative and disadvantageous in this optimization setting. Relaxing this behavior makes constrained synthesizability much more consistently learnable (under the fixed oracle budget). Finally, once molecules with a specific EBB are generated, Saturn heavily focuses on that building block, such that within the same generative experiment often only one unique block is enforced (but variable across seeds). We do not consider this a disadvantage, as it enables the construction of divergent synthesis networks, where a common block branches towards many optimal molecules. We discuss the implications of this behavior from a generative perspective in the next section.

#### Constrained synthesizability results

Table 2 shows the synthesizability metrics for the *B*_enf-10_ and *B*_enf-100_ experiments using TANGO. Extended Data Table 2 shows the molecular property and diversity metrics. We make the following observations. (1) Constrained synthesizability can be learned within the 10,000-oracle budget (approximate 8.5 h wall time). (2) All runs generate non-solvable molecules and many solvable molecules do not contain the enforced blocks, as expected (see Non-solved, Solved and Solved (Enforced) metrics). Nonetheless, generated molecules can achieve favorable DSs (tending towards <−10, which is considered optimal in previous works^12^) and optimal quantitative estimate of druglikeness (QED)^36^ values. This demonstrates the capability to perform MPO while also optimizing for constrained synthesizability. (3) The negative control is the ‘Unconstrained’ experiment, which does not explicitly optimize for the use of EBBs. We expect generated molecules not to satisfy constrained synthesizability, and this is affirmed by the results in Table 2, showing that only four molecules in total (across ten seeds), by chance, satisfy this constraint. In this experiment only, we report the diversity metrics for the Solved molecules and observe that the IntDiv1, #Circles and number of Bemis–Murcko scaffolds found is much higher than in constrained experiments (Extended Data Table 2). This is expected because constrained synthesizability, by definition, essentially fixes a portion of the molecule by using EBBs. (4) Number of unique enforced blocks is relatively low, as we observed that once the model incorporates one EBB it focuses on generating molecules whose syntheses can be decomposed to that specific block, since the reward it obtains is high and there is a degree of exploitation. This also contributes to the decreased diversity (see IntDiv1, #Circles and #Scaffolds metrics) compared with the Unconstrained setting, and is an expected outcome (Extended Data Table 2). (5) The starting-material constraint is more difficult for *B*_enf-100_, but unexpectedly not necessarily for B_enf-10_ (there are 9/10 successful seeds compared with 6/10 for the non-starting-material case). We speculate that the reason for this is exactly due to exploitation behavior. Since TANGO returns the maximum reward in the synthesis tree (comparing with all *B*_enf_), it is possible that more blocks can be a hindrance when there are specific blocks that are particularly favorable. We emphasize that across different seeds the EBBs can be different, which is important, as we could run multiple experiments in parallel and pool the results. (6) Number of reaction steps is generally small, which shows that optimizing for constrained synthesizability does not lead to inefficient synthesis plans. We note that the number of reaction steps in the divergent synthesis results is greater because it takes one step in the first place to arrive at the divergent blocks. Finally, all runs took 8.5 h on average using a single graphics processing unit (GPU), which is reasonable.

**Table 2:** Constrained synthesizability experiments

Configuration *N*: no. of seeds/10 generating ≥1 Solved (Enforced)	Synthesizability			Solved (Enforced) molecules Reaction values: pooled (*M* pooled molecules) Unique enforced block values: per seed		Oracle budget (wall time)
	Non-solved	Solved	Solved (Enforced)	No. of reaction steps	No. of unique enforced blocks	
Unconstrained (*N* = 10)	1,827 ± 191	8,128 ± 197	4 in total^**a**^	1.88 ± 1.21 (*M* = 74,155)	1 ± 0	10,000 (5 h 34 min ± 39 min)
100 blocks (*N* = 10)	2,288 ± 305	7,649 ± 309	2,112 ± 1,170	2.42 ± 1.27 (*M* = 19,221)	1.9 ± 0.70	10,000 (8 h 31 min ± 40 min)
100 blocks (SM) (*N* = 10)	1,879 ± 186	8,067 ± 180	1,525 ± 503	1.59 ± 1.00 (*M* = 11,299)	1.70 ± 0.78	10,000 (8 h 33 min ± 30 min)
10 blocks (*N* = 6)	2,424 ± 288	7,498 ± 292	984 ± 1,182	2.71 ± 1.22 (*M* = 8,562)	1.00 ± 0	10,000 (8 h 29 min ± 30 min)
10 blocks (SM) (*N* = 9)	2,225 ± 181	7,703 ± 192	1,004 ± 926	2.59 ± 1.07 (*M* = 8,311)	1.00 ± 0	10,000 (8 h 39 min ± 24 min)
Divergent blocks (*N* = 4)	2,166 ± 203	7,770 ± 214	651 ± 1,238	3.69 ± 1.08 (*M* = 6,372)	1.75 ± 0.83	10,000 (8 h 52 min ± 42 min)

Synthesizability metrics with the TANGO reward function (0.50 FMS_Block_ and 0.50 Tanimoto similarity weighting). ‘SM’ denotes starting-material constrained. ‘Unconstrained’ denotes the experiment without enforcing building blocks, as a comparison. The number of seeds (out of ten) with at least one generated molecule satisfying constrained synthesizability is reported as *N*. Metrics (for constrained synthesizable molecules) are reported in two settings: (1) per seed, averaged across seeds; (2) pooled, pooling all molecules across seeds and de-duplicating. The number of pooled, de-duplicated molecules is denoted by *M*. ^a^There is no notion of ‘enforced blocks’ in the Unconstrained setting, so the metrics are reported for Solved molecules.

Source data

#### Are the results by chance?

**Fig. 2:**
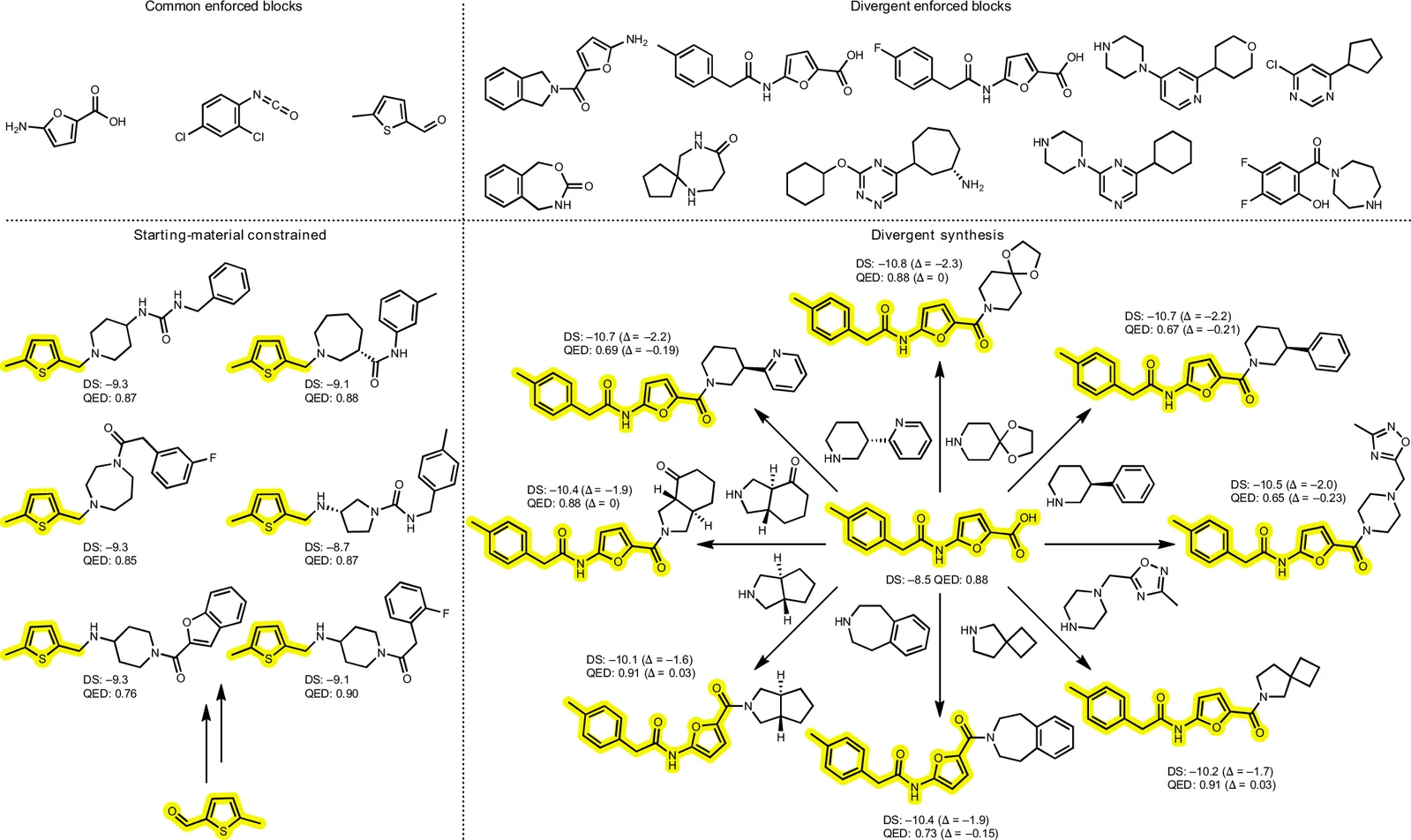
Example molecules generated under the starting-material and divergent-synthesis (one-step synthesis from a non-commercial common intermediate to diverse, high-reward molecules) constraints. The specific EBB is highlighted in yellow. The DSs and the QED values are annotated. For the divergent-synthesis example, ΔDS (negative is better) and ΔQED (positive is better) are additionally annotated. Source data

While the results thus far were promising, we noticed that many runs (across seeds) converged to the same three EBBs (Fig. 2). We questioned whether the success was simply due to these ‘lucky’ blocks. Therefore, we performed a set of ablation experiments by removing these blocks and re-running the configurations in Table 2. The results show that the model can generate optimal molecules with other EBBs (Supplementary Section 4.7). These runs were much less successful (across seeds) but recovered when removing the QED objective. This suggests that the model learns to use certain blocks that are more aligned with the objective function. Next, we further push our framework with an EBB set of five molecules dissimilar to the lucky blocks. While much more challenging (most runs are unsuccessful under the strict oracle budget), this is still possible, with the routes containing Suzuki coupling reactions (Supplementary Section 4.8), which is notably different from the amide coupling reactions in Fig. 2.

#### Synthesis networks

Next, we tackle divergent synthesis by incorporating larger, non-commercial molecules in the EBB set. We curated a set of ten non-commercial blocks from solved synthesis routes (Fig. 2) and ran the same experimental set-up. Table 2 shows that this is also learnable within the oracle budget, albeit much less consistently, as only 4/10 seeds were successful. We still believe that our framework is robust, as this is a much more challenging task and we wanted to show that the model can learn to enforce these large building blocks from scratch. We can also increase the oracle budget, which leads to more successful seeds, as we show in Supplementary Section 4.9. This capability opens up practical applications for late-stage functionalization, commonly employed in drug discovery^37^. Correspondingly, Fig. 2 shows example synthesis networks using results from the starting-material- and divergent-synthesis-constrained experiments. All generated molecules achieve optimal DSs (although starting-material constrained resulted in slightly worse scores) and QED values. In the divergent-synthesis case, a one-step amide coupling reaction from the enforced block leads to notably improved DSs, though sometimes with lower QED. Examples of full synthesis routes are shown in Supplementary Section 5.1.

### Learning a desirable distribution

**Fig. 3:**
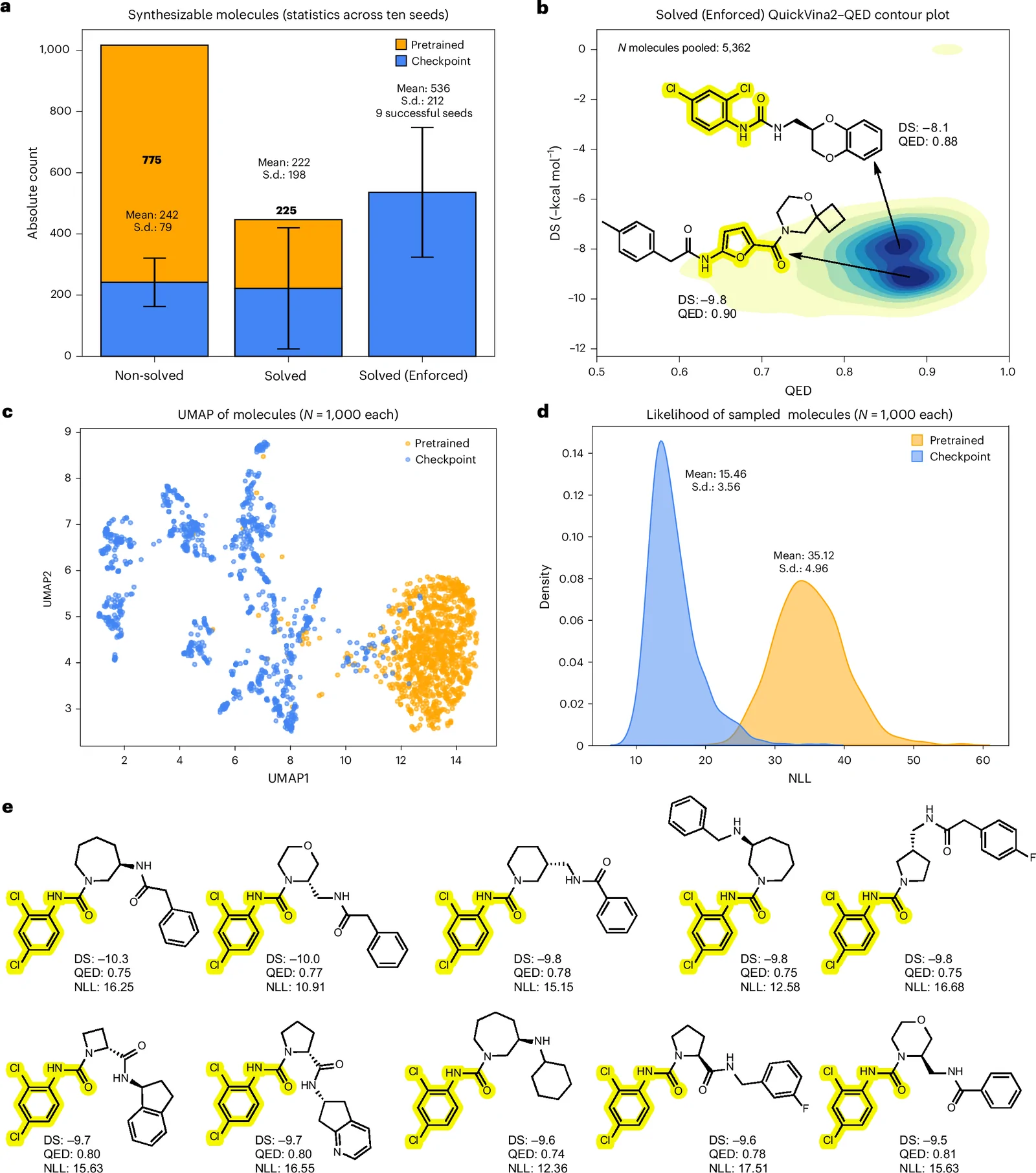
The model learns a distribution of molecules that satisfies the MPO objective. **a**–**e**, The final model checkpoint from the 100-EBB experiment (all ten seeds) was used to sample 1,000 unique molecules. The specific EBB is highlighted in yellow. **a**, Counts of solvable molecules from the checkpoints with the mean and s.d. reported (non-bold). 1/10 final model checkpoints was unable to yield Solved (Enforced) molecules. The pretrained model (before RL) generates mostly unsynthesizable molecules and no synthesizable molecules with enforced blocks (metrics are bold). **b**, Contour plot showing the density of DS and the QED values of the pooled Solved (Enforced) molecules across all seeds. Panels **c**–**e** use 1,000 unique molecules sampled from one final model checkpoint. **c**, The UMAP of sampled molecules compared with those from the pretrained model. **d**, NLLs of the sampled molecules. It is much more likely to generate the sampled molecules under the final model checkpoint (lower NLL). **e**, Top-ten (by DS) molecules with the EBB highlighted. Source data

Fundamentally, generative models learn to model distributions. In this section, we further demonstrate that TANGO is a learnable reward function and that the modeled distribution shifts to satisfy the MPO objective. To do so, we take each final model checkpoint (across the ten seeds) from the experiment in Table 2 with 100 EBBs (and with QED) and sample 1,000 unique molecules. Figure 3a shows that a considerable number of sampled molecules are jointly synthesizable with an EBB (Solved (Enforced)). The distribution shift is apparent when compared with 1,000 unique molecules sampled from the pretrained model (before RL), which mostly generates unsynthesizable molecules (Non-solved). Figure 3b pools (across the ten seeds) all the Solved (Enforced) molecules and shows the density of docking and QED scores that have shifted towards favorable values. Next, we take the sampled molecules from one seed and plot a uniform manifold approximation and projection (UMAP)^38^ embedding, comparing with the molecules sampled from the pretrained model. It is clear that the checkpoint-sampled molecules are dissimilar but we show that the learned distribution is not perfect, as the final checkpoint still sometimes samples ill-suited (on the basis of the MPO objective) molecules that are similar to the pretrained model. Subsequently, we take the sampled molecules from the final model checkpoint and compare the negative log-likelihoods (NLLs) as measured by this checkpoint and the pretrained model. A lower NLL means the molecule is more likely to be generated and vice versa. We make two observations: first, the molecules are much more likely under the checkpoint, unsurprisingly. However, second, and more importantly, the NLLs from the checkpoint put more probability mass in a narrower region. We now cross-reference Fig. 3e, which shows the top-ten sampled molecules (by DS), which all share the same EBB. The NLLs are not markedly different, which shows that some exploitation during RL can be advantageous as the likelihoods of molecules that share a common structure can be quite similar. The model learns to use the building blocks in a way that performs local exploration and assigns a relatively similar likelihood to the neighborhood of molecules. Overall, the results show that taking a general-purpose model and incentivizing the learning process with TANGO can shift the modeled distribution to one that captures constrained synthesizability, while simultaneously satisfying MPO objectives. This is practically useful, as one could simply sample molecules from model checkpoints to obtain more desirable molecules (it takes about 5 s to sample 1,000 unique molecules).

### Enforcing phenylsulfonamide building blocks (COX-2 case study)

In this section, we apply our framework to a real-world-inspired case study to design selective inhibitors for cyclooxygenase (COX) to treat pain and inflammation^39^. From existing structure–activity relationship studies, the cis-stilbene and phenylsulfonamide moieties can confer selectivity for COX-2 over COX-1, where inhibiting the latter has been associated with toxicity^39^. We note that existing selective COX-2 inhibitors containing these specific moieties (rofecoxib and valdecoxib) have also led to cardiovascular complications and were withdrawn from the therapeutics market^39,40^. This suggests that the presence of cis-stilbene and phenylsulfonamide, while important for selectivity, does not fully account for the efficacy and safety of the potential drug molecule.

#### Experimental details

This case study mimics a plausible design campaign to pivot from a known selective inhibitor by exploring other scaffolds, while fixing a specific moiety. SC-558 is a selective COX-2 inhibitor possessing the cis-stilbene and phenylsulfonamide moieties^41^. We focus on phenylsulfonamide and extract all the building blocks containing this moiety from the same ZINC-derived commercial stock^42^ used in this work. Out of 17,721,980 building blocks in the ZINC stock, 139 contain phenylsulfonamide, and we use TANGO to guide molecular generation to use these specific blocks. We run this experiment across five seeds (0–4 inclusive) with a 15,000-oracle budget and jointly minimize the Gnina^43,44^ DS and maximize QED^36^.

#### Results

**Fig. 4:**
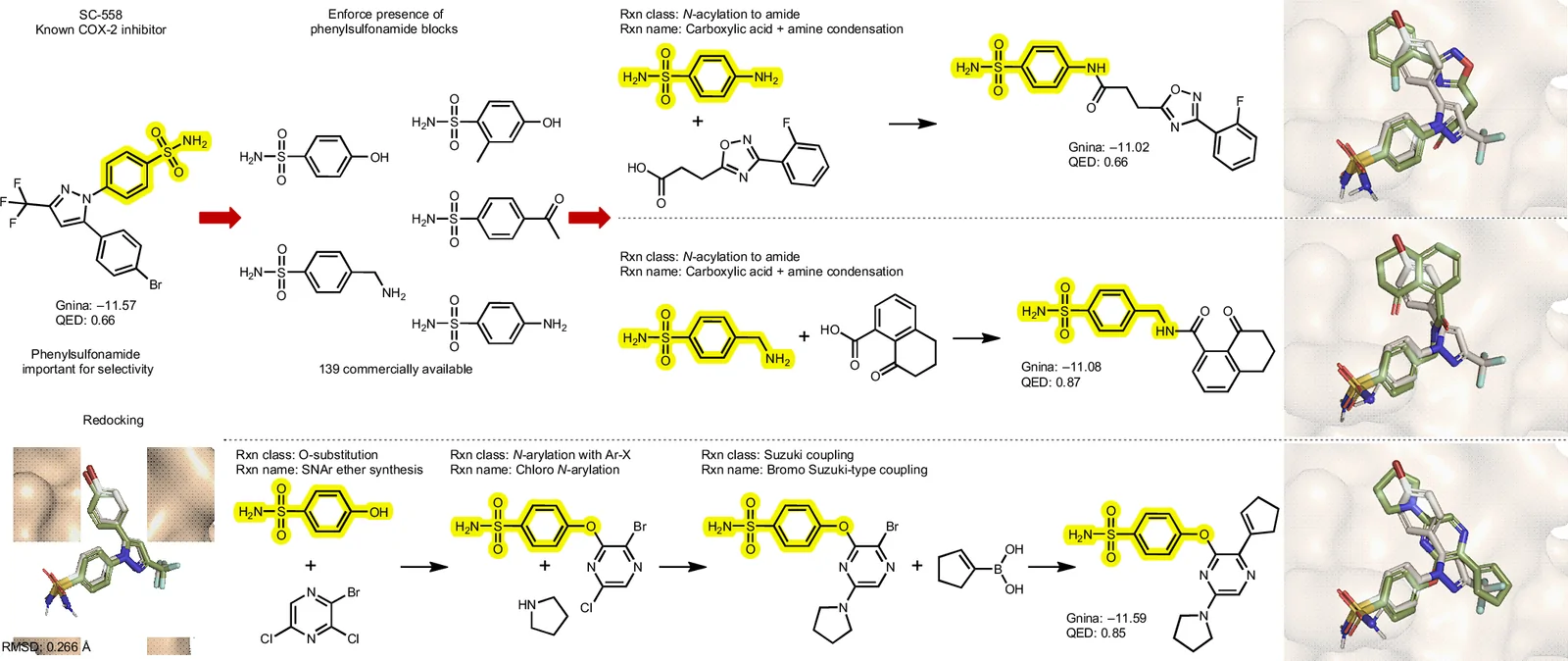
COX-2 case study. Phenylsulfonamide is important for selectivity in known COX-2 inhibitors, such as SC-558. Redocking using Gnina reasonably reproduces the co-crystallized binding pose. The case study is to generate docking-optimized molecules that have synthesis routes specifically using phenylsulfonamide building blocks to retain this moiety. The specific EBB is highlighted in yellow. Three example generated molecules are shown with the corresponding synthesis route, where the reaction (Rxn) classes and names are labeled with NameRxn software. The Gnina DSs and the QED are annotated. All docked poses are shown in green and compared with the crystal pose of the reference SC-558 ligand in gray. RMSD, root mean square deviation. Source data

To promote the plausibility of generated molecules, we redock SC-558 using Gnina to first verify that the docking protocol can reproduce the crystal binding pose. Figure 4 shows that the redocked pose (green) has a 0.266-Å root mean square deviation from the crystal pose (gray), and places the phenylsulfonamide in the correct area, albeit with a slightly rotated orientation. Extended Data Tables 3 and 4 show that TANGO successfully guides the generation of phenylsulfonamide-containing molecules. We make the following observations. (1) The number of unique enforced blocks is much larger than in the experiments in the previous sections and indicates that many of the 139 phenylsulfonamide-containing blocks were used. This is somewhat expected as the EBBs here have high similarity (Fig. 4), in contrast to the set of EBBs in the previous section, which we randomly sampled from the commercial stock. (2) Diverse molecules are still generated (IntDiv1 and #Circles metrics). (3) The wall time is much longer than in previous experiments because Gnina is more computationally intensive compared with QuickVina2-GPU. (4) The reference ligand, SC-558, is a known selective COX-2 inhibitor with a Gnina DS of −11.57. Generated molecules can achieve similar or better DSs with the docking algorithm placing the phenylsulfonamide in a similar orientation (Fig. 4). We acknowledge that DSs are a crude estimate of binding affinity, and the purpose of this section is to demonstrate the applicability of TANGO. Accordingly, some generated molecules are predicted to be synthesizable with a one-step amide coupling (reactions were labeled with NameRxn^45^) and the binding pose is plausible. Overall, the results demonstrate that TANGO can be used to fix specific chemical moieties of interest and explore other scaffolds that also satisfy the optimization objective.

## Discussion

In this work, we have proposed a reward function called TANGO that can guide a general-purpose molecular generative model to directly optimize for constrained synthesizability. The framework demonstrates the feasibility of a generative approach for constrained synthesizability, and tackles various degrees of constraints that are practically important in real-world applications: starting-material, intermediate and divergent-synthesis constraints (Fig. 2).

The results show that TANGO can generalize across different EBB sets, but there are several important limitations. First, TANGO’s success relies on formulating constrained synthesizability as a continuous reward function, which enables providing a signal on whether we are getting ‘closer’ to achieving the objective. In Supplementary Section 4.3, we support this statement by assessing the brute-force approach, meaning assigning 1 or 0 as the reward, indicating satisfying constrained synthesizability or not, respectively. The results show that this brute-force approach is much less reliable, as more seeds are unsuccessful and the MPO is worse. However, while TANGO itself is a continuous reward function, it requires that a synthesis route is solved, in the first place, by the retrosynthesis model. If a synthesis route is not found for a molecule, TANGO cannot be computed and the reward is defaulted to 0. This imposes a binary aspect in TANGO. In all the experiments performed in this work, we found that this was not detrimental to the optimization process, but we highlight that we used 17,721,980 building blocks (stock) in the retrosynthesis model. Naturally, a larger stock means that more molecules can be solved by the retrosynthesis model. Accordingly, under a more constrained stock, it is possible that the binary aspect of finding a synthesis route becomes an optimization challenge. Another limitation is that ‘true synthesizability’ depends on the accuracy of the retrosynthesis model, and these are not perfect. It is likely that many synthesis routes themselves are not synthetically feasible and/or lack regio- or stereo-selectivity^46^. This is a limitation of current retrosynthesis models and is an ongoing challenge for improved synthesis planning. As we are not proposing a new retrosynthesis model, this is beyond the scope of this work, but we believe this is important to explicitly acknowledge.

Our framework also provides opportunities to further understand the optimization dynamics and to extend TANGO to more use cases. In this work, we investigated various reward function formulations (Supplementary Section 4.3) and their ability to guide learning towards achieving constrained synthesizability. Another factor that may notably affect optimization is the number of synthesis routes to return from the retrosynthesis model. Typically, retrosynthesis models are tasked to return a set of potential synthesis routes for further prioritization^47–50^. Here, we terminate route searching if one successful synthesis route has been found, for computational efficiency purposes. In general, there are numerous potential ways to synthesize a target molecule. Since TANGO measures the similarity between molecule nodes in the predicted synthesis route and the EBBs, returning more synthesis routes might make the optimization more tractable, given that some routes may have molecule nodes more similar to the EBBs. We believe that this is an interesting research avenue, as running route searching for longer will also increase the computational cost. Thus, understanding the interplay between optimization efficiency and computational cost would be pertinent. Going further, this work only focuses on enforcing a single building block. In Supplementary Section 5.3, we find that there are cases, albeit rare, where more than one EBB is present. Correspondingly, incentivizing the use of more than one EBB would necessitate further understanding of the optimization dynamics and can have practical applications. For example, generating molecules with synthesis routes incorporating multiple industrial by-products^28^ or biomass-derived molecules^29^ could be hugely advantageous in molecular re-purposing efforts.

## Methods

In this section, we describe the problem formulation of constrained synthesizability, the generative model, the TANGO reward function and the experimental set-up.

### Constrained synthesizability problem formulation

In synthesis planning, the goal is to propose a valid synthetic route to a target molecule using (commercially) available building blocks, *B*, and a set of reaction rules, *R*. We define a synthesis graph, *G*(*M*, *R*), where each node represents an intermediate molecule, *m*, that need not necessarily be an available building block, *b*, and the edges represent reactions, *r* ∈ *R*. The depth of a node is the number of edges from the root node (the target molecule). A valid synthetic route requires that all leaf nodes correspond to commercially available building blocks, *b* ∈ *B*. We further define the set of EBBs, *B*_enf_ ⊆ *B*. In practice, ∣*B*_enf_∣ ≪ ∣*B*∣, and in this work we consider ∣*B*_enf_∣ ∈ {10, 100}. We address three cases of constrained synthesis in this work.

### Starting-material-constrained synthesis (case 1)

A synthesis graph is considered starting-material constrained if at least one leaf node, *m* ∈ *G*(*M*, *R*), satisfies both of the following conditions: (1) *m* = EBB ∈ *B*_enf_ and (2) depth(*m*) = max depth,

$$\exists \,m\,\in \,G(M,\,R)\,{\rm{such}\,}{\rm{that}}\,{\mathrm{depth}}(m)\,=\,\max \,{\mathrm{depth}}\,{\mathrm{and}}\,m\,=\,{\mathrm{EBB}}\,\in \,{{{B}}}_{\mathrm{enf}}.$$

A practical reason for enforcing a starting-material constraint is that they may be inexpensive reagents. As such, they can be obtained in larger quantities for use in multi-step synthesis, which necessarily loses material at every synthetic step.

### Intermediate-constrained synthesis (case 2)

A synthesis graph is considered intermediate constrained (general case of starting-material constrained) if at least one intermediate node, *m* ∈ *G*(*M*, *R*), belongs to *B*_enf_, and depth(*m*) ≠ max depth:

$$\exists \,m\in G(M,\,R)\,{\rm{such}}\,{\rm{that}}\,{\mathrm{depth}}(m)\ne \max \,{\mathrm{depth}}\,{\mathrm{and}}\,m={\mathrm{EBB}}\in {B}_{\mathrm{enf}}.$$

### Divergent synthesis (case 3)

A synthesis graph is considered divergent if at least one intermediate node, *m* ∈ *G*(*M*, *R*), satisfies both of the following conditions: (1) *m* = EBB ∈ *B*_enf_ and (2) all EBB ∈ *B*_enf_ are non-commercial. The nuance of non-commercial is that they can be highly specific building blocks and potentially much larger in size than common commercial building blocks, which can enable late-stage functionalization^37^:

$$\begin{array}{l}\exists \,m\,\in \,G(M,\,R)\,{\rm{such}}\,{\rm{that}}\,\forall \,\mathrm{EBB}\,\in \,{B}_{\mathrm{enf}},\,\mathrm{EBB}\,\mathrm{is}\,\mathrm{non}-\mathrm{commercial},\,\mathrm{and}\,\\m\, =\,\mathrm{EBB}\,\in \,{B}_{\mathrm{enf}}.\end{array}\,\,$$

### TANGO reward function

In the context of generative molecular design, previous work has shown that retrosynthesis models can be treated as an oracle and directly optimized for^22^. The effect is that generated molecules are synthesizable, as deemed by retrosynthesis models (from here on, this will just be referred to as ‘synthesizable’, for brevity). In that work, we adopted a brute-force approach to learning synthesizability, despite the reward signal being binary, meaning *R* ∈ {0, 1}, denoting whether a molecule is synthesizable or not. This worked because there are enough molecules that are synthesizable, making the optimization landscape not too sparse. However, in the constrained synthesis setting, it is highly unlikely that a synthesizable molecule will also contain an EBB in its synthesis graph, especially when the number of *B*_enf_ is small, which is common in real-world applications^28^. Consequently, molecules satisfying constrained synthesizability are sparse: without a way to inform the model if it is getting closer to achieving the goal, learning becomes extremely challenging. Intuitively, defining a reward signal that is not exactly the target objective but is informative to achieving the target objective should guide learning, and can be done through reward shaping^51^.

In this work, we propose the TANGO reward function that leverages chemistry inductive bias to transform the binary reward environment associated with constrained synthesis to a continuous reward environment (Supplementary Fig. 1). Specifically, for every synthesizable molecule, TANGO provides a signal on whether the model is closer to incorporating *B*_enf_. Given *G*(*M*, *R*), this is achieved by a notion of similarity between every node, *m*, and *B*_enf_. We draw inspiration from previous works that leverage Tanimoto similarity (subgraph similarity) for retrosynthesis^18,52^. However, Tanimoto similarity alone is insufficient, as chemical reactivity is often associated with functional groups and their neighborhoods, which dictate incompatibilities^46^. Correspondingly, we experiment with augmenting Tanimoto similarity with functional group overlap (does a given molecule have similar functional groups to *B*_enf_?) and FMS (what is the maximum substructure overlap by atom count with *B*_enf_?). In the most straightforward formulation, substructure matching is a binary verifier: 1 if the substructure is contained and 0 otherwise. FMS, by contrast, formulates verification as a continuous signal: even if the exact substructure is not contained, what fraction of the substructure is? Concretely, the first step is to compute the number of overlapping heavy atoms in a query molecule node of a synthesis graph (an example is shown in Supplementary Fig. 1) with an EBB. Next, we define FMS_Block_ and FMS_Query_, denoting whether the number of overlapping heavy atoms is divided by the total number of heavy atoms in the EBB or the query, respectively. In the case of FMS_Block_, if the number of overlapping heavy atoms is equal to the total number of heavy atoms in the EBB, then the molecule node is an exact match, indicating that it has the same chemical structure as the EBB. In initial experiments, we found an edge case that led to false positives being matched. This occurs when the molecule node itself contains a substructure that is an exact match with the EBB. In this case, the overlapping heavy atoms are equal to the number of heavy atoms in the EBB, yet the molecule node is not structurally equivalent. FMS_Query_ handles this edge case by normalizing by the query molecule. When using FMS as a standalone reward function, we used the FMS_Query_ formulation to avoid false positives. Finally, it is entirely possible that there exists a scenario where FMS_Query_ is the one that leads to false positives over FMS_Block_. We did not observe this given our building-block sets, but to guard against such a scenario the implementation of FMS we use is to compute FMS_Block_ first. Next, if FMS_Block_ is 1, then we verify that the query and EBB are structurally equivalent. If not, this is a false-positive case, and FMS_Query_ is returned instead. See Supplementary Section 4.1 for a more detailed discussion of this edge case.

Subsequently, FMS (regardless of whether Block or Query) also naturally encodes a notion of conserved substructure, as rarely are molecular structures fragmented during a synthesis. If a molecule is used as a building block, a large portion of the intact structure should still be present in the final molecule. This has important implications, as the likelihood of generating a molecule that conserves a large substructure might mean that a certain level of exploitation in the sampling behavior can be beneficial^35^. We comment on this in ‘Results’. We further note that FMS (Block or Query), if enforcing exact atom hybridization, atom type, chirality and whether the atom is part of a ring, also implicitly considers functional group overlap. In Supplementary Section 4.3, we systematically evaluated various TANGO formulations and their ability to distinguish ‘goodness’, and conclude that equal weighting (0.5) of Tanimoto similarity (TanSim) and FMS_Block_ yields the best learnable signal:

1 $$\begin{array}{l}\mathrm{TANGO}(m,\mathrm{EBB})=\mathrm{TanSim}(m,\mathrm{EBB})\times 0.5+{\mathrm{FMS}}_{\mathrm{Block}}(m,\mathrm{EBB}) \times 0.5\in [0,1].\end{array}\,\,$$

To compute Tanimoto similarity, this work uses Morgan fingerprints with radius = 2 and nBits = 1,024. We note that, since the maximum value of Tanimoto similarity and FMS is 1, TANGO is by design already normalized ∈[0, 1]. Note also that using FMS_Block_ instead of FMS_Query_ does not lead to false positives in this case. The reason is that the Tanimoto similarity would never equal 1 if the molecule node and EBB are not exactly equivalent. Nonetheless, we compute FMS_Block_ by default, and fall back to FMS_Query_ if the FMS is 1 but the EBB and query are not structurally equivalent. The TANGO reward is the maximum value between all non-root nodes, *m* ∈ *G*(*M*, *R*) (Supplementary Algorithm 1). It follows that the type of constrained synthesizability can be controlled by a simple toggle of whether only nodes at max depth (starting material) or any node (intermediate or divergent) are considered.

### Molecular generative model

Here, we build on Saturn^35^, which is a general-purpose autoregressive language-based model operating on SMILES strings^53^. Saturn uses the Mamba^54^ architecture and performs goal-directed generation using RL. The key mechanism is combining SMILES augmentation^55^ with experience replay^56^ (Augmented Memory^57^), which directly controls the exploration–exploitation trade-off. In the original work, we found that aggressive local sampling in chemical space improves sample efficiency across various drug-discovery case studies. By contrast, we show that the constrained synthesizability setting necessitates a more exploratory behavior. We pretrain Saturn on PubChem^58^ after data preprocessing (Supplementary Section 1).

### Retrosynthesis model

In this work, we integrate Syntheseus^49^, which is a wrapper around various retrosynthesis models and search algorithms, into Saturn. Through Syntheseus, we use MEGAN (graph-edit based)^59^ as the single-step retrosynthesis model coupled with the Retro*^60^ search algorithm with default hyperparameters. MEGAN was chosen due to its fast inference speed, but we emphasize that our framework is retrosynthesis model agnostic. All generated molecules are sent for retrosynthetic analysis by MEGAN and a predicted synthesis route is returned, if any.

### Commercial building blocks

In this work, *B* is comprised of the ‘Fragment’ and ‘Reactive’ subsets of ZINC^42^ (17,721,980), which are part of the commercial building-block stock used in the AiZynthFinder^47,48^ retrosynthesis model. We note that the size of *B* is much larger than employed in previous synthesizability-constrained works^11,12,14,15^. Therefore, an additional result in this paper is showing that our framework can navigate an enormous synthesizable space. We also want to highlight that further increasing the size of *B* is straightforward, and does not require re-training of the generative model. Next, *B*_enf_ ⊂ *B* is randomly sampled with the following criteria: 150 < molecular weight < 200, no aliphatic carbon chains longer than 3, exclude charged molecules, if rings are present enforce size ∈ {5, 6}, and molecules must contain at least one nitrogen, oxygen or sulfur atom. We believe these criteria are a reasonable representation of simple building blocks applicable to drug design (see Supplementary Section 2.2 for more details) We consider ∣*B*_enf_∣ ∈ {10, 100} and denote these as *B*_enf-10_ and *B*_enf-100_, respectively.

### Drug discovery case study

The MPO optimization task for method development is to generate molecules with optimized QuickVina2-GPU-2.1^44,61,62^ DSs against ATP-dependent Clp protease proteolytic subunit (ClpP)^63^ (implicated in cancer) and high QED^36^, and that are synthesizable with either the starting-material, intermediate or divergent-synthesis constraints. We finally note that in rare cases Saturn can generate radicals due to the SMILES syntax: for example, when a ‘[C@H]’ token is generated in place of ‘c’ when constructing a ring. In principle, undesired radicals would be penalized by the oracle, but we noticed that the docking oracle in this work may be overly lenient. As this is a fundamental limitation of the oracle itself, we believe it does not hinder method development in this work.

### Experimental details

For method development, we ran every experiment across five seeds (0–4 inclusive) with varying oracle budgets. Once we identified optimal hyperparameters, we ran all main development experiments across ten seeds (0–9 inclusive) with a 10,000-oracle budget, and reported the wall time to promote practical application. We focus our investigation on the optimization dynamics for learning constrained synthesizability and the implications of TANGO. Finally, we apply our framework on a real-world-inspired case study to design selective COX-2 inhibitors by fixing a specific chemical moiety known to be important for selectivity^39^. We defer experiment-specific details to the corresponding Results section.

### Metrics

We report Non-solved, Solved and Solved (Enforced) as the number of generated molecules that the retrosynthesis model deems unsynthesizable (no route returned), synthesizable but not necessarily containing an enforced block, and synthesizable with the presence of an EBB, respectively. Note that Solved (Enforced) is a much more challenging metric than just synthesizable, which previous work has shown is directly learnable^22^. We further report *N* as the number of replicates out of ten seeds where Solved (Enforced) > 0, and the mean and s.d. for the number of unique enforced blocks, denoting how many unique EBBs are in the routes for the Solved (Enforced) molecules. Next, we pool all Solved (Enforced) molecules, de-duplicate and report the mean and s.d. of the number of reaction steps. Among the generated molecules that satisfy constrained synthesizability, we further assess their property profiles and diversity. Accordingly, we report the mean and s.d. of the DSs, QED values and ligand efficiency (DS divided by the number of heavy atoms). To assess diversity, we report the internal diversity (IntDiv1)^34^, #Circles^64^ and the number of unique Bemis–Murcko scaffolds (#Scaffolds)^65^. Both IntDiv1 and #Circles use Morgan fingerprints with radius = 2 and nBits = 1,024. IntDiv1 is defined as 1 − (average pair-wise Tanimoto similarity of the generated set). #Circles is defined as the largest subset of generated molecules where all molecules are dissimilar to one another by a distance metric and threshold (0.75 in this work). Accordingly, all molecules in the subset are <0.25 Tanimoto similarity.

## Supplementary information

Supplementary Information Data preprocessing, model training, computation details, TANGO development and ablations, and example synthesis routes.

Peer Review File

## Source data

Source Data Fig. 2 SMILES, QED and DS of the displayed molecules.

Source Data Fig. 3 Raw metrics and property values.

Source Data Fig. 4 Synthesis route of the generated molecules for the COX-2 case study.

Source Data Table 1 Raw data for Table 1.

Source Data Table 2 Raw data for Table 2.

Source Data Extended Data Table 1 Raw property metrics for the molecules corresponding to Table 1.

Source Data Extended Data Table 2 Raw property metrics for the molecules corresponding to Table 2.

Source Data Extended Data Table 3 Raw synthesizability metrics for the COX-2 case study.

Source Data Extended Data Table 4 Raw synthesizability metrics for the COX-2 case study.

## Figures and Tables

**Extended Data Table 1:**
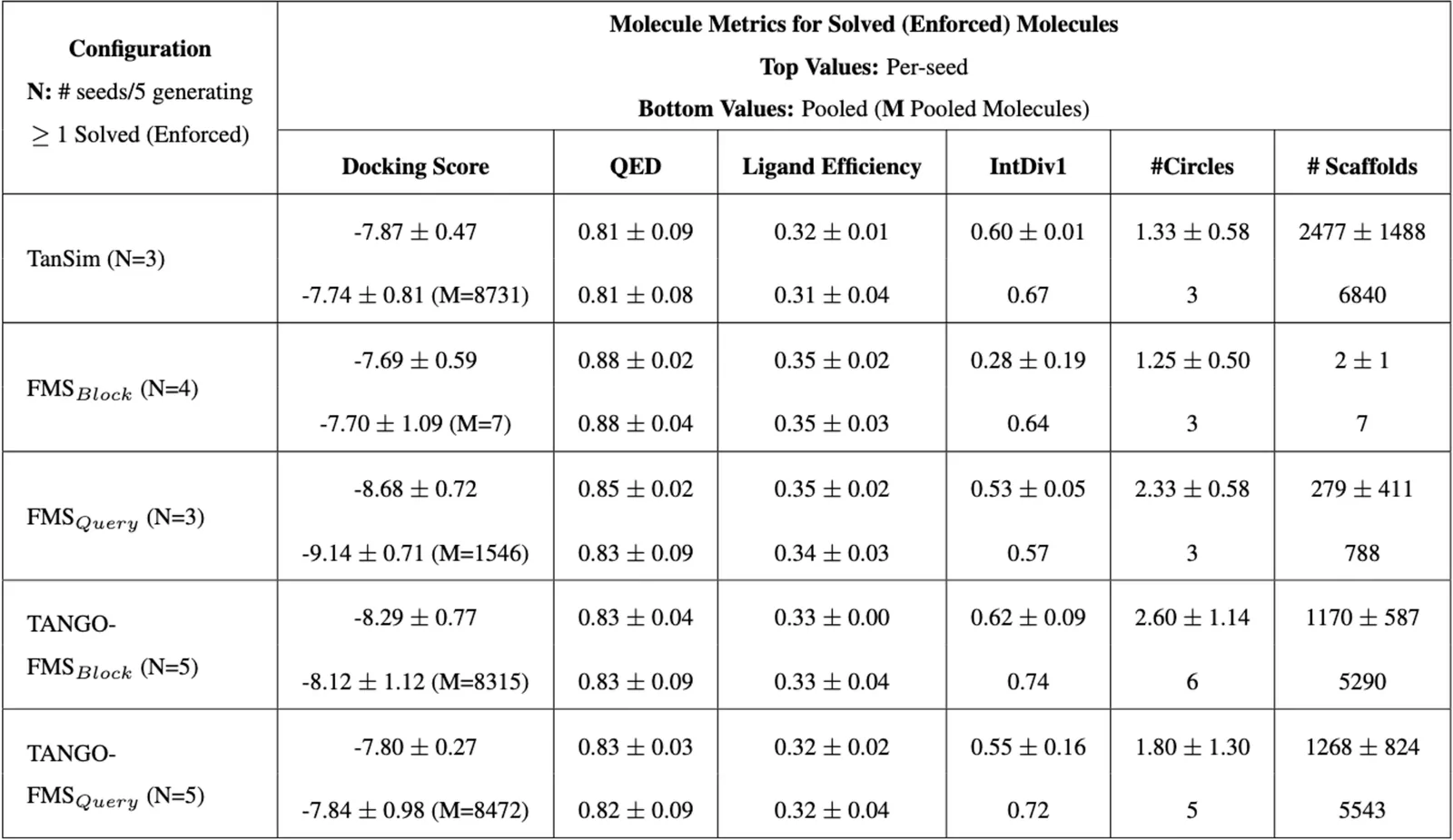
Reward function experiments: Molecular property and diversity metrics across different reward functions: Tanimoto Similarity (**TanSim**), Fuzzy Matching Substructure (FMS) normalized by the enforced building block (FMS_*Block*_) or query molecule (FMS_*Query*_), and TANGO-FMS_*Block*_ or TANGO-FMS_*Query*_, both with 0.50 FMS and 0.50 TanSim weighting. All experiments were with 100 enforced building blocks. The number of seeds (out of 5) with at least 1 generated molecule satisfying constrained synthesizability is reported with **N**. Metrics (for constrained synthesizable molecules) are reported in two settings: (1) Per-seed: Averaged across seeds (2) Pooled: Pooling all molecules across seeds and de-duplicating. The number of pooled, de-duplicated molecules is denoted by **M**

.

Source data

**Extended Data Table 2:**
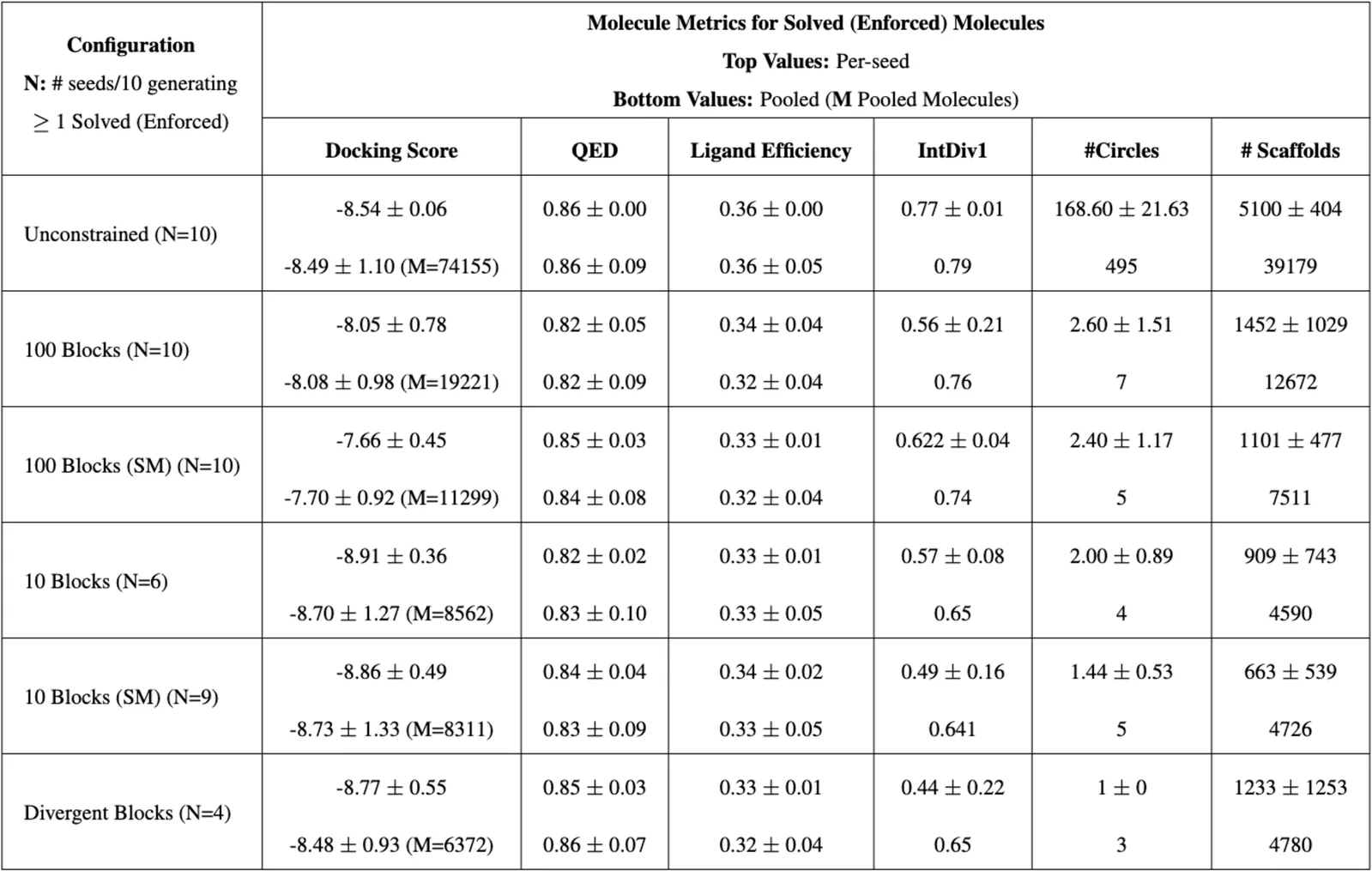
Constrained synthesizability experiments: Molecular property and diversity metrics with the TANGO reward function (0.50 FMS_*Block*_ and 0.50 TanSim weighting). ‘SM’ denotes starting-material constrained. ‘Unconstrained’ denotes the experiment without enforcing building blocks, as a comparison. The number of seeds (out of 10) with at least 1 generated molecule satisfying constrained synthesizability is reported with **N**. Metrics (for constrained synthesizable molecules) are reported in two settings: (1) Per-seed: Averaged across seeds (2) Pooled: Pooling all molecules across seeds and de-duplicating. The number of pooled, de-duplicated molecules is denoted by **M**. ^a^ There is no notion of ‘enforced blocks’ in the ‘Unconstrained’ setting, so the metrics are reported for ‘Solved’ molecules

.

Source data

**Extended Data Table 3:**
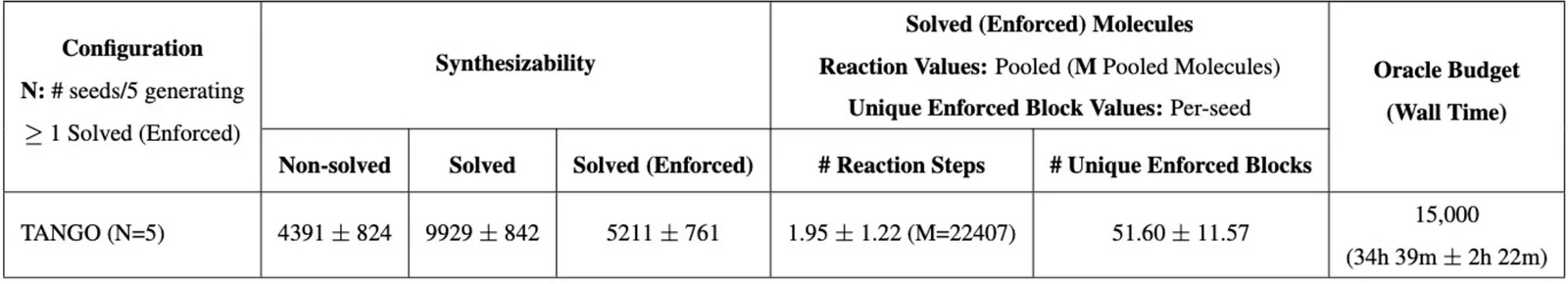
COX2 case study to enforce the use of phenylsulfonamide-containing blocks using the TANGO reward function (0.50 FMS_*Block*_ and 0.50 TanSim weighting): Synthesizability metrics. The number of seeds (out of 5) with at least 1 generated molecule satisfying constrained synthesizability is reported with **N**. Metrics (for constrained synthesizable molecules) are reported in two settings: (1) Per-seed: Averaged across seeds (2) Pooled: Pooling all molecules across seeds and de-duplicating. The number of pooled, de-duplicated molecules is denoted by **M**. Note the docking scores here are with ‘gnina’ instead of ‘QuickVina2-GPU’ used in the previous experiments

.

Source data

**Extended Data Table 4:**
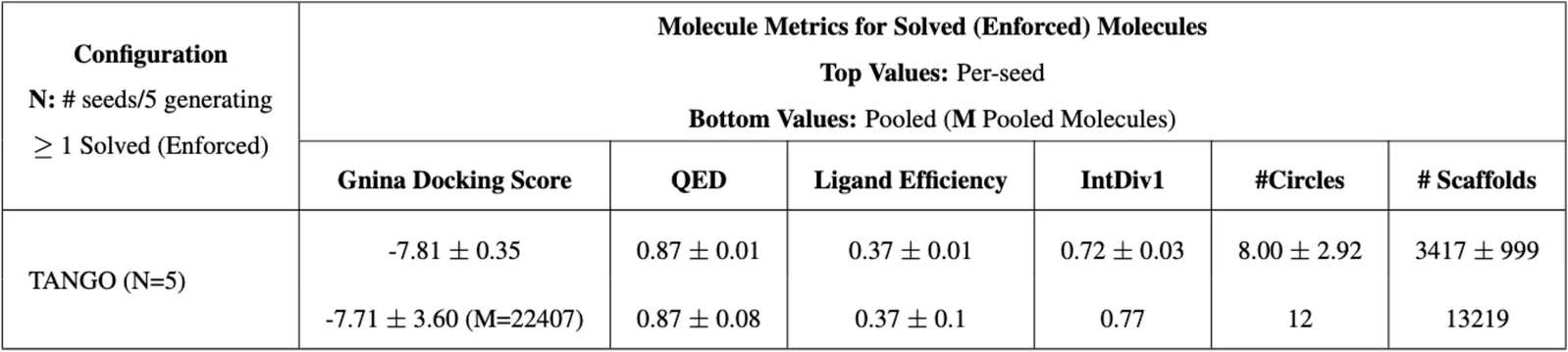
COX2 case study to enforce the use of phenylsulfonamide-containing blocks using the TANGO reward function (0.50 FMS_*Block*_ and 0.50 TanSim weighting): Molecular property and diversity metrics. The number of seeds (out of 5) with at least 1 generated molecule satisfying constrained synthesizability is reported with **N**. Metrics (for constrained synthesizable molecules) are reported in two settings: (1) Per-seed: Averaged across seeds (2) Pooled: Pooling all molecules across seeds and de-duplicating. The number of pooled, de-duplicated molecules is denoted by **M**. Note the docking scores here are with ‘gnina’ instead of ‘QuickVina2-GPU’ used in the previous experiments

## Data Availability

The building-block stocks used in this work are deposited in figshare at https://doi.org/10.6084/m9.figshare.27225483 (ref. ^66^). Source data are available for Figs. 2–4, Tables 1 and 2 and Extended Data Tables 1–4. Source data are provided with this paper.

## Code availability

The code is available at https://github.com/schwallergroup/saturn and deposited in figshare at https://doi.org/10.6084/m9.figshare.27225483 (ref. ^66^). Instructions are provided in the repository to reproduce the experiments.

## References

[1] Gao, W. & Coley, C. W. The synthesizability of molecules proposed by generative models. *J. Chem. Inf. Model.* **60**, 5714–5723 (2020). 10.1021/acs.jcim.0c00174 32250616

[2] Stanley, M. & Segler, M. Fake it until you make it? Generative de novo design and virtual screening of synthesizable molecules. *Curr. Opin. Struct. Biol.* **82**, 102658 (2023). 10.1016/j.sbi.2023.102658 37473637

[3] Du, Y. et al. Machine learning-aided generative molecular design. *Nat. Mach. Intell.* **6**, 589–604 (2024).

[4] Coley, C. W. et al. Autonomous discovery in the chemical sciences part I: progress. *Angew. Chem. Int. Ed.* **59**, 22858–22893 (2020). 10.1002/anie.201909987 31553511

[5] Coley, C. W. et al. Autonomous discovery in the chemical sciences part II: outlook. *Angew. Chem. Int. Ed.* **59**, 23414–23436 (2020). 10.1002/anie.201909989 31553509

[6] Tom, G. et al. Self-driving laboratories for chemistry and materials science. *Chem. Rev.* **124**, 9633–9732 (2024). 10.1021/acs.chemrev.4c00055 PMC11363023 39137296

[7] Sin, J. W. et al. Highly parallel optimisation of chemical reactions through automation and machine intelligence. *Nat. Commun.* **16**, 6464 (2025). 10.1038/s41467-025-61803-0 PMC12255721 40651950

[8] Neeser, R. M. et al. FSscore: a personalized machine learning-based synthetic feasibility score leveraging human expertise. *Chem. Methods* **4**, e202400024 (2024).

[9] Bradshaw, J. et al. A model to search for synthesizable molecules. In *Proc. 33rd International Conference on Neural Information Processing Systems* 7937–7949 (Curran Associates Inc., 2019).

[10] Bradshaw, J. et al. Barking up the right tree: an approach to search over molecule synthesis DAGs. *Adv. Neural Inf. Process. Syst* **33**, 6852–6866 (2020).

[11] Gao, W. et al. Amortized tree generation for bottom-up synthesis planning and synthesizable molecular design. In *Proc. 10th International Conference on Learning Representations* (ICLR, 2022); https://openreview.net/forum?id=FRxhHdnxt1

[12] Koziarski, M. et al. RGFN: synthesizable molecular generation using GFlowNets. In *Proc. 38th International Conference on Neural Information Processing Systems* 46908–46955 (Curran Associates Inc., 2024).

[13] Cretu, M. et al. SynFlowNet: towards molecule design with guaranteed synthesis pathways. In *Proc. 13th International Conference on Learning Representations* 47101–47126 (ICLR, 2025).

[14] Seo, S. et al. Generative flows on synthetic pathway for drug design. In *Proc. 13th International Conference on Learning Representations* 14029–14060 (ICLR, 2025).

[15] Gao, W., Luo, S. & Coley, C. W. Generative AI for navigating synthesizable chemical space. *Proc. Natl Acad. Sci. USA* **122**, e2415665122 (2025). 10.1073/pnas.2415665122 PMC12541314 41052333

[16] Liu, B. et al. Retrosynthetic reaction prediction using neural sequence-to-sequence models. *ACS Cent. Sci.* **3**, 1103–1113 (2017). 10.1021/acscentsci.7b00303 PMC5658761 29104927

[17] Segler, M. H. & Waller, M. P. Neural-symbolic machine learning for retrosynthesis and reaction prediction. *Chem. Eur. J.* **23**, 5966–5971 (2017). 10.1002/chem.201605499 28134452

[18] Coley, C. W. et al. Computer-assisted retrosynthesis based on molecular similarity. *ACS Cent. Sci.* **3**, 1237–1245 (2017). 10.1021/acscentsci.7b00355 PMC5746854 29296663

[19] Segler, M. H. et al. Planning chemical syntheses with deep neural networks and symbolic AI. *Nature* **555**, 604–610 (2018). 10.1038/nature25978 29595767

[20] Schwaller, P. et al. Predicting retrosynthetic pathways using transformer-based models and a hyper-graph exploration strategy. *Chem. Sci.* **11**, 3316–3325 (2020). 10.1039/c9sc05704h PMC8152799 34122839

[21] Shields, J. D. et al. AiZynth impact on medicinal chemistry practice at AstraZeneca. *RSC Med. Chem.* **15**, 1085–1095 (2024). 10.1039/d3md00651d PMC11042116 38665822

[22] Guo, J. & Schwaller, P. Directly optimizing for synthesizability in generative molecular design using retrosynthesis models. *Chem. Sci.* **16**, 6943–6956 (2025). 10.1039/d5sc01476j PMC11927497 40123687

[23] Vollmann, D. J. et al. Emerging concepts in the semisynthetic and mutasynthetic production of natural products. *Curr. Opin. Biotechnol.* **77**, 102761 (2022). 10.1016/j.copbio.2022.102761 35917622

[24] Li, L. et al. Divergent strategy in natural product total synthesis. *Chem. Rev.* **118**, 3752–3832 (2018). 10.1021/acs.chemrev.7b00653 29516724

[25] Granda, J. M. et al. Controlling an organic synthesis robot with machine learning to search for new reactivity. *Nature* **559**, 377–381 (2018). 10.1038/s41586-018-0307-8 PMC6223543 30022133

[26] Wołos, A. et al. Synthetic connectivity, emergence, and self-regeneration in the network of prebiotic chemistry. *Science* **369**, eaaw1955 (2020). 10.1126/science.aaw1955 32973002

[27] Ża̧dło-Dobrowolska, A. et al. Computational synthesis design for controlled degradation and revalorization. *Nat. Synth.* **3**, 643–654 (2024).

[28] Wołos, A. et al. Computer-designed repurposing of chemical wastes into drugs. *Nature* **604**, 668–676 (2022). 10.1038/s41586-022-04503-9 35478240

[29] Shanks, B. H. & Keeling, A. P. Bioprivileged molecules: creating value from biomass. *Green Chem.* **19**, 3177–3185 (2017).

[30] Johnson, A. P. et al. Starting material oriented retrosynthetic analysis in the LHASA program. 1. General description. *J. Chem. Inf. Comput. Sci.* **32**, 411–417 (1992).

[31] Yu, Y. et al. GRASP: navigating retrosynthetic planning with goal-driven policy. *Adv. Neural Inf. Process. Syst* **35**, 10257–10268 (2022).

[32] Yu, K. et al. Double-ended synthesis planning with goal-constrained bidirectional search. *Adv. Neural Inf. Process. Syst* **37**, 112919–112949 (2024).

[33] Grzybowski, B. A. et al. Chematica: a story of computer code that started to think like a chemist. *Chem* **4**, 390–398 (2018).

[34] Benhenda, M. ChemGAN challenge for drug discovery: can AI reproduce natural chemical diversity? Preprint at https://arxiv.org/abs/1708.08227 (2017).

[35] Guo, J. & Schwaller, P. Saturn: Sample-efficient generative molecular design using memory manipulation. Preprint at https://arxiv.org/abs/2405.17066 (2024). 10.1021/jacsau.4c00066 PMC11200228 38938817

[36] Bickerton, G. R. et al. Quantifying the chemical beauty of drugs. *Nat. Chem.* **4**, 90–98 (2012). 10.1038/nchem.1243 PMC3524573 22270643

[37] Castellino, N. J. et al. Late-stage functionalization for improving drug-like molecular properties. *Chem. Rev.* **123**, 8127–8153 (2023). 10.1021/acs.chemrev.2c00797 37285604

[38] McInnes, L. et al. UMAP: uniform manifold approximation and projection for dimension reduction. *J. Open Source Softw.* **3**, 861 (2018).

[39] Deb, P. K. et al. in *Nonsteroidal Anti-Inflammatory Drugs* (ed. Al-kaf, A. G. A.) Ch. 6 (IntechOpen, 2017).

[40] Sun, S. X. et al. Withdrawal of COX-2 selective inhibitors rofecoxib and valdecoxib: impact on NSAID and gastroprotective drug prescribing and utilization. *Curr. Med. Res. Opin.* **23**, 1859–1866 (2007). 10.1185/030079907X210561 17605893

[41] Kurumbail, R. G. et al. Structural basis for selective inhibition of cyclooxygenase-2 by anti-inflammatory agents. *Nature* **384**, 644–648 (1996). 10.1038/384644a0 8967954

[42] Sterling, T. & Irwin, J. J. ZINC 15—ligand discovery for everyone. *J. Chem. Inf. Model.* **55**, 2324–2337 (2015). 10.1021/acs.jcim.5b00559 PMC4658288 26479676

[43] McNutt, A. T. et al. GNINA 1.0: molecular docking with deep learning. *J. Cheminform.* **3**, 43 (2021). 10.1186/s13321-021-00522-2 PMC8191141 34108002

[44] Trott, O. & Olson, A. J. AutoDock Vina: improving the speed and accuracy of docking with a new scoring function, efficient optimization, and multithreading. *J. Comput. Chem.* **31**, 455–461 (2010). 10.1002/jcc.21334 PMC3041641 19499576

[45] NameRxn v.3.7.3 (NextMove Software, 2024).

[46] Molga, K. et al. The logic of translating chemical knowledge into machine-processable forms: a modern playground for physical–organic chemistry. *React. Chem. Eng.* **4**, 1506–1521 (2019).

[47] Genheden, S. et al. AiZynthFinder: a fast, robust and flexible open-source software for retrosynthetic planning. *J. Cheminform.* **12**, 70 (2020). 10.1186/s13321-020-00472-1 PMC7672904 33292482

[48] Saigiridharan, L. et al. AiZynthFinder 4.0: developments based on learnings from 3 years of industrial application. *J. Cheminform.* **16**, 57 (2024). 10.1186/s13321-024-00860-x PMC11112899 38778382

[49] Maziarz, K. et al. Re-evaluating retrosynthesis algorithms with Syntheseus. *Faraday Discuss.* **31**, 568–586 (2025). 10.1039/d4fd00093e 39485491

[50] Tu, Z. et al. ASKCOS: an open source software suite for synthesis planning. *Acc. Chem. Res.* **58**, 1764–1775 (2025). 10.1021/acs.accounts.5c00155 PMC12140107 40397546

[51] Ng, A. Y. et al. Policy invariance under reward transformations: theory and application to reward shaping. In *ICML ’99: Proc. Sixteenth International Conference on Machine Learning* (eds Bratko, I. & Dzeroski, S.) 278–287 (Morgan Kaufmann, 1999).

[52] Zhang, Y. et al. Evolutionary retrosynthetic route planning. *IEEE Comput. Intell. Mag.* **19**, 58–72 (2024).

[53] Weininger, D. SMILES, a chemical language and information system. 1. Introduction to methodology and encoding rules. *J. Chem. Inf. Comput. Sci.* **28**, 31–36 (1988).

[54] Gu, A. & Dao, T. Mamba: linear-time sequence modeling with selective state spaces. In *First**Conference on Language Modeling* (COLM, 2024); https://openreview.net/forum?id=tEYskw1VY2

[55] Bjerrum, E. J. SMILES enumeration as data augmentation for neural network modeling of molecules. Preprint at https://arxiv.org/abs/1703.07076 (2017).

[56] Lin, L.-J. Self-improving reactive agents based on reinforcement learning, planning and teaching. *Mach. Learning* **8**, 293–321 (1992).

[57] Guo, J. & Schwaller, P. Augmented Memory: sample-efficient generative molecular design with reinforcement learning. *JACS Au* **4**, 2160–2172 (2024). 10.1021/jacsau.4c00066 PMC11200228 38938817

[58] Kim, S. et al. PubChem 2023 update. *Nucl. Acids Res.* **51**, D1373–D1380 (2023). 10.1093/nar/gkac956 PMC9825602 36305812

[59] Sacha, M. et al. Molecule Edit Graph Attention Network: modeling chemical reactions as sequences of graph edits. *J. Chem. Inf. Model.* **61**, 3273–3284 (2021). 10.1021/acs.jcim.1c00537 34251814

[60] Chen, B. et al. Retro*: learning retrosynthetic planning with neural guided A* search. *Proc. Mach. Learning Res*. **119**, 1608–1616 (2020).

[61] Alhossary, A. et al. Fast, accurate, and reliable molecular docking with QuickVina 2. *Bioinformatics* **31**, 2214–2216 (2015). 10.1093/bioinformatics/btv082 25717194

[62] Tang, S. et al. Vina-GPU 2.1: towards further optimizing docking speed and precision of AutoDock Vina and its derivatives. *IEEE/ACM Trans. Comput. Biol. Bioinform.* **21**, 2382–2393 (2024). 10.1109/TCBB.2024.3467127 39320991

[63] Mabanglo, M. F. et al. Potent ClpP agonists with anticancer properties bind with improved structural complementarity and alter the mitochondrial N-terminome. *Structure* **31**, 185–200 (2023). 10.1016/j.str.2022.12.002 PMC9898158 36586405

[64] Xie, Y. et al. How much space has been explored? Measuring the chemical space covered by databases and machine-generated molecules. In *Proc. 11th International Conference on Learning Representations* (ICLR, 2023); https://openreview.net/pdf?id=Yo06F8kfMa1

[65] Bemis, G. W. & Murcko, M. A. The properties of known drugs. 1. Molecular frameworks. *J. Med. Chem.* **39**, 2887–2893 (1996). 10.1021/jm9602928 8709122

[66] Guo, J. TANGO paper building blocks and code-base. *figshare*10.6084/m9.figshare.27225483 (2025).

