# Supplementary material for: TANGO: direct optimization of constrained synthesizability for generative molecular design
Source: Nat Comput Sci. 2026 Mar 3;6(3):260–70. doi: 10.1038/s43588-026-00959-1 (PMC13658032; doi:10.1038/s43588-026-00959-1)
Supplement: Supplementary file 1 [file 43588_2026_959_MOESM1_ESM.pdf]

nature computational science

Supplementary information

https://doi.org/10.1038/s43588-026-00959-1

TANGO: direct optimization of constrained synthesizability for generative molecular design

In the format provided by the authors and unedited

Supplementary Information for

It Takes Two to Tango: Directly Optimizing for *Constrained* Synthesizability in Generative Molecular Design

Jeff Guo[1,2*] and Philippe Schwaller[1,2*]

[1] *École Polytechnique Fédérale de Lausanne (EPFL)*

[2] *National Centre of Competence in Research (NCCR) Catalysis*

[*] *Correspondence to {jeff.guo, philippe.schwaller}@epfl.ch*

Contents

1 Supplementary Section 1 **1**

2 Supplementary Section 2 **3**

2.1 Supplementary Section 2.1 3

2.2 Supplementary Section 2.2 3

3 Supplementary Section 3 **5**

3.1 Supplementary Section 3.1 5

3.2 Supplementary Section 3.2 5

3.3 Supplementary Section 3.3 5

4 Supplementary Section 4 **6**

4.1 Supplementary Section 4.1 9

4.2 Supplementary Section 4.2 13

4.3 Supplementary Section 4.3 15

4.4 Supplementary Section 4.4 18

4.5 Supplementary Section 4.5 22

4.6 Supplementary Section 4.6 25

4.7 Supplementary Section 4.7 27

4.8 Supplementary Section 4.8 31

4.9 Supplementary Section 4.9 33

4.10 Supplementary Section 4.10 34

4.11 Supplementary Section 4.11 36

5 Supplementary Section 5 **38**

5.1 Supplementary Section 5.1 38

5.2 Supplementary Section 5.2 38

5.3 Supplementary Section 5.3 39

6 Supplementary Section 6 **40**

1 Supplementary Section 1

Note, we have re-used this section in our subsequent work [1] as the pre-training details are shared.

This section contains the full data pre-processing and pre-training pipeline starting from the raw PubChem which was downloaded from https://ftp.ncbi.nlm.nih.gov/pubchem/Compound/Extras/. The exact file is "CID-SMILES.gz".

The exact pre-processing steps along with the SMILES remaining after each step are:

1. Raw PubChem - 118,563,810
2. De-duplication - 118,469,904
3. Standardization (charge and isotope handling) based on https://github.com/MolecularAI/ReinventCommunity/blob/master/notebooks/Data_Preparation.ipynb. All SMILES that could not be parsed by RDKit were removed - 109,128,315
4. Tokenize all SMILES based on REINVENT's tokenizer: https://github.com/MolecularAI/reinvent-models/blob/main/reinvent_models/reinvent_core/models/vocabulary.py
5. Keep SMILES $\leq$ 80 tokens, 150 $\leq$ molecular weight $\leq$ 650, number of heavy atoms $\leq$ 40, number of rings $\leq$ 8, Size of largest ring $\leq$ 8, longest aliphatic carbon chain $\leq$ 4 - 97,667,549
6. Removed SMILES containing the following tokens (due to undesired chemistry, low token frequency, and redundancy): [Br+2], [Br+3], [Br+], [C+], [C-], [CH+], [CH-], [CH2+], [CH2-], [CH2], [CH], [C], [Cl+2], [Cl+3], [Cl+], [ClH+2], [ClH2+2], [ClH3+3], [N-], [N@+], [N@@+], [NH+], [NH-], [NH2+], [NH3+], [NH], [N], [O+], [OH+], [OH2+], [O], [S+], [S-], [S@+], [S@@+], [S@@], [S@], [SH+], [SH-], [SH2], [SH4], [SH], [S], [c+], [c-], [cH+], [cH-], [c], [n+], [n-], [nH+], [nH], [o+], [s+], [sH+], [sH-], [sH2], [sH4], [sH], [s] - **88,618,780**

The final vocabulary contained 35 tokens (2 extra tokens were added, indicating <START> and <END>) and carbon stereochemistry tokens were kept. Saturn [2] uses the Mamba [3] architecture and we used the default hyperparameters in the code-base. With the vocabulary size of 35,

the model has 5,265,408 parameters. Saturn was pre-trained for 5 steps, with each step consisting of a full pass through the dataset. The model was pre-trained on a workstation with an NVIDIA RTX 3090 GPU and AMD Ryzen 9 5900X 12-Core CPU. The pre-training parameters were:

1. Training steps = 5
2. Seed = 0
3. Batch size = 512
4. Learning rate = 0.0001
5. Randomize [4] every batch of SMILES

Relevant metrics of the pre-trained model (final model checkpoint) are:

1. Average negative log-likelihood (NLL) = 30.914
2. Validity (10k) = 98.74%
3. Uniqueness (10k) = 98.73%
4. Wall time = 106 hours (takes a relatively long time, though we only used 1 GPU for training. Pre-training also only needs to be done once.)

2 Supplementary Section 2

This section contains details on the retrosynthesis model, the commercial building blocks, and the enforced building blocks.

2.1 Supplementary Section 2.1

In this work, we use Syntheseus [5] (benchmark platform and wrapper around retrosynthesis models and search algorithms) to run retrosynthesis. We integrate Syntheseus into Saturn [2] and run the MEGAN [6] single-step model with the Retro* [7] search algorithm. In the Syntheseus work, the authors standardize and benchmark many retrosynthesis models and configurations, reporting the inference time and accuracy (across various metrics). We chose MEGAN because it has the fastest inference time, although the top-k accuracies were lower than other models. We note that top-k single-step accuracy does not necessarily equate to better performance on multi-step retrosynthesis. Faster inference time allowed us to iterate experiments and hypotheses faster and is the main reason we chose MEGAN. Our framework is model-agnostic and any retrosynthesis model could be used in place of MEGAN. All MEGAN hyperparameters were tuned by the Syntheseus authors and we use them as is.

2.2 Supplementary Section 2.2

All retrosynthesis models require commercial building blocks, B. In this work, we use the 'Fragment' and 'Reactive' sub-sets of ZINC [8], equating to 17,721,980 building blocks. These sub-sets were obtained from the commercial building block stock used in AiZynthFinder [9, 10]. Next, we consider two sets of enforced building blocks, $B_{enf-10} \subset B_{enf-100} \subset B$. The enforced building block sets (10 or 100) are sub-sets of B and were randomly sampled following the criteria:

1. 150 $<$ molecular weight $<$ 200
2. No aliphatic carbon chains longer than 3
3. Exclude charged building blocks
4. If rings are present, enforce size $\in \{5, 6\}$

5. All building blocks must contain at least one nitrogen, oxygen, or sulfur atom

The criteria we defined are based on enforcing building blocks that are "simple, common, and relevant for drug-like molecules". While there is an inherent bias here, we emphasize that our TANGO framework is general and the set of enforced building blocks can be freely changed. Finally, we want to highlight an important implication when considering the commercial building blocks, B, and the generative model. Due to intentional data pre-processing of PubChem, which was used to pre-train Saturn, the generative model cannot generate all the atom types present in B. The specific atom types are phosphorus and silicon. We removed these atoms due to their seldom presence in "drug-like" molecules (although phosphorus is common in pro-drugs). The effect of this is that some commercial building blocks are not relevant, but we did not purge these and used the ZINC sub-sets as is. Similar to the enforced building blocks set, the set of commercial building blocks can also freely be changed. The sets of enforced building blocks are provided in the code-base.

3 Supplementary Section 3

3.1 Supplementary Section 3.1

Every experiment (except pre-training Saturn) was run on a cluster equipped with NVIDIA L40S GPUs. As we used a SLURM queuing system, many jobs could be allocated the same GPU to run simultaneously. This makes the wall time for each individual run slower, but the total time to finish experiments is faster. We report the wall times as is.

3.2 Supplementary Section 3.2

Here, we report the wall time for Tanimoto similarity (TanSim) and Maximum Common Substructure (MCSS) in our framework for varying enforced building block set sizes. We take the commercial stock used in this work and randomly sample $\{1, 10, 100, 1000, 10000, 100000\}$ building blocks with molecular weight > 200 Da. Next, we arbitrarily sample a 3-step synthesis route with 5 molecule nodes, excluding the generated molecule (Fig. 2).

Fig. 2 shows the wall time required to compute the TanSim and MCSS comparing these 5 molecule nodes to varying enforced building block sets. We further report multi-threading (4) results and also `x64` results which multiply the wall time by 64. This mimics the batch size of 64 used, which means the generative model samples 64 molecules at every generation epoch. Note that in practice, 64 is seldom, if ever observed, because this requires all 64 molecules to have a synthesis route (retrosynthesis model solvable). Lastly, we investigated enforced building block set sizes up to 100,000 but we do not envision this to be a common use case as that is approaching the size of some commercial stocks. In that scenario, the entire commercial stock is "enforced", which is equivalent to normal retrosynthesis model solvability.

3.3 Supplementary Section 3.3

Saturn expected every property to be optimized to have a normalized reward $\in [0, 1]$. TANGO and QED are already by design normalized but QuickVina2-GPU docking needs to be reward shaped. This is done by the shaping function shown here.

4 Supplementary Section 4

In this section, we present the systematic development of TANGO, all ablation studies, and additional results. We first illustrate how the TANGO reward is derived, given a solved synthesis route (Fig. 1. Accordingly, Algorithm 1 shows step-by-step the TANGO computation.

The remaining section will be divided sequentially into sub-sections detailing our hypotheses, the experiments we ran to study them, and the observations we made during the development of TANGO. All development experiments were run across 5 seeds (0-4 inclusive) while main result experiments were run across 10 seeds (0-9 inclusive). This information will be noted. The MPO objective is:

1. **Minimize QuickVina2-GPU-2.1** [11–13] docking scores against ATP-dependent Clp protease proteolytic subunit (ClpP) [14] (implicated in cancer)

2. **Maximize QED** [15]

3. **Constrained Synthesizable**, as deemed by the MEGAN [6] retrosynthesis model coupled with Retro* search [7]

Next, throughout TANGO development, we change the hyperparameters of Saturn, which directly control for the exploration-exploitation trade-off. We briefly summarize the key hyperparameters and their effect:

1. **Batch Size:** Lower is more exploitative

2. **Augmentation Rounds:** Higher is more exploitative

Algorithm 1: TANGO Reward Calculation

```
Input: G(M, R) // Synthesis graph of generated molecule
  B_enf // The set of enforced building blocks
  maxdepth // Graph depth of terminal leaf nodes
  enforcestart ∈ {True, False} // Starting material constraint
Output: reward ∈ ℝ // TANGO reward
// Initialize reward
reward ← 0
// Traverse all non-root nodes in the synthesis graph
foreach m ∈ G(M, R) such that depth(m) > 0 do
    // Enforce start constraint if applicable
    if enforcestart then
        if depth(m) ≠ maxdepth then
            continue
        end
    end
    reward ← max(reward, TANGONodeReward(m, B_enf))
end
Function TANGONodeReward(query_node, B_enf):
    node_reward ← 0
    // Evaluate all enforced building blocks
    foreach EBB ∈ B_enf do
        TanSim ← ComputeTanimotoSimilarity(query_node, EBB)
        FMS ← ComputeFMS_Block(query_node, EBB)
        // Fallback to FMS_Query
        if FMS = 1 and query_node ≠ EBB then
            FMS ← ComputeFMS_Query(query_node, EBB)
        end
        block_reward ← TanSim · 0.5 + FMS · 0.5
        node_reward ← max(node_reward, block_reward)
    end
    return node_reward
return reward
```

Finally, for all sets of experiments, we report metrics averaged across either 5 (0-4 inclusive) or 10 (0-9 inclusive) seeds and also for the entire pooled, de-duplicated set. The number of seeds will be explicitly noted. The metrics are:

1. **Non-solved:** Number of generated molecules that do not have a solved synthesis route
2. **Solved:** Number of generated molecules that have any synthesis route
3. **Solved (Enforced):** Number of generated molecules that have a synthesis route **with at least 1 enforced building block**
4. **Oracle Budget:** Number of oracle calls permitted
5. **Wall Time:** Compute time for the run
6. **Docking Score**: QuickVina2-GPU docking scores [11–13]
7. **QED**: QED values [15]
8. **Ligand Efficiency**: QuickVina2-GPU docking scores divided by the number of heavy atoms of the generated molecule
9. **IntDiv1**: Internal Diversity using Morgan fingerprints (radius=2, nBits=1024) [21]
10. **#Circles**: "Sphere packing" [22]
11. **# Scaffolds**: Number of Bemis-Murcko scaffolds [23]

Constrained Synthesizability denotes either starting material constrained (enforced building blocks appearing at the max depth nodes in the synthesis graph), intermediate-constrained (enforced building blocks appearing anywhere in the synthesis graph), or divergent synthesis (enforced non-commercial building blocks appearing anywhere in the synthesis graph). This information will be explicitly noted. Finally, for brevity, we will write "synthesizable" to mean synthesizable, as deemed by the MEGAN retrosynthesis model.

4.1 Supplementary Section 4.1

The starting point of TANGO development drew inspiration from [16, 17] which used TanSim for retrosynthesis problems. We hypothesized that TanSim alone is insufficient to inform chemical reactivity. Therefore, very initial experiments tried to "filter" nodes by matching for functional groups. Specifically, for every molecule generated that was synthesizable, there is a corresponding synthesis graph whose nodes are every intermediate molecule. The very first reward function traverses these nodes and computes the max TanSim to the set of enforced building blocks, provided that the node overlaps 75% of the functional groups with at least one of the enforced building blocks, and returns this as the reward. With this initial reward formulation, we used Saturn's [2] default hyperparameters of batch size 16 and 10 augmentation rounds. These parameters make the model perform local sampling in chemical space aggressively. We had run this experiment across 5 seeds (0-4 inclusive) with an oracle budget of 3,000 and only one seed was successful in generating some synthesizable molecules with the enforced building blocks. All seeds showed some learning, in that the average TanSim of the synthesis graphs to the enforced building blocks was increasing (though it always stagnated). At the time, this was highly irreproducible, considering only 1/5 runs were successful. However, these failed runs gave us sets of molecules possessing various Tanimoto similarity to the enforced building blocks which we used to investigate various reward shaping functions. Specifically, we took the set of all generated molecules from one of the seeds and partitioned all the molecules that were synthesizable into the following TanSim thresholds (to the enforced building blocks):

1. **Low:** $0.0 < \text{TanSim} < 0.2$ (N = 237)
2. **Med:** $0.2 \leq \text{TanSim} < 0.3$ (N = 438)
3. **Med-High:** $0.3 \leq \text{TanSim} < 0.4$ (N = 734)
4. **High:** $0.4 \leq \text{TanSim} < 0.5$ (N = 38)
5. **Very-High:** $0.5 \leq \text{TanSim} > 1.0$ (N = 712)

The **reward distributions** of these sets of molecules were visualized under different **reward formulations** (Fig. 4). All comparison are to the set of enforced building blocks:

1. **Functional Groups (FG):** Mean or max functional groups overlap

2. **Tanimoto Similarity (TanSim):** Mean or max Tanimoto Similarity

3. **Fuzzy Matching Substructure (FMS):** Mean or max fraction of atoms in the maximum matching substructure

4. **TANGO-FG:** Max TanSim + Mean FG

5. **TANGO-FMS:** Max TanSim + Max FMS

6. **TANGO-All:** Max TanSim + Mean FG + Max FMS

Based on Fig 4, **Max TanSim**, **Max FMS**, and **TANGO-FMS** are able to separate the partitioned TanSim intervals the best. These reward formulations are promising because they can distinguish between "closeness" to incorporating the enforced building blocks and enables a gradient for learning. It is important to know that this analysis has an explicit bias: we are assuming that TanSim does in fact equate to being "closer", since we partitioned the generated set based on this. However, this gave us the first hypotheses to work with.

Hypotheses:

1. The initial run with 1/5 successful seeds used Batch Size = 16 and Augmentation Rounds = 10. This is likely too exploitative. Try a more exploratory sampling behavior with **Batch Size = 32 and Augmentation Rounds = 5**.

2. Try the most promising reward functions: **Max TanSim**, **Max FMS**, and **TANGO-FMS**.

Fixed Parameters:

1. **Oracle Budget** = 3,000

2. **Batch Size** = 32

3. **Augmentation Rounds** = 5

4. **Enforced Building Blocks** = 100

Observations: Tables 1 and 2 show the results with the mean and standard deviation across 5 seeds (0-4 inclusive). We make the following observations:

1. All reward functions can yield successful runs.
2. TanSim is inconsistent with many unsuccessful seeds.
3. FMS is essentially unsuccessful.
4. TANGO-FMS yields the best average performance.

Supplementary Table 1: Results for Section 1: How can Constrained Synthesizability be made Learnable? Synthesizability metrics. The number of seeds (out of 5) with at least 1 generated molecule satisfying constrained synthesizability is reported with **N**. Metrics (for constrained synthesizable molecules) are reported in two settings: (1) Per-seed: Averaged across seeds (2) Pooled: Pooling all molecules across seeds and de-duplicating. The number of pooled, de-duplicated molecules is denoted by **M**.

**Configuration** **N:** # seeds/5 generating $\geq$ 1 Solved (Enforced)	**Synthesizability**			**Solved (Enforced) Molecules** **Reaction Values:** Pooled (**M** Pooled Molecules) **Unique Enforced Block Values:** Per-seed		**Oracle Budget (Wall Time)**
	**Non-solved**	**Solved**	**Solved (Enforced)**	**# Reaction Steps**	**# Unique Enforced Blocks**	
TanSim (N=2)	577 ± 32	2394 ± 33	333 ± 408	2.52 ± 1.30 (M=1627)	2.00 ± 0	3,000 (5h 3m ± 24m)
FMS (N=1)	649 ± 101	2326 ± 103	1 in total	1.00 ± 0.00 (M=1)	1.00 ± 0	3,000 (4h 50m ± 26m)
TANGO-FMS (N=5)	643 ± 23	2327 ± 32	476 ± 377	2.30 ± 1.27 (M=2236)	1.20 ± 0.40	3,000 (5h 28m ± 45m)

Supplementary Table 2: Results for Section 1: How can Constrained Synthesizability be made Learnable? Molecular property and diversity metrics. The number of seeds (out of 5) with at least 1 generated molecule satisfying constrained synthesizability is reported with **N**. Metrics (for constrained synthesizable molecules) are reported in two settings: (1) Per-seed: Averaged across seeds (2) Pooled: Pooling all molecules across seeds and de-duplicating. The number of pooled, de-duplicated molecules is denoted by **M**.

**Configuration** **N:** # seeds/5 generating $\geq$ 1 Solved (Enforced)	**Molecule Metrics for Solved (Enforced) Molecules** **Top Values:** Per-seed **Bottom Values:** Pooled (**M** Pooled Molecules)					
	**Docking Score**	**QED**	**Ligand Efficiency**	**IntDiv1**	**#Circles**	**# Scaffolds**
TanSim (N=2)	-7.36 ± 0.06	0.77 ± 0.02	0.31 ± 0.01	0.67 ± 0.06	2.50 ± 0.71	610 ± 60
	-7.36 ± 0.71 (M=1627)	0.77 ± 0.11	0.31 ± 0.04	0.70	3	1166
FMS (N=1)	-6.90 ± 0	0.92 ± 0	0.36 ± 0	0 ± 0	1 ± 0	1 ± 0
	-6.90 ± 0 (M=1)	0.92 ± 0	0.36 ± 0	0 ± 0	1 ± 0	1 ± 0
TANGO-FMS (N=5)	-8.13 ± 0.82	0.80 ± 0.05	0.33 ± 0.02	0.46 ± 0.26	1.20 ± 0.45	402 ± 388
	-7.76 ± 0.82 (M=2236)	0.80 ± 0.09	0.32 ± 0.04	0.67	4	1848

4.2 Supplementary Section 4.2

The FMS results from the previous section yielded false positives: A maximum reward (1.0) was assigned to many generated molecules, yet these molecules did not contain any of the enforced building blocks in its synthesis graphs. The reason for this is due to the asymmetric nature of the designed FMS reward function. We refer to Fig. 5. The FMS reward function computes the maximum substructure overlap and then divides the number of atoms in this overlap by the number of atoms in the enforced building block. From here on, we denote this $\textbf{FMS}_{Block}$ to indicate that the overlap is normalized by the enforced building block. Fig. 5 illustrates an edge case where the intermediate node contains the enforced building block as a substructure, but the overall structures do not exactly match. The result was that FMS_{Block} assigned a perfect reward (1.0). This edge case can be handled by an additional check for exact match, and returning the overlap normalized by the intermediate node (query molecule) instead. We denote this $\textbf{FMS}_{Query}$. This is one possible solution to avoid false positives, yet still reward the model since the overall node and enforced building block structures are similar. Therefore, for all **FMS** reward function results, we used this formulation.

However, we note that false positives only occur in the FMS_{Block} reward function case, as TANGO-FMS_{Block} cannot yield perfect reward. Since TANGO-FMS_{Block} is comprised of both FMS_{Block} and TanSim: even if FMS_{Block} is a false positive, TanSim cannot equal 1.0, and thus TANGO-FMS_{Block} cannot equal 1.0. We hypothesized that this false positive can actually be beneficial, as a perfect reward biases the model towards generating molecules that yield a synthesis graph with an intermediate node similar to an enforced building block. This exploitation behavior could be advantageous. In the next section, we investigate the exploration-exploitation trade-off of the generative model when using TANGO-FMS_{Block} as the reward function. Once we identified optimal hyperparameters, we performed an ablation study in the section after to quantitatively study the FMS_{Query} behavior. We sought to answer whether it is actually advantageous to return a "perfect reward (1.0)" for the FMS component in these situations?

We lastly note that a scenario where FMS_{Query} leads to false positives over FMS_{Block} is possible. Consider the case where the enforced building block is actually a superset of the query molecule, where the query molecule substructure is exactly in the enforced building block (perfect

overlap). Yet, the enforced building block has other additional heavy atoms. In this case, FMS_{Query} would return a perfect match and FMS_{Block} would not. Using the building block sets in this work, we did not observe this scenario.

4.3 Supplementary Section 4.3

The results from the first section identified TANGO-FMS as the most stable reward function. We further observed that FMS can lead to false positives and therefore introduced the FMS_{Block} and FMS_{Query} formulations. To avoid ambiguity, we now denote TANGO-FMS as TANGO-FMS_{Block}. From the previous section, we observed that during RL, reward improvement often stagnates.

Hypotheses:

1. Further relax the local sampling behavior of Saturn which may help reward stagnation

Fixed Parameters:

1. **Reward Function** = TANGO-FMS_{Block} (Later, this will just be referred to as TANGO)
2. **Oracle Budget** = 5,000
3. **Batch Size** = 32 or 64
4. **Augmentation Rounds** = Varied
5. **Enforced Building Blocks** = 100

Observations: Tables 3 and 4 show the results with the mean and standard deviation across 5 seeds (0-4 inclusive). We make the following observations:

1. Batch32, AR5 is the most successful but imposes a much longer wall time. This is due to Saturn's local sampling behavior at low batch sizes and high augmentation rounds.
2. Batch64, AR0 is essentially completely unsuccessful. This affirms that some degree of exploitation is beneficial.
3. Batch64, AR10 is somewhat inconsistent, suggesting too much exploitation.
4. Batch64, AR2 and AR5 are successful with the latter notably better, suggesting AR5 may be a good balance between exploration-exploitation.

Supplementary Table 3: Results for Section 2: Can we Circumvent Reward Stagnation? The number of seeds (out of 5) with at least 1 generated molecule satisfying constrained synthesizability is reported with **N**. Metrics (for constrained synthesizable molecules) are reported in two settings: (1) Per-seed: Averaged across seeds (2) Pooled: Pooling all molecules across seeds and de-duplicating. The number of pooled, de-duplicated molecules is denoted by **M**.

**Configuration** **N:** # seeds/5 generating $\geq$ 1 Solved (Enforced)	**Synthesizability**			**Solved (Enforced) Molecules** **Reaction Values:** Pooled (**M** Pooled Molecules) **Unique Enforced Block Values:** Per-seed		**Oracle Budget (Wall Time)**
	**Non-solved**	**Solved**	**Solved (Enforced)**	**# Reaction Steps**	**# Unique Enforced Blocks**	
Batch32, AR5 (N=4)	1125 $\pm$ 110	3807 $\pm$ 105	960 $\pm$ 872	2.76 $\pm$ 1.41 (M=4790)	1.00 $\pm$ 0	5,000 (11h 44m $\pm$ 1h 38m)
Batch64, AR10 (N=3)	954 $\pm$ 90	3943 $\pm$ 94	674 $\pm$ 707	2.76 $\pm$ 1.12 (M=3370)	2.00 $\pm$ 0.82	5,000 (5h 56m $\pm$ 25m)
Batch64, AR5 (N=4)	1029 $\pm$ 78	3928 $\pm$ 89	857 $\pm$ 530	2.16 $\pm$ 1.21 (M=4134)	1.25 $\pm$ 0.43	5,000 (4h 21m $\pm$ 18m)
Batch64, AR2 (N=4)	1175 $\pm$ 89	3829 $\pm$ 94	34 $\pm$ 48	1.60 $\pm$ 0.82 (M=167)	2.00 $\pm$ 1.00	5,000 (3h 20m $\pm$ 7m)
Batch64, AR0 (N=3)	1921 $\pm$ 72	3098 $\pm$ 59	0.60 $\pm$ 0.49	1.00 $\pm$ 0.00 (M=3)	1.00 $\pm$ 0	5,000 (2h 52m $\pm$ 2m)

Supplementary Table 4: Results for Section 2: Can we Circumvent Reward Stagnation? The number of seeds (out of 5) with at least 1 generated molecule satisfying constrained synthesizability is reported with **N**. Metrics (for constrained synthesizable molecules) are reported in two settings: (1) Per-seed: Averaged across seeds (2) Pooled: Pooling all molecules across seeds and de-duplicating. The number of pooled, de-duplicated molecules is denoted by **M**.

**Configuration** **N:** # seeds/5 generating $\geq$ 1 Solved (Enforced)	**Molecule Metrics for Solved (Enforced) Molecules** **Top Values:** Per-seed **Bottom Values:** Pooled (**M** Pooled Molecules)					
	**Docking Score**	**QED**	**Ligand Efficiency**	**IntDiv1**	**#Circles**	**# Scaffolds**
Batch32, AR5 (N=4)	-7.78 $\pm$ 0.42	0.82 $\pm$ 0.02	0.31 $\pm$ 0.02	0.57 $\pm$ 0.08	1.75 $\pm$ 0.96	1018 $\pm$ 772
	-7.71 $\pm$ 0.74 (M=4790)	0.82 $\pm$ 0.08	0.31 $\pm$ 0.04	0.718	5	4058
Batch64, AR10 (N=3)	-8.12 $\pm$ 1.18	0.80 $\pm$ 0.10	0.31 $\pm$ 0.03	0.55 $\pm$ 0.02	2.33 $\pm$ 1.16	800 $\pm$ 488
	-7.64 $\pm$ 0.91 (M=3370)	0.84 $\pm$ 0.09	0.30 $\pm$ 0.04	0.69	5	2395
Batch64, AR5 (N=4)	7.88 $\pm$ 0.31	0.82 $\pm$ 0.01	0.32 $\pm$ 0.01	0.60 $\pm$ 0.02	1.75 $\pm$ 0.96	768 $\pm$ 327
	-7.81 $\pm$ 0.81 (M=4134)	0.82 $\pm$ 0.08	0.32 $\pm$ 0.04	0.72	4	2904
Batch64, AR2 (N=4)	-7.65 $\pm$ 0.58	0.82 $\pm$ 0.07	0.35 $\pm$ 0.04	0.43 $\pm$ 0.29	2.00 $\pm$ 0.82	28 $\pm$ 39
	-7.83 $\pm$ 0.74 (M=167)	0.77 $\pm$ 0.11	0.32 $\pm$ 0.05	0.65	4	108
Batch64, AR0 (N=3)	-8.03 $\pm$ 0.59	0.69 $\pm$ 0.16	0.32 $\pm$ 0.03	0 $\pm$ 0	1 $\pm$ 0	1 $\pm$ 0
	-8.03 $\pm$ 0.48 (M=3)	0.69 $\pm$ 0.13	0.32 $\pm$ 0.02	0.38	1	3

4.4 Supplementary Section 4.4

The results from the previous section identified tentative hyperparameters with a good balance between exploration-exploitation. With this "better" sampling behavior, we wanted to re-visit the reward function formulations as an extensive ablation to affirm that TANGO-TANGO-FMS$_{Block}$ is the best formulation. In this section, we additionally investigate a brute-force approach and a functional groups overlap reward function. **Brute-force** entails only optimizing for retrosynthesis model solvability, that is, whether the retrosynthesis model returns a synthesis route (solved) or not. In this case, no notion of similarity between blocks in the synthesis route and the enforced building blocks is used as a learning signal. Therefore, if any enforced building blocks are indeed used, it would be by chance. The functional groups overlap (**FG**) reward function extracts the functional groups present in the synthesis route nodes and measures the overlap with those in the enforced building blocks. Concretely, for a given synthesis route node, compute: # shared functional groups between the node and an enforced building block, divided by the total number of functional groups in the enforced building block. Do this for every enforced building block and then return the mean. The functional groups themselves are defined by SMARTS patterns encoding common groups adapted from: https://www.daylight.com/dayhtml_tutorials/languages/smarts/smarts_examples.html

Hypotheses:

1. TANGO-TANGO-FMS$_{Block}$ may not be the best reward function formulation now that better exploration-exploitation parameters have been identified. Try all reward function formulations.

2. In the previous section, Batch64, AR5 worked much better than Batch64, AR2, but it might be too exploitative when we consider moving to a smaller set of enforced blocks and/or starting material constraints.

3. More thoroughly study the effect of the sampling behavior by increasing the oracle budget.

Fixed Parameters:

1. **Oracle Budget** = 10,000

2. **Batch Size** = 64

3. **Augmentation Rounds** = 2 or 5

4. **Enforced Building Blocks** = 100

Observations: Tables 5 and 6 show the results with the mean and standard deviation across 5 seeds (0-4 inclusive). We make the following observations:

1. Surprisingly, Brute-force is sometimes successful but is inconsistent, as expected. Notably many molecules are non-solved (no retrosynthesis route found). The MPO is also notably worse than TANGO-FMS$_{Block}$, in agreement with the optimization "by chance" with brute-force learning.

2. FG poorly distinguishes between "goodness" and is essentially unsuccessful, as expected.

3. FMS can distinguish between "goodness" but struggles to guide the optimization, somewhat unexpectedly.

4. TANGO with components of FG are more unsuccessful, in agreement with FG being a poor reward function formulation.

5. TANGO-FMS$_{Block}$ is most consistent reward function with 5/5 successful seeds and amongst the most generated molecules satisfying constrained synthesizability. This is in agreement with initial results.

6. TANGO-FMS$_{Query}$ with the FMS$_{Block}$ implementation (Appendix 4.2) performs worse than TANGO-FMS$_{Block}$. The variance for Solved (Enforced) is higher and much fewer molecules with good docking scores and QED are generated. **For this reason, from here on, the FMS$_{Block}$ implementation is used, as detailed in Appendix 4.2. In the event that there is a false positive when computing FMS$_{Block}$, then FMS$_{Query}$ is used as a fallback.**

7. TANGO-FMS$_{Block}$ with AR5 can outperform TANGO-FMS$_{Block}$ with AR2 but is notably more inconsistent. This affirms our hypothesis that AR5 might be too exploitative. Importantly, the runs with AR5 also have a much longer wall time, again, due to Saturn's

local sampling behavior. Based on these results, AR2 is likely a better balance between exploration-exploitation.

8. Comparing the molecule metrics, TANGO-FMS$_{Block}$ is the most performant reward function, with the best overall docking scores and diversity metrics. The only reward function with better docking scores is FMS$_{Query}$ but only 3/5 seeds were successful and there were only 1546 pooled molecules compared to 8315 for TANGO-FMS$_{Block}$. This further affirms that TANGO-FMS$_{Block}$ is the most consistent reward function.

Taking the results together, we find that TANGO-FMS$_{Block}$ (0.50 FMS$_{Block}$ weighting and 0.50 TanSim weighting) is the most consistent reward function and designate this the default reward function.

Supplementary Table 5: Results for Section 3: Re-visiting Reward Function Formulation for Ablation Studies. The number of seeds (out of 5) with at least 1 generated molecule satisfying constrained synthesizability is reported with **N**. Metrics (for constrained synthesizable molecules) are reported in two settings: (1) Per-seed: Averaged across seeds (2) Pooled: Pooling all molecules across seeds and de-duplicating. The number of pooled, de-duplicated molecules is denoted by **M**.

**Configuration** **N:** # seeds/5 generating $\geq$ 1 Solved (Enforced)	**Synthesizability**			**Solved (Enforced) Molecules** **Reaction Values:** Pooled (**M** Pooled Molecules) **Unique Enforced Block Values:** Per-seed		**Oracle Budget (Wall Time)**
	**Non-solved**	**Solved**	**Solved (Enforced)**	**# Reaction Steps**	**# Unique Enforced Blocks**	
Brute-force (N=3)	5547 ± 1554	4408 ± 1494	2175 ± 1955	2.99 ± 1.54 (M=10876)	1.33 ± 0.47	10,000 (7h 29m ± 2h 11m)
FG (N=4)	2144 ± 263	7822 ± 267	1 ± 1	3.33 ± 1.80 (M=9)	1.75 ± 0.83	10,000 (7h 23m ± 16m)
FMS$_{Block}$ (N=4)	2166 ± 293	7792 ± 304	1 ± 0	2.14 ± 1.46 (M=7)	1.50 ± 0.50	10,000 (7h 35m ± 22m)
FMS$_{Query}$ (N=3)	1967 ± 226	8002 ± 241	315 ± 563	2.67 ± 1.13 (M=1546)	1.33 ± 0.47	10,000 (8h 6m ± 23m)
TANGO-FG (N=5)	1957 ± 203	7987 ± 213	647 ± 949	2.25 ± 1.15 (M=3123)	1.80 ± 0.75	10,000 (8h 29m ± 25m)
TANGO-FMS$_{Block}$ (N=5)	2229 ± 325	7711 ± 333	1744 ± 715	2.38 ± 1.25 (M=8315)	2.20 ± 0.75	10,000 (8h 12m ± 15m)
TANGO-FMS$_{Query}$ (N=5)	2000 ± 211	7945 ± 217	1864 ± 1066	2.24 ± 1.18 (M=8472)	1.20 ± 0.40	10,000 (8h 14m ± 11m)
TANGO-FMS$_{Block}$ (AR5, N=4)	2149 ± 180	7664 ± 174	2522 ± 2061	2.60 ± 1.17 (M=12186)	1.75 ± 0.43	10,000 (12h 36m ± 52m)
TANGO-All (N=4)	2029 ± 219	7919 ± 225	477 ± 576	2.90 ± 1.33 (M=2183)	1.75 ± 0.83	10,000 (8h 1m ± 11m)

Supplementary Table 6: Results for Section 3: Re-visiting Reward Function Formulation for Ablation Studies. The number of seeds (out of 5) with at least 1 generated molecule satisfying constrained synthesizability is reported with **N**. Metrics (for constrained synthesizable molecules) are reported in two settings: (1) Per-seed: Averaged across seeds (2) Pooled: Pooling all molecules across seeds and de-duplicating. The number of pooled, de-duplicated molecules is denoted by **M**.

**Configuration**<br>**N:** # seeds/5 generating<br>$\geq$ 1 Solved (Enforced)	**Molecule Metrics for Solved (Enforced) Molecules**<br>**Top Values:** Per-seed<br>**Bottom Values:** Pooled (**M** Pooled Molecules)					
	**Docking Score**	**QED**	**Ligand Efficiency**	**IntDiv1**	**#Circles**	**# Scaffolds**
Brute-force (N=3)	-7.82 ± 0.39	0.69 ± 0.20	0.31 ± 0.01	0.62 ± 0.08	3.67 ± 4.62	2959 ± 1056
	-7.72 ± 0.95 (M=10876)	0.74 ± 0.18	0.31 ± 0.04	0.794	9	8876
FG (N=4)	-7.60 ± 0.37	0.84 ± 0.06	0.34 ± 0.02	0.34 ± 0.24	2.00 ± 0.82	2 ± 1
	-7.72 ± 0.88 (M=9)	0.84 ± 0.06	0.34 ± 0.03	0.69	5	9
FMS_{Block} (N=4)	-7.69 ± 0.59	0.88 ± 0.02	0.35 ± 0.02	0.28 ± 0.19	1.25 ± 0.50	2 ± 1
	-7.70 ± 1.09 (M=7)	0.88 ± 0.04	0.35 ± 0.03	0.64	3	7
FMS_{Query} (N=3)	-8.68 ± 0.72	0.85 ± 0.02	0.35 ± 0.02	0.53 ± 0.05	2.33 ± 0.58	279 ± 411
	-9.14 ± 0.71 (M=1546)	0.83 ± 0.09	0.34 ± 0.03	0.57	3	788
TANGO-FG (N=5)	-7.45 ± 0.38	0.85 ± 0.06	0.32 ± 0.02	0.49 ± 0.14	1.60 ± 0.55	525 ± 869
	-7.79 ± 1.52 (M=3123)	0.82 ± 0.09	0.30 ± 0.06	0.59	3	2499
TANGO-<br>FMS_{Block} (N=5)	-8.29 ± 0.77	0.83 ± 0.04	0.33 ± 0.00	0.62 ± 0.09	2.60 ± 1.14	1170 ± 587
	-8.12 ± 1.12 (M=8315)	0.83 ± 0.09	0.33 ± 0.04	0.74	6	5290
TANGO-<br>FMS_{Query} (N=5)	-7.80 ± 0.27	0.83 ± 0.03	0.32 ± 0.02	0.55 ± 0.16	1.80 ± 1.30	1268 ± 824
	-7.84 ± 0.98 (M=8472)	0.82 ± 0.09	0.32 ± 0.04	0.72	5	5543
TANGO-<br>FMS_{Block} (AR5, N=4)	-7.40 ± 0.51	0.86 ± 0.04	0.32 ± 0.02	0.54 ± 0.15	2.00 ± 1.41	2513 ± 1751
	-7.65 ± 0.70 (M=12186)	0.85 ± 0.08	0.31 ± 0.03	0.71	3	9619
TANGO-All (N=4)	-8.05 ± 1.07	0.88 ± 0.04	0.34 ± 0.01	0.46 ± 0.31	2.25 ± 1.26	411 ± 467
	-8.74 ± 0.97 (M=2183)	0.86 ± 0.08	0.34 ± 0.04	0.61	3	1426

4.5 Supplementary Section 4.5

Supplementary Table 7: Results for Section 4: Comparing Tanimoto and Tversky similarity. The number of seeds (out of 5) with at least 1 generated molecule satisfying constrained synthesizability is reported with **N**. Metrics (for constrained synthesizable molecules) are reported in two settings: (1) Per-seed: Averaged across seeds (2) Pooled: Pooling all molecules across seeds and de-duplicating. The number of pooled, de-duplicated molecules is denoted by **M**.

**Configuration**<br>**N:** # seeds/5 generating<br>$\geq$ 1 Solved (Enforced)	**Synthesizability**			**Solved (Enforced) Molecules**<br>**Reaction Values:** Pooled (**M** Pooled Molecules)<br>**Unique Enforced Block Values:** Per-seed		**Oracle Budget**<br>**(Wall Time)**
	**Non-solved**	**Solved**	**Solved (Enforced)**	**# Reaction Steps**	**# Unique Enforced Blocks**	
TanSim (N=3)	2275 ± 143	7653 ± 142	1864 ± 1828	2.21 ± 1.16 (M=8731)	1.33 ± 0.47	10,000<br>(9h 11m ± 48m)
Tversky<br>a=0.50, b=0.50 (N=5)	2324 ± 289	7615 ± 293	1595 ± 1088	2.33 ± 1.22 (M=7533)	1.20 ± 0.40	10,000<br>(9h 46m ± 26m)
Tversky<br>a=0.75, b=0.25 (N=4)	2295 ± 209	7635 ± 217	724 ± 869	2.34 ± 1.34 (M=3615)	1.75 ± 0.83	10,000<br>(9h 47m ± 33m)
Tversky<br>a=0.25, b=0.75 (N=4)	2190 ± 190	7740 ± 201	1243 ± 1031	2.35 ± 1.29 (M=5863)	1.50 ± 0.50	10,000<br>(9h 27m ± 20m)
TANGO<br>FMS_{Block}=0.50, TanSim=0.50 (N=5)	2229 ± 325	7711 ± 333	1744 ± 715	2.38 ± 1.25 (M=8315)	2.20 ± 0.75	10,000<br>(8h 12m ± 15m)
TANGO-Tversky<br>FMS_{Block}=0.50, Tversky=0.50<br>(a=0.50, b=0.50) (N=5)	2094 ± 352	7852 ± 358	976 ± 778	2.24 ± 1.24 (M=4793)	2.20 ± 0.98	10,000<br>(10h 3m ± 50m)

Following the results from the previous section, we further re-visit the **Tanimoto similarity (TanSim)** reward function and also investigate **Tversky similarity** as another means of quantifying fingerprint similarity. We continue to use Morgan fingerprints with radius=2 and nBits=1024. For Tversky similarity, we also tune different `a` and `b` values and interrogate performance. Finally, we contrast TANGO-FMS_{Block} using Tanimoto and Tversky similarity. We continue to use the same fixed parameters as the previous section:

Fixed Parameters:

1. **Oracle Budget** = 10,000
2. **Batch Size** = 64
3. **Augmentation Rounds** = 2
4. **Enforced Building Blocks** = 100

Supplementary Table 8: Results for Section 4: Comparing Tanimoto and Tversky similarity. The number of seeds (out of 5) with at least 1 generated molecule satisfying constrained synthesizability is reported with **N**. Metrics (for constrained synthesizable molecules) are reported in two settings: (1) Per-seed: Averaged across seeds (2) Pooled: Pooling all molecules across seeds and de-duplicating. The number of pooled, de-duplicated molecules is denoted by **M**.

**Configuration**<br>**N:** # seeds/5 generating<br>$\geq$ 1 Solved (Enforced)	**Molecule Metrics for Solved (Enforced) Molecules**<br>**Top Values:** Per-seed<br>**Bottom Values:** Pooled (**M** Pooled Molecules)					
	**Docking Score**	**QED**	**Ligand Efficiency**	**IntDiv1**	**#Circles**	**# Scaffolds**
TanSim (N=3)	-7.87 ± 0.47<br>-7.74 ± 0.81 (M=8731)	0.81 ± 0.09<br>0.81 ± 0.08	0.32 ± 0.01<br>0.31 ± 0.04	0.60 ± 0.01<br>0.67	1.33 ± 0.58<br>3	2477 ± 1488<br>6840
Tversky<br>a=0.50, b=0.50 (N=5)	-7.69 ± 0.11<br>-7.68 ± 0.72 (M=7533)	0.84 ± 0.04<br>0.83 ± 0.09	0.32 ± 0.01<br>0.32 ± 0.03	0.58 ± 0.05<br>0.72	1.80 ± 0.84<br>3	1250 ± 944<br>5769
Tversky<br>a=0.75, b=0.25 (N=4)	-8.03 ± 0.75<br>-7.70 ± 0.77 (M=3615)	0.71 ± 0.25<br>0.83 ± 0.09	0.30 ± 0.05<br>0.32 ± 0.03	0.41 ± 0.28<br>0.71	1.50 ± 0.58<br>4	648 ± 732<br>2580
Tversky<br>a=0.25, b=0.75 (N=4)	-7.99 ± 0.51<br>-7.96 ± 0.87 (M=5863)	0.82 ± 0.02<br>0.82 ± 0.09	0.32 ± 0.01<br>0.32 ± 0.03	0.57 ± 0.07<br>0.69	1.50 ± 0.58<br>3	1180 ± 832<br>4333
TANGO-<br>FMS_{Block} (N=5)	-8.29 ± 0.77<br>-8.12 ± 1.12 (M=8315)	0.83 ± 0.04<br>0.83 ± 0.09	0.33 ± 0.00<br>0.33 ± 0.04	0.62 ± 0.09<br>0.74	2.60 ± 1.14<br>6	1170 ± 587<br>5290
TANGO-Tversky<br>FMS_{Block}=0.50, Tversky=0.50<br>(a=0.50, b=0.50) (N=5)	-7.43 ± 0.64<br>-7.77 ± 0.82 (M=4793)	0.84 ± 0.03<br>0.84 ± 0.09	0.32 ± 0.00<br>0.32 ± 0.03	0.53 ± 0.18<br>0.72	3.00 ± 1.87<br>7	656 ± 570<br>3196

Observations: Tables 7 and 8 show the results with the mean and standard deviation across 5 seeds (0-4 inclusive). We make the following observations:

1. Amongst the Tversky parameter combinations, equal $\texttt{a} = 0.50$ and $\texttt{b} = 0.50$ weighting is generally the most performant. 5/5 seeds were successful and the highest number of pooled molecules satisfying constrained synthesizability (7,533). While $\texttt{a} = 0.25$ and $\texttt{b} = 0.75$ yields better docking scores, only 4/5 seeds were successful and there are less pooled molecules (5,863).

2. Comparing the best Tversky parameter combination, equal $\texttt{a} = 0.50$ and $\texttt{b} = 0.50$ to TanSim, the synthesizability and molecule metrics are similar, making it difficult to discern any

differences.

3. Therefore, we compare TANGO-FMS using TanSim and Tversky equal $\mathtt{a} = 0.50$ and $\mathtt{b} = 0.50$ similarity. Here, differences are clearer and TANGO-FMS with TanSim notably results in more molecules satisfying constrained synthesizability (Solved (Enforced) metric). Moreover, the docking scores and diversity metrics are also better.

4. Lastly, comparing TANGO-FMS$_{Block}$ using TanSim with TanSim alone and Tversky equal $\mathtt{a} = 0.50$ and $\mathtt{b} = 0.50$, TANGO-FMS$_{Block}$ using TanSim is the best reward function across all synthesizability (N successful seeds) and molecule metrics.

Taking the results from the previous section and this section, we continue to fix TANGO-FMS$_{Block}$ using TanSim as the default reward function. **From here on, we refer to TANGO-FMS$_{Block}$ as TANGO.**

4.6 Supplementary Section 4.6

The results from the two previous sections identified hyperparameters with good balance between exploration-exploitation. Thus far, all TANGO formulations weight each component equally. The next question we asked was whether certain components were more important?

Hypotheses:

1. FMS_{Block} should be more informative than TanSim to inform chemical reactivity. Test the effect of components weighting.

Fixed Parameters:

1. **Reward Function** = TANGO (0.50 FMS_{Block} and 0.50 TanSim weighting)
2. **Oracle Budget** = 10,000
3. **Batch Size** = 64
4. **Augmentation Rounds** = 2
5. **Enforced Building Blocks** = 100

Observations: Tables 9 and 10 show the results with the mean and standard deviation across 5 seeds (0-4 inclusive). "High" indicates 0.75 weighting while the other component is 0.25. TANGO has equal weighting (0.5 FMS_{Block}, 0.5 TanSim). We make the following observations:

1. TANGO with equal weighting performs similarly to TANGO-High-FMS, with the former potentially able to generate molecules with better docking scores.
2. TANGO-High-TanSim generates more solved molecules but docking scores are worse. These suggests suggest that MPO is better with TANGO (equal weighting) and is the reward function we use from here on.

Supplementary Table 9: Results for Section 5: In TANGO-FMS, is either FMS_{Block} or Tanimoto Similarity more Important? The number of seeds (out of 5) with at least 1 generated molecule satisfying constrained synthesizability is reported with **N**. Metrics (for constrained synthesizable molecules) are reported in two settings: (1) Per-seed: Averaged across seeds (2) Pooled: Pooling all molecules across seeds and de-duplicating. The number of pooled, de-duplicated molecules is denoted by **M**.

**Configuration** **N:** # seeds/5 generating $\geq$ 1 Solved (Enforced)	**Synthesizability**			**Solved (Enforced) Molecules** **Reaction Values:** Pooled (**M** Pooled Molecules) **Unique Enforced Block Values:** Per-seed		**Oracle Budget (Wall Time)**
	**Non-solved**	**Solved**	**Solved (Enforced)**	**# Reaction Steps**	**# Unique Enforced Blocks**	
TANGO (0.50 FMS_{Block}, 0.50 TanSim) (N=5)	2229 ± 325	7711 ± 333	1744 ± 715	2.35 ± 1.24 (M=8719)	2.20 ± 0.75	10,000 (8h 12m ± 15m)
TANGO (0.75 FMS_{Block}, 0.25 TanSim) (N=5)	1960 ± 165	7980 ± 175	1725 ± 748	2.45 ± 1.24 (M=7698)	1.60 ± 0.49	10,000 (8h 36m ± 16m)
TANGO (0.25 FMS_{Block}, 0.75 TanSim) (N=5)	2464 ± 437	7476 ± 440	2738 ± 1039	2.35 ± 1.30 (M=12936)	1.80 ± 0.40	10,000 (8h 51m ± 26m)

Supplementary Table 10: Results for Section 5: In TANGO-FMS, is either FMS_{Block} or Tanimoto Similarity more Important? The number of seeds (out of 5) with at least 1 generated molecule satisfying constrained synthesizability is reported with **N**. Metrics (for constrained synthesizable molecules) are reported in two settings: (1) Per-seed: Averaged across seeds (2) Pooled: Pooling all molecules across seeds and de-duplicating. The number of pooled, de-duplicated molecules is denoted by **M**.

**Configuration** **N:** # seeds/5 generating $\geq$ 1 Solved (Enforced)	**Molecule Metrics for Solved (Enforced) Molecules** **Top Values:** Per-seed **Bottom Values:** Pooled (**M** Pooled Molecules)					
	**Docking Score**	**QED**	**Ligand Efficiency**	**IntDiv1**	**#Circles**	**# Scaffolds**
TANGO (0.50 FMS_{Block}, 0.50 TanSim) (N=5/5)	-8.29 ± 0.77<br>-8.12 ± 1.12 (M=8315)	0.83 ± 0.04<br>0.83 ± 0.09	0.33 ± 0.00<br>0.33 ± 0.04	0.62 ± 0.09<br>0.74	2.60 ± 1.14<br>6	1170 ± 587<br>5290
TANGO (0.75 FMS_{Block}, 0.25 TanSim) (N=5/5)	-7.81 ± 0.23<br>-7.77 ± 0.80 (M=7698)	0.86 ± 0.01<br>0.86 ± 0.08	0.32 ± 0.01<br>0.32 ± 0.03	0.61 ± 0.05<br>0.66	1.80 ± 1.10<br>2	1219 ± 546<br>5218
TANGO (0.25 FMS_{Block}, 0.75 TanSim) (N=5/5)	-7.85 ± 0.47<br>-7.78 ± 0.83 (M=12936)	0.83 ± 0.03<br>0.82 ± 0.10	0.32 ± 0.01<br>0.32 ± 0.04	0.61 ± 0.03<br>0.74	1.80 ± 0.84<br>6	2120 ± 1070<br>9799

4.7 Supplementary Section 4.7

With optimal hyperparameters identified, we expand to robustness studies and run every experiment across 10 seeds (0-9 inclusive) and investigate enforcing a smaller set of building blocks. We also probe whether the starting material constraint is also learnable within the oracle budget. Finally, we also perform a set of experiments without the QED objective.

Fixed Parameters:

1. **Reward Function** = TANGO (0.50 FMS_{Block} and 0.50 TanSim weighting)
2. **Oracle Budget** = 10,000
3. **Batch Size** = 64
4. **Augmentation Rounds** = 2
5. **Enforced Building Blocks** = 100

Observations: Tables 11 and 12 show the results with the mean and standard deviation across 10 seeds (0-9 inclusive). We make the following observations:

1. All constraints are learnable.
2. When not enforcing QED, the model generates many more molecules with "good" docking scores, and expectedly, at the expense of QED. This affirms that the MPO is tunable, allowing tailored design of molecules that are also constrained by synthesis. We further observe that without the QED constraint, generated molecules are larger in order to exploit that docking scores are correlated with size (see the Ligand Efficiency metric).
3. Also as expected, when not enforcing QED, the average reaction steps is longer, since QED constrains molecular weight.
4. In general, all constraints lead to generated molecules satisfying the objective function, while maintaining diversity. Concretely, when enforcing QED, we observe that QED remains high. Conversely, when omitting QED, QED is low, and docking scores are better. This shows that MPO can be tuned and the model is learning to optimize the objective function.

5. Elaborating on diversity, the IntDiv1, #Circles, and # Scaffolds metrics demonstrate that enforcing the presence of building blocks still leads to molecules that are dissimilar.

Supplementary Table 11: Results for Section 6: Investigating Robustness. "SM" denotes starting material constrained. The number of seeds (out of 10) with at least 1 generated molecule satisfying constrained synthesizability is reported with **N**. Metrics (for constrained synthesizable molecules) are reported in two settings: (1) Per-seed: Averaged across seeds (2) Pooled: Pooling all molecules across seeds and de-duplicating. The number of pooled, de-duplicated molecules is denoted by **M**.

**Configuration**<br>**N:** # seeds/10 generating<br>$\geq$ 1 Solved (Enforced)	**Synthesizability**			**Solved (Enforced) Molecules**<br>**Reaction Values:** Pooled (**M** Pooled Molecules)<br>**Unique Enforced Block Values:** Per-seed		**Oracle Budget**<br>**(Wall Time)**
	**Non-solved**	**Solved**	**Solved (Enforced)**	**# Reaction Steps**	**# Unique Enforced Blocks**	
100 Blocks (N=10)	2288 ± 305	7649 ± 309	2112 ± 1170	2.42 ± 1.27 (M=19221)	1.9 ± 0.70	10,000<br>(8h 31m ± 40m)
100 Blocks (no QED) (N=10)	1848 ± 158	8146 ± 165	3723 ± 682	3.29 ± 1.19 (M=34997)	1.90 ± 0.75	10,000<br>(7h 27m ± 13m)
100 Blocks (SM) (N=10)	1879 ± 186	8067 ± 180	1525 ± 503	1.59 ± 1.00 (M=11299)	1.90 ± 0.70	10,000<br>(8h 33m ± 30m)
100 Blocks (SM, no QED) (N=10)	1733 ± 172	8238 ± 172	1189 ± 963	2.40 ± 1.18 (M=11094)	1.60 ± 0.66	10,000<br>(8h 3m ± 33m)
10 Blocks (N=6)	2424 ± 288	7498 ± 292	984 ± 1182	2.71 ± 1.22 (M=8562)	1.00 ± 0	10,000<br>(8h 29m ± 30m)
10 Blocks (no QED) (N=9)	1967 ± 211	7999 ± 221	2640 ± 1067	3.23 ± 1.25 (M=24780)	1.22 ± 0.42	10,000<br>(7h 51m ± 33m)
10 Blocks (SM) (N=9)	2225 ± 181	7703 ± 192	1004 ± 926	2.59 ± 1.07 (M=8311)	1.00 ± 0	10,000<br>(8h 39m ± 24m)
10 Blocks (SM, no QED) (N=10)	1835 ± 169	8123 ± 176	2526 ± 597	2.86 ± 1.15 (M=22830)	1.30 ± 0.64	10,000<br>(8h 7m ± 34m)

Supplementary Table 12: Results for Section 6: Investigating Robustness. "SM" denotes starting material constrained. The number of seeds (out of 10) with at least 1 generated molecule satisfying constrained synthesizability is reported with **N**. Metrics (for constrained synthesizable molecules) are reported in two settings: (1) Per-seed: Averaged across seeds (2) Pooled: Pooling all molecules across seeds and de-duplicating. The number of pooled, de-duplicated molecules is denoted by **M**.

**Configuration** **N:** # seeds/10 generating $\geq$ 1 Solved (Enforced)	**Molecule Metrics for Solved (Enforced) Molecules** **Top Values:** Per-seed **Bottom Values:** Pooled (**M** Pooled Molecules)					
	**Docking Score**	**QED**	**Ligand Efficiency**	**IntDiv1**	**#Circles**	**# Scaffolds**
100 Blocks (N=10)	-8.05 $\pm$ 0.78	0.82 $\pm$ 0.05	0.34 $\pm$ 0.04	0.56 $\pm$ 0.21	2.60 $\pm$ 1.51	1452 $\pm$ 1029
	-8.08 $\pm$ 0.98 (M=19221)	0.82 $\pm$ 0.09	0.32 $\pm$ 0.04	0.76	7	12672
100 Blocks (no QED) (N=10)	-9.17 $\pm$ 0.34	0.29 $\pm$ 0.02	0.26 $\pm$ 0.01	0.60 $\pm$ 0.03	2.30 $\pm$ 1.49	2285 $\pm$ 696
	-9.20 $\pm$ 1.17 (M=34997)	0.28 $\pm$ 0.14	0.26 $\pm$ 0.04	0.70	12	19661
100 Blocks (SM) (N=10)	-7.66 $\pm$ 0.45	0.85 $\pm$ 0.03	0.33 $\pm$ 0.01	0.622 $\pm$ 0.04	2.40 $\pm$ 1.17	1101 $\pm$ 477
	-7.70 $\pm$ 0.92 (M=11299)	0.84 $\pm$ 0.08	0.32 $\pm$ 0.04	0.74	5	7511
100 Blocks (SM, no QED) (N=10)	-8.50 $\pm$ 0.39	0.44 $\pm$ 0.08	0.29 $\pm$ 0.01	0.54 $\pm$ 0.19	1.90 $\pm$ 0.74	836 $\pm$ 764
	-8.47 $\pm$ 1.00 (M=11094)	0.45 $\pm$ 0.21	0.28 $\pm$ 0.05	0.75	8	7471
10 Blocks (N=6)	-8.91 $\pm$ 0.36	0.82 $\pm$ 0.02	0.33 $\pm$ 0.01	0.57 $\pm$ 0.08	2.00 $\pm$ 0.89	909 $\pm$ 743
	-8.70 $\pm$ 1.27 (M=8562)	0.83 $\pm$ 0.10	0.33 $\pm$ 0.05	0.65	4	4590
10 Blocks (no QED) (N=9)	-8.96 $\pm$ 0.42	0.40 $\pm$ 0.05	0.26 $\pm$ 0.01	0.58 $\pm$ 0.07	1.78 $\pm$ 0.83	1764 $\pm$ 489
	-8.96 $\pm$ 1.00 (M=24780)	0.38 $\pm$ 0.16	0.26 $\pm$ 0.05	0.71	5	14251
10 Blocks (SM) (N=9)	-8.86 $\pm$ 0.49	0.84 $\pm$ 0.04	0.34 $\pm$ 0.02	0.49 $\pm$ 0.16	1.44 $\pm$ 0.53	663 $\pm$ 539
	-8.73 $\pm$ 1.33 (M=8311)	0.83 $\pm$ 0.09	0.33 $\pm$ 0.05	0.641	5	4726
10 Blocks (SM, no QED) (N=10)	-8.92 $\pm$ 0.43	0.41 $\pm$ 0.06	0.27 $\pm$ 0.02	0.57 $\pm$ 0.06	1.80 $\pm$ 0.79	1526 $\pm$ 550
	-8.86 $\pm$ 0.97 (M=22830)	0.40 $\pm$ 0.18	0.26 $\pm$ 0.05	0.71	7	12904

4.8 Supplementary Section 4.8

From the previous set of experiments, we noticed that the generative model was always incorporating the same 3 enforced building blocks. One in particular was especially common, such that most runs using the set of 10 enforced blocks, use it. We questioned whether TANGO's success was due to luck in having "suitable" building blocks. Therefore, we perform further ablation experiments that purge these 3 building blocks. Similar to the previous set of experiments, we run every configuration here across 10 seeds (0-9 inclusive).

Fixed Parameters:

1. **Oracle Budget** = 10,000
2. **Batch Size** = 64
3. **Augmentation Rounds** = 2
4. **Reward Function** = TANGO-FMS (equal weighting)
5. **Purged Enforced Building Blocks** = 97 (Purged the 3 common enforced building blocks from the set of 100)

Observations: Tables 13 and 14 show the results with the mean and standard deviation across 10 seeds (0-9 inclusive). We make the following observations:

1. Other building blocks can be enforced.
2. The runs become less consistent (less successful seeds out of 10). Runs without QED are more consistently successful, reinforcing that the success of constrained synthesizability deeply depends on the difficult of the MPO objective as omitting QED results in an easier objective function.
3. The diversity of the molecules is lower in this purged blocks setting with notably higher # Reaction Steps and the # Unique Enforced Blocks is low. This leads to follow-up question, whether there is now another particular building block that is consistently enforced. We explore this in the next sub-section.

Supplementary Table 13: Results for Section 7: Lucky Building Blocks? "SM" denotes starting material constrained. The number of seeds (out of 10) with at least 1 generated molecule satisfying constrained synthesizability is reported with **N**. Metrics (for constrained synthesizable molecules) are reported in two settings: (1) Per-seed: Averaged across seeds (2) Pooled: Pooling all molecules across seeds and de-duplicating. The number of pooled, de-duplicated molecules is denoted by **M**.

**Configuration** **N:** # seeds/10 generating $\geq$ 1 Solved (Enforced)	**Synthesizability**			**Solved (Enforced) Molecules** **Reaction Values:** Pooled (**M** Pooled Molecules) **Unique Enforced Block Values:** Per-seed		**Oracle Budget (Wall Time)**
	**Non-solved**	**Solved**	**Solved (Enforced)**	**# Reaction Steps**	**# Unique Enforced Blocks**	
100 Blocks Purged (N=4)	2322 ± 233	7605 ± 241	77 ± 229	5.98 ± 1.16 (M=768)	1 ± 0	10,000 (8h 54m ± 20m)
100 Blocks Purged (no QED) (N=9)	1794 ± 193	8196 ± 189	1553 ± 1211	3.36 ± 1.19 (M=15315)	1.44 ± 0.50	10,000 (7h 15m ± 12m)
100 Blocks Purged (SM) (N=5)	2179 ± 298	7762 ± 310	166 ± 333	4.13 ± 2.29 (M=1658)	1.40 ± 0.80	10,000 (8h 41m ± 28m)
100 Blocks Purged (SM, no QED) (N=8)	1688 ± 239	8286 ± 243	1456 ± 1112	3.33 ± 1.28 (M=14120)	1.62 ± 0.70	10,000 (7h 40m ± 25m)

Supplementary Table 14: Results for Section 7: Lucky Building Blocks? "SM" denotes starting material constrained. The number of seeds (out of 10) with at least 1 generated molecule satisfying constrained synthesizability is reported with **N**. Metrics (for constrained synthesizable molecules) are reported in two settings: (1) Per-seed: Averaged across seeds (2) Pooled: Pooling all molecules across seeds and de-duplicating. The number of pooled, de-duplicated molecules is denoted by **M**.

**Configuration** **N:** # seeds/10 generating $\geq$ 1 Solved (Enforced)	**Molecule Metrics for Solved (Enforced) Molecules** **Top Values:** Per-seed **Bottom Values:** Pooled (**M** Pooled Molecules)					
	**Docking Score**	**QED**	**Ligand Efficiency**	**IntDiv1**	**#Circles**	**# Scaffolds**
100 Blocks Purged (N=4)	-8.04 ± 0.76	0.84 ± 0.06	0.33 ± 0.04	0.26 ± 0.22	1.25 ± 0.50	171 ± 338
	-8.47 ± 0.69 (M=8562)	0.89 ± 0.05	0.33 ± 0.04	0.53	3	682
100 Blocks Purged (no QED) (N=9)	-8.79 ± 0.53	0.36 ± 0.11	0.26 ± 0.03	0.56 ± 0.04	1.44 ± 0.73	971 ± 704
	-8.99 ± 1.14 (M=15315)	0.32 ± 0.16	0.26 ± 0.04	0.72	5	8264
100 Blocks Purged (SM) (N=5)	-7.66 ± 0.59	0.89 ± 0.04	0.34 ± 0.02	0.28 ± 0.31	1.40 ± 0.55	291 ± 404
	-8.05 ± 0.75 (M=1658)	0.86 ± 0.07	0.33 ± 0.04	0.71	6	1455
100 Blocks Purged (SM, no QED) (N=8)	-8.64 ± 0.42	0.39 ± 0.06	0.26 ± 0.02	0.57 ± 0.04	1.50 ± 0.76	1212 ± 666
	-8.71 ± 0.95 (M=14120)	0.37 ± 0.15	0.26 ± 0.05	0.71	7	9196

4.9 Supplementary Section 4.9

In Tables 13 and 14, the results show that the generated molecules converge to a small number of unique enforced building blocks. This suggests the possibility of another "lucky block". We show that while "100 Blocks Purged" (with QED constraint) does converge mostly to another specific building block, removing the QED constraint results in different and more blocks (with some overlap compared to the QED constraint experiment). This supports the hypothesis that certain blocks can be more favorable for the objective function. To show that there are many unique blocks enforced, we visualize the synthesis route of the best docking score molecule for each unique enforced block across the "100 Blocks Purged (with QED)" and "100 Blocks Purged (without QED)" experiments in Figures 6 and 7, respectively. Importantly, we also annotate the frequency of the specific enforced building block compared to the full pooled set of molecules, that is, out of all generated molecules across the successful seeds, how many times was a particular enforced block used? Note that if a particular generated molecule contains > 1 enforced building block, all are counted in the frequencies.

4.10 Supplementary Section 4.10

We next push our framework further by curating 5 building blocks (Fig. 8) that are dissimilar and/or can be involved in different reaction chemistries. Our objective was to investigate whether the model can learn to incorporate such a small set of blocks and whether other chemical reactions can be enforced.

Fixed Parameters:

1. **Oracle Budget** = 10,000 or 15,000
2. **Batch Size** = 64
3. **Augmentation Rounds** = 2
4. **Reward Function** = TANGO-FMS (equal weighting)
5. **Enforced Building Blocks** = 5 (dissimilar to the ones used thus far)

Observations: Tables 15 and Tables 16 show the results with the mean and standard deviation across 10 seeds (0-9 inclusive). We make the following observations:

1. The task is challenging under the 10,000 oracle budget when QED is also optimized for.
2. Without optimizing for QED and increasing the oracle budget to 15,000 results in some successes (2/10 seeds).
3. The two successful replicates both enforced only the Suzuki block (Boron containing) which is circled in Fig. 8.
4. The results here show that learning to enforce such a small set of building blocks is possible. In practice, one could further increase the oracle budget which we did not explore due to time limits on the cluster we used. The two successful replicates (with a 15,000 oracle budget) took about 12.5 hours which we believe is still very reasonable.

Supplementary Table 15: Results for Section 8: 5 Enforced Blocks. The number of seeds (out of 10) with at least 1 generated molecule satisfying constrained synthesizability is reported with **N**. Metrics (for constrained synthesizable molecules) are reported in two settings: (1) Per-seed: Averaged across seeds (2) Pooled: Pooling all molecules across seeds and de-duplicating. The number of pooled, de-duplicated molecules is denoted by **M**.

**Configuration**<br>**N:** # seeds/10 generating<br>$\geq$ 1 Solved (Enforced)	**Synthesizability**			**Solved (Enforced) Molecules**<br>**Reaction Values:** Pooled (**M** Pooled Molecules)<br>**Unique Enforced Block Values:** Per-seed		**Oracle Budget**<br>**(Wall Time)**
	**Non-solved**	**Solved**	**Solved (Enforced)**	**# Reaction Steps**	**# Unique Enforced Blocks**	
5 Blocks (N=0)	2638 $\pm$ 184	7282 $\pm$ 189	0 $\pm$ 0	N/A	N/A	10,000<br>(9h 24m $\pm$ 25m)
5 Blocks (no QED)<br>15k Budget (N=2)	3333 $\pm$ 437	11598 $\pm$ 430	972 $\pm$ 2112	3.79 $\pm$ 0.83 (M=9723)	1.00 $\pm$ 0	15,000<br>(12h 33m $\pm$ 34m)

Supplementary Table 16: Results for Section 8: 5 Enforced Blocks. The number of seeds (out of 10) with at least 1 generated molecule satisfying constrained synthesizability is reported with **N**. Metrics (for constrained synthesizable molecules) are reported in two settings: (1) Per-seed: Averaged across seeds (2) Pooled: Pooling all molecules across seeds and de-duplicating. The number of pooled, de-duplicated molecules is denoted by **M**.

**Configuration**<br>**N:** # seeds/10 generating<br>$\geq$ 1 Solved (Enforced)	**Molecule Metrics for Solved (Enforced) Molecules**<br>**Top Values:** Per-seed<br>**Bottom Values:** Pooled (**M** Pooled Molecules)					
	**Docking Score**	**QED**	**Ligand Efficiency**	**IntDiv1**	**#Circles**	**# Scaffolds**
5 Blocks (N=0)	N/A<br>N/A (M=0)	N/A<br>N/A	N/A<br>N/A	N/A<br>N/A	N/A<br>N/A	N/A<br>N/A
5 Blocks (no QED)<br>15k Budget (N=2)	-11.10 $\pm$ 0.46<br>-10.98 $\pm$ 1.52 (M=9723)	0.30 $\pm$ 0.04<br>0.31 $\pm$ 0.10	0.27 $\pm$ 0.00<br>0.27 $\pm$ 0.04	0.51 $\pm$ 0.09<br>0.57	1 $\pm$ 0<br>1	3857 $\pm$ 1792<br>7714

4.11 Supplementary Section 4.11

Often, divergent synthesis [18] is desirable, whereby intermediates (usually non-commercially available) are enforced in the synthesis path. This can be used for late-stage functionalization [19] which is particularly relevant in drug discovery to explore SAR. In this section, we select intermediate non-commercial blocks from solved paths. We note that this is artificial in the sense that these selected intermediates were taken from solved routes, and are likely "favorable". However, we were interested in whether a model can learn from scratch to enforce relatively large building blocks. For this reason, we curated 10 selected intermediates and investigate the ability of TANGO to learn divergent synthesis constraints.

Fixed Parameters:

1. **Oracle Budget** = 10,000 or 15,000
2. **Batch Size** = 64
3. **Augmentation Rounds** = 2
4. **Reward Function** = TANGO-FMS (equal weighting)
5. **Divergent Enforced Building Blocks** = 10 (Curated from successful runs)

Observations: Tables 17 and 18 show the results with the mean and standard deviation across 10 seeds (0-9 inclusive). We make the following observations:

1. Divergent blocks can be enforced but the runs are less consistently successful than with the original sets of enforced building blocks, under a 10,000 oracle budget.
2. The runs do not necessarily take longer which means that in practical applications, one could increase the oracle budget. We believe that the wall times of all our experiments (7-9 hours) are reasonable and that much longer is tolerable in real-world applications ($\leq$ 24h and even $>$ 24h if the model can truly solve the MPO task).
3. Increasing the oracle budget to 15,000 results in more successful seeds. Therefore, simply using more compute (within reason) is a straightforward solution.

Supplementary Table 17: Results for Section 9: Divergent Synthesis. The number of seeds (out of 10) with at least 1 generated molecule satisfying constrained synthesizability is reported with **N**. Metrics (for constrained synthesizable molecules) are reported in two settings: (1) Per-seed: Averaged across seeds (2) Pooled: Pooling all molecules across seeds and de-duplicating. The number of pooled, de-duplicated molecules is denoted by **M**.

**Configuration**<br>**N:** # seeds/10 generating<br>$\geq$ 1 Solved (Enforced)	**Synthesizability**			**Solved (Enforced) Molecules**<br>**Reaction Values:** Pooled (**M** Pooled Molecules)<br>**Unique Enforced Block Values:** Per-seed		**Oracle Budget**<br>**(Wall Time)**
	**Non-solved**	**Solved**	**Solved (Enforced)**	**# Reaction Steps**	**# Unique Enforced Blocks**	
Divergent Blocks (N=4)	2166 $\pm$ 203	7770 $\pm$ 214	651 $\pm$ 1238	3.69 $\pm$ 1.08 (M=6372)	1.75 $\pm$ 0.83	10,000<br>(8h 52m $\pm$ 42m)
Divergent Blocks<br>15k Budget (N=5)	3715 $\pm$ 633	11150 $\pm$ 636	1519 $\pm$ 2321	3.63 $\pm$ 1.10 (M=14503)	1.80 $\pm$ 1.17	15,000<br>(15h 54m $\pm$ 1h 30m)
Divergent Blocks<br>(no QED) (N=3)	1937 $\pm$ 210	8043 $\pm$ 217	540 $\pm$ 1259	4.17 $\pm$ 1.35 (M=5260)	1.00 $\pm$ 0	10,000<br>(7h 39m $\pm$ 23m)
Divergent Blocks<br>(no QED, 15k Budget) (N=4)	2865 $\pm$ 523	12078 $\pm$ 531	839 $\pm$ 1972	4.30 $\pm$ 1.36 (M=8393)	2.00 $\pm$ 0.71	15,000<br>(12h 41m $\pm$ 25m)

Supplementary Table 18: Results for Section 9: Divergent Synthesis. The number of seeds (out of 10) with at least 1 generated molecule satisfying constrained synthesizability is reported with **N**. Metrics (for constrained synthesizable molecules) are reported in two settings: (1) Per-seed: Averaged across seeds (2) Pooled: Pooling all molecules across seeds and de-duplicating. The number of pooled, de-duplicated molecules is denoted by **M**.

**Configuration**<br>**N:** # seeds/10 generating<br>$\geq$ 1 Solved (Enforced)	**Molecule Metrics for Solved (Enforced) Molecules**<br>**Top Values:** Per-seed<br>**Bottom Values:** Pooled (**M** Pooled Molecules)					
	**Docking Score**	**QED**	**Ligand Efficiency**	**IntDiv1**	**#Circles**	**# Scaffolds**
Divergent Blocks (N=4)	-8.77 $\pm$ 0.55<br>-8.48 $\pm$ 0.93 (M=6372)	0.85 $\pm$ 0.03<br>0.86 $\pm$ 0.07	0.33 $\pm$ 0.01<br>0.32 $\pm$ 0.04	0.44 $\pm$ 0.22<br>0.65	1 $\pm$ 0<br>3	1233 $\pm$ 1253<br>4780
Divergent Blocks<br>15k Budget (N=5)	-8.48 $\pm$ 0.51<br>-8.53 $\pm$ 0.90 (M=14503)	0.84 $\pm$ 0.03<br>0.86 $\pm$ 0.08	0.33 $\pm$ 0.01<br>0.33 $\pm$ 0.04	0.58 $\pm$ 0.06<br>0.68	1 $\pm$ 0<br>4	2441 $\pm$ 2030<br>11557
Divergent Blocks<br>(no QED) (N=3)	-9.63 $\pm$ 0.77<br>-9.28 $\pm$ 0.95 (M=5260)	0.43 $\pm$ 0.04<br>0.43 $\pm$ 0.21	0.28 $\pm$ 0.02<br>0.29 $\pm$ 0.05	0.39 $\pm$ 0.34<br>0.63	1 $\pm$ 0<br>3	1213 $\pm$ 1490<br>3531
Divergent Blocks<br>(no QED, 15k Budget) (N=4)	-9.15 $\pm$ 0.45<br>-9.31 $\pm$ 0.95 (M=8562)	0.44 $\pm$ 0.10<br>0.41 $\pm$ 0.19	0.26 $\pm$ 0.03<br>0.27 $\pm$ 0.05	0.51 $\pm$ 0.10<br>0.70	1.25 $\pm$ 0.50<br>5	1633 $\pm$ 2301<br>6533

5 Supplementary Section 5

5.1 Supplementary Section 5.1

In this section, we show examples of synthetic routes from the MEGAN [6] retrosynthesis model with enforced building blocks. The synthesis graph images were taken as is from Syntheseus' [5] output. Figures 9, 10, 11, 12, and 13 show routes from the 100, 100 with "lucky" blocks purged, 10, 5, and divergent blocks experiments. Moreover, the routes are for molecules with docking score $<$ -10.5 since these are the most optimal. In addition, for the 5 Enforced Blocks, the routes shown were from the runs without QED (Fig. 12). This is because these were the only seeds that were successful under the oracle budget. **The enforced block is boxed.** We also try to show some diversity in the route lengths to highlight that path length was not explicitly optimized for. QED implicitly encourages shorter paths due to constraining the molecular weight, but even so, longer synthetic routes can still be observed (for example in Fig. 10). Future work could also reward shorter paths.

5.2 Supplementary Section 5.2

We further analyze the synthesis routes for the generated molecules satisfying constrained synthesizability for the following experiments: "100 Blocks", "10 Blocks", "100 Blocks Purged", and "Divergent Blocks" all with the TANGO reward function (FMS_{Block}=0.50, TanSim=0.50 weighting). Fig. 14 shows the top-9 most common reaction classes across both the runs with QED enforced and without. The reaction classes were labeled using NameRXN [20]. We make several observations: (1) "N-acylation to amide" is by far the most common reaction. (2) Deprotection reactions are common. (3) "Nitro to amine reduction" is almost exclusively used in the non-QED runs. We make general observations only, as we believe the types of reactions used deeply depends on the nature of the enforced blocks, as they dictate what reactions would be compatible. If a specific building block is commonly enforced, then that biases the reactions used. Finally, TANGO does not explicitly control for reaction types.

5.3 Supplementary Section 5.3

In rare cases, some generated molecules possess synthesis routes with 2 enforced building blocks (we observed a handful of cases with > 2), even though this was not explicitly rewarded. For each individual experiment in the paper, we report the occurrence of this relative to the total number of molecules with an enforced block (Table 19). Fig. 15 shows an example route from the "100 Blocks" experiment with 2 enforced building blocks (highlighted in yellow and beach).

Supplementary Table 19: The number of generated molecules with more than 1 enforced building block. All metrics are reported for the pooled molecules across seeds and de-duplicating.

[a] 81/34997 3 blocks and 1/34997 with 4 blocks.

[b] 56/11094 3 blocks and 4/11094 with 4 blocks.

[c] 5/15315 3 blocks.

Configuration	Double Blocks Occurrences
100 Blocks	190/19221
100 Blocks (no QED)	3172/34997[a]
100 Blocks (SM)	115/11299
100 Blocks (SM) (no QED)	1264/11094[b]
10 Blocks	39/8562
10 Blocks (no QED)	460/24780
10 Blocks (SM)	33/8311
10 Blocks (SM) (no QED)	568/22830
100 Blocks Purged	0/768
100 Blocks Purged (no QED)	948/15315[c]
100 Blocks Purged (SM)	5/1658
100 Blocks Purged (SM) (no QED)	429/14120
Divergent Blocks	26/6372
Divergent Blocks (no QED)	124/5260
COX2 Blocks	359/22407

6 Supplementary Section 6

Supplementary Figure 1: TANGO Example Computation

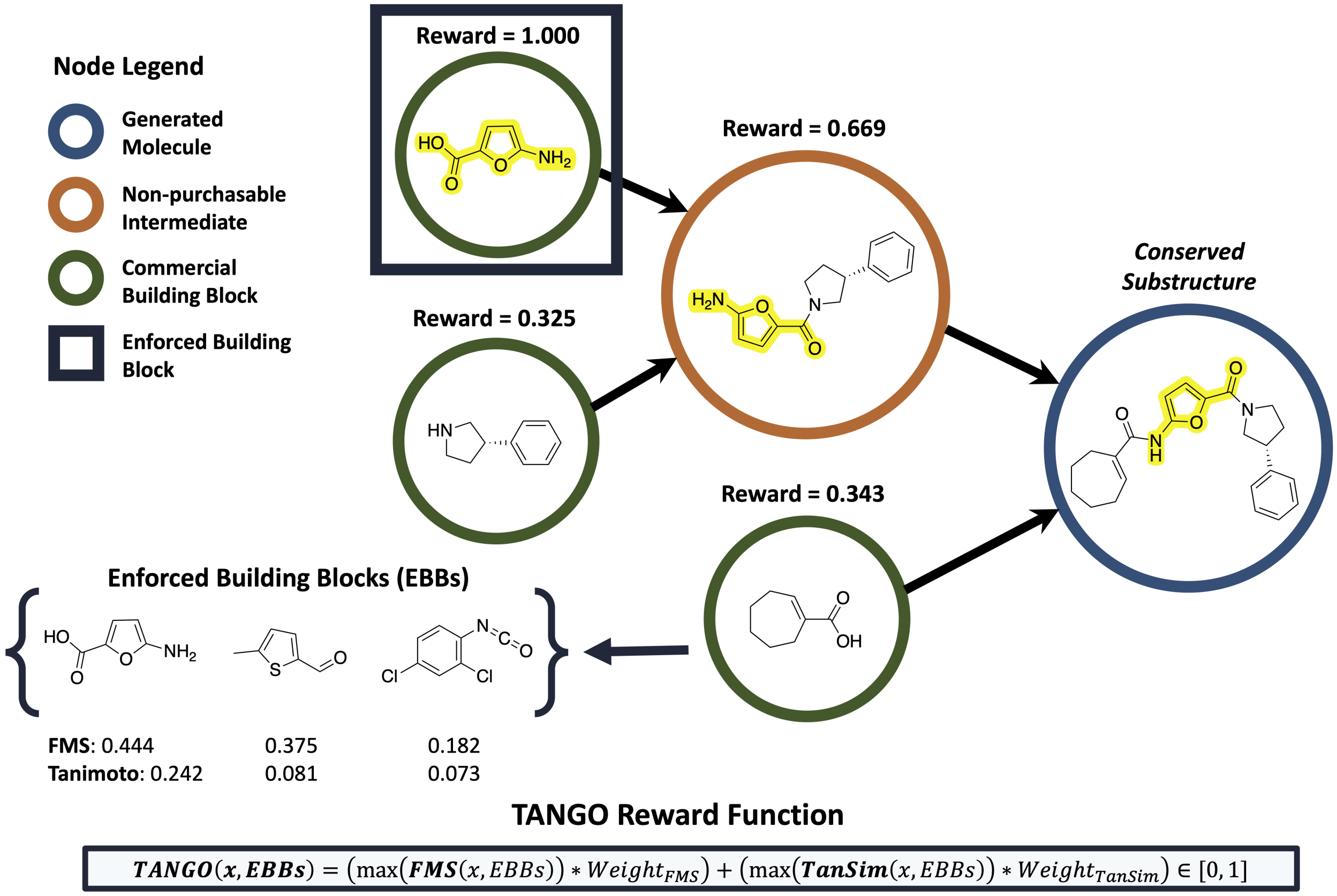

Supplementary Figure 1: TANGO reward function: the maximum similarity between every non-root node (generated molecule) molecule and the set of enforced building blocks. Every synthesizable generated molecule returns a non-zero reward. The first step of FMS computes the number of heavy atoms overlap between the query molecule node and the enforced building block (EBB). In the figure, this count is normalized by the EBB (divided by the number of heavy atoms in the EBB). In the main text, we denote this FMS_{Block}. FMS_{Query} denotes the case where the overlap is normalized by the query instead.

Supplementary Figure 2: TANGO Compute Time

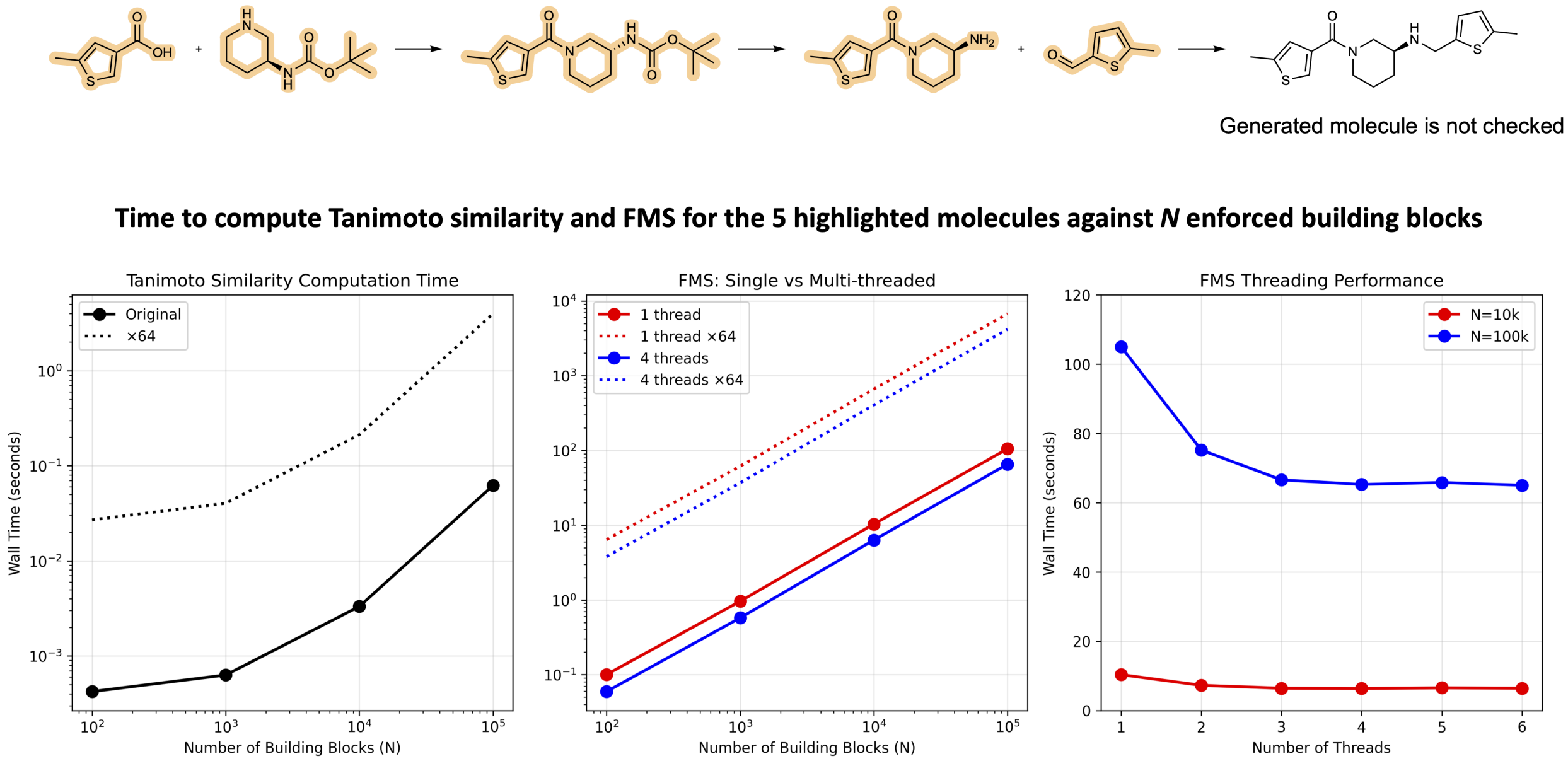

Supplementary Figure 2: Compute time for Tanimoto similarity and FMS across varying enforced building block set sizes (**N**). **Left:** Tanimoto similarity wall time. **Middle:** FMS wall time. **Right:** FMS threading performance for 10k and 100k set sizes.

Supplementary Figure 3: Docking Reward Shaping Function

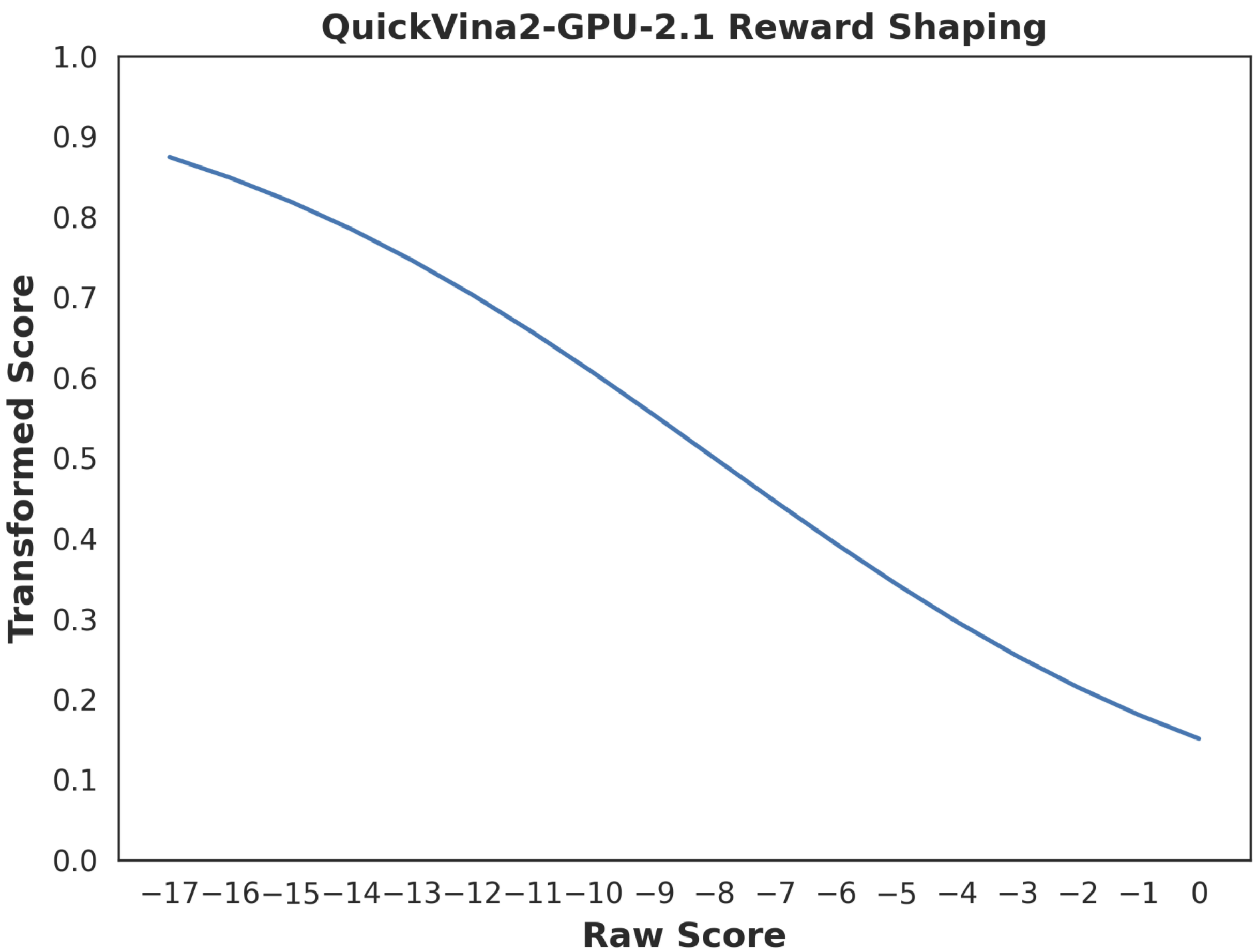

Supplementary Figure 3: Reward shaping function for docking.

Supplementary Figure 4: Reward Value Distributions for Different Reward Formulations

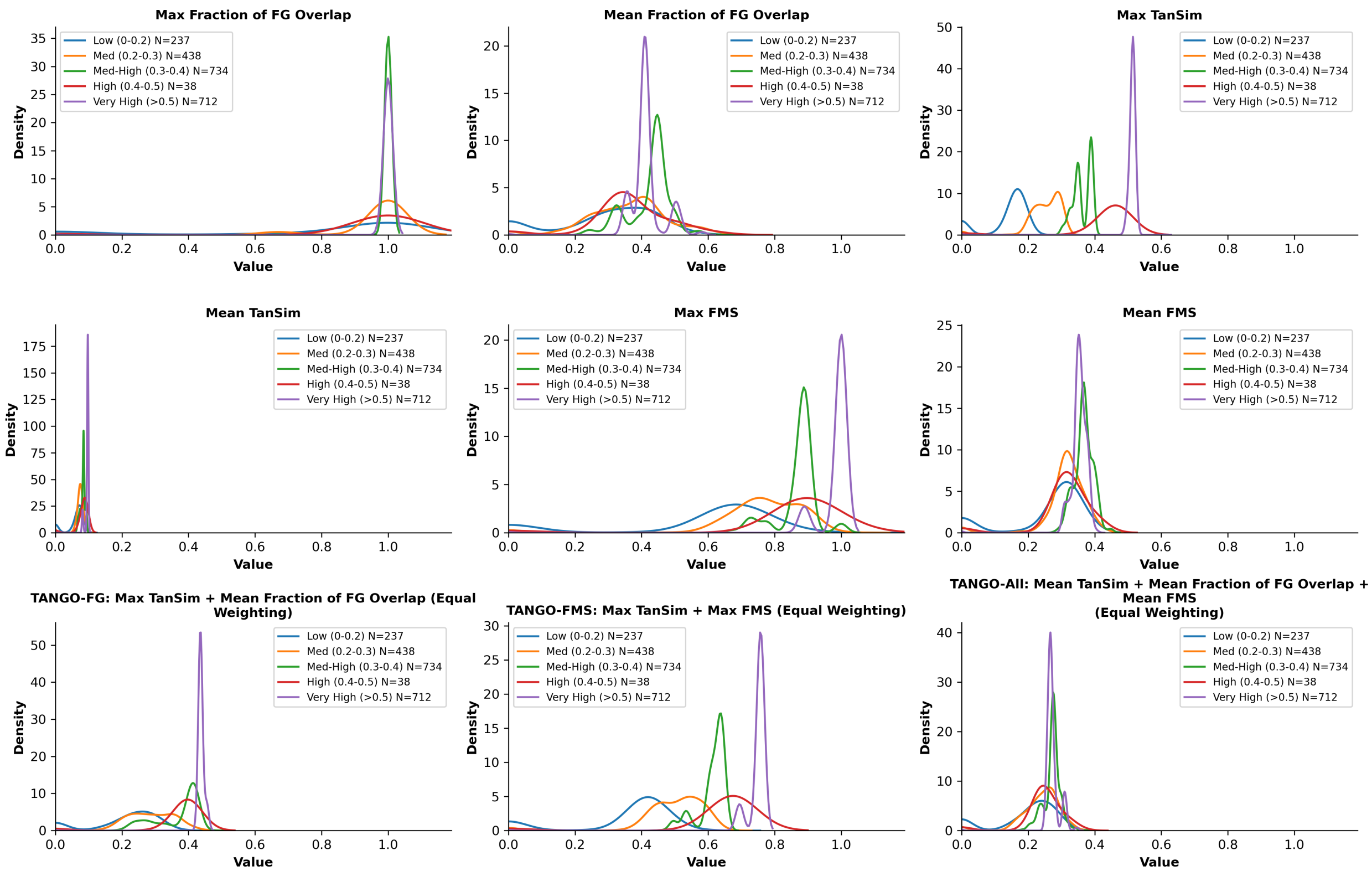

Supplementary Figure 4: Reward distributions of different reward function formulations. FMS here denotes FMS_{Block}

Supplementary Figure 5: Asymmetric Nature of Fuzzy Matching Substructure

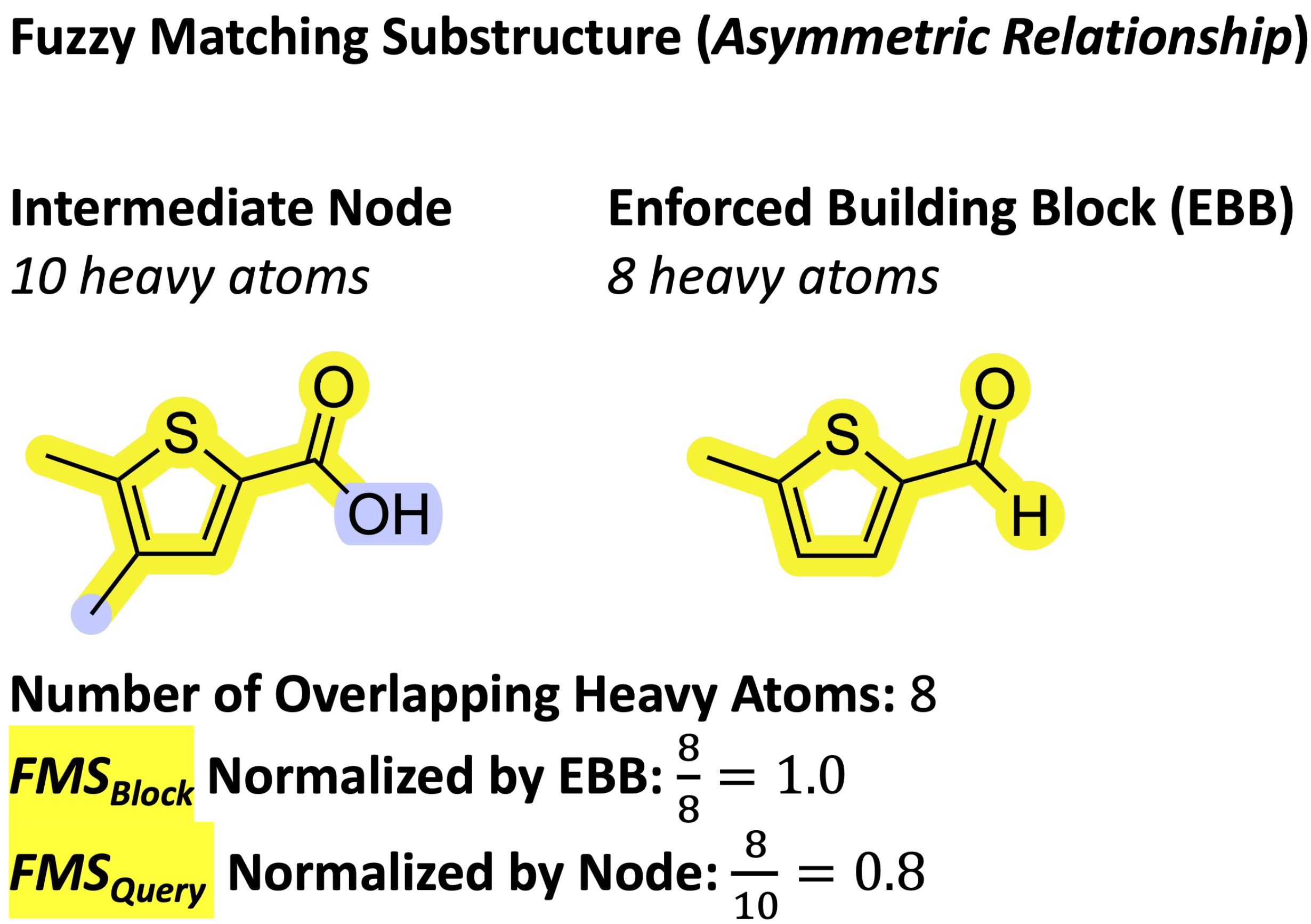

Supplementary Figure 5: Fuzzy Matching Substructure (FMS) is asymmetric depending on whether the number of matching atoms is divided by the number of atoms in the enforced building block or the intermediate node.

Supplementary Figure 6: 100 Blocks Purged (with QED) Example Synthesis Routes with Enforced Building Block Frequencies

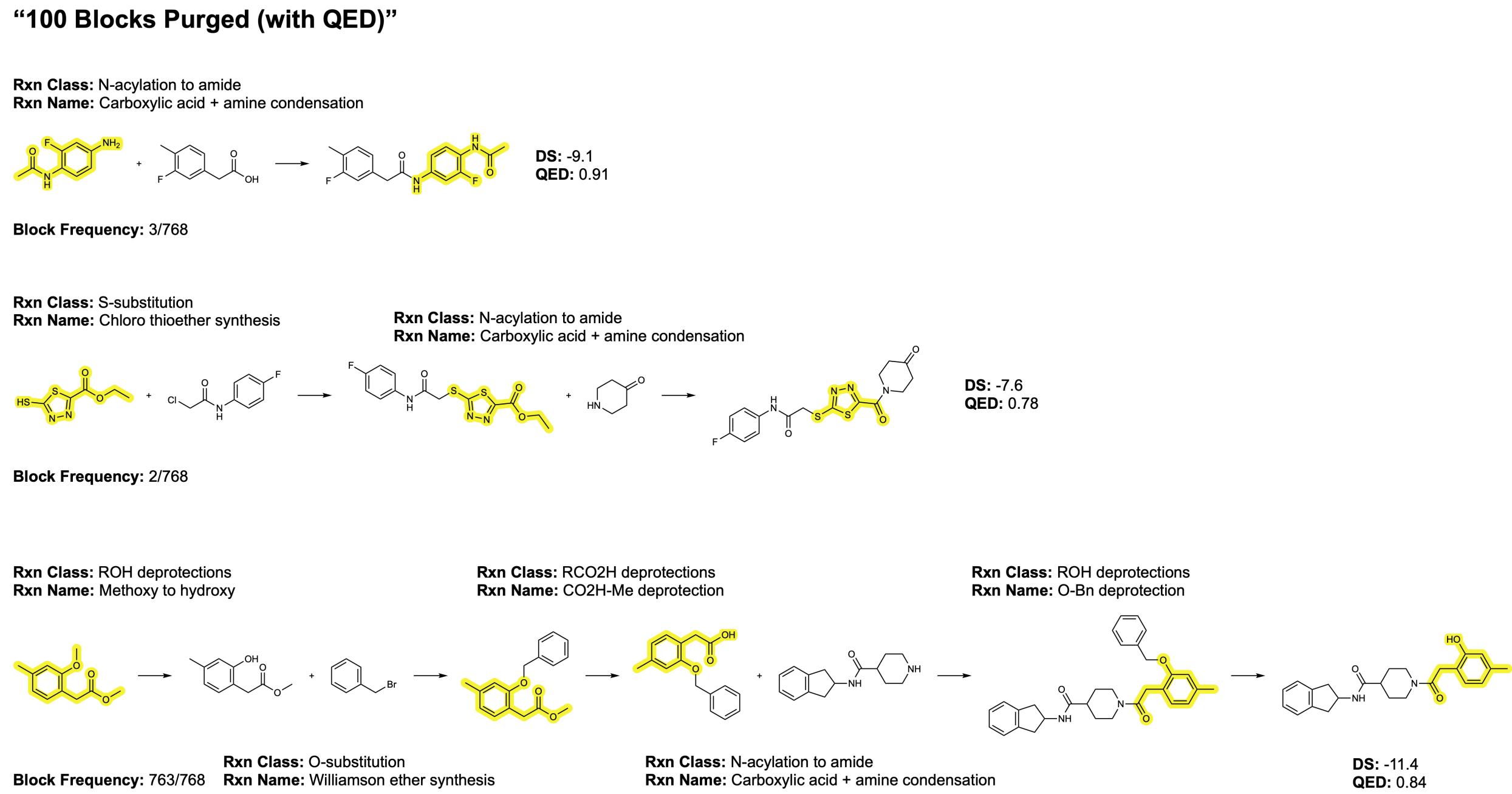

Supplementary Figure 6: "100 Blocks Purged (with QED)" synthesis routes of the best docking score molecule for each unique enforced building block in the pooled set. Reaction classes and names are annotated. The frequency of each block is also annotated. The specific enforced block is also highlighted throughout the synthesis, tracking its evolution.

Supplementary Figure 7: 100 Blocks Purged (without QED) Example Synthesis Routes with Enforced Building Block Frequencies

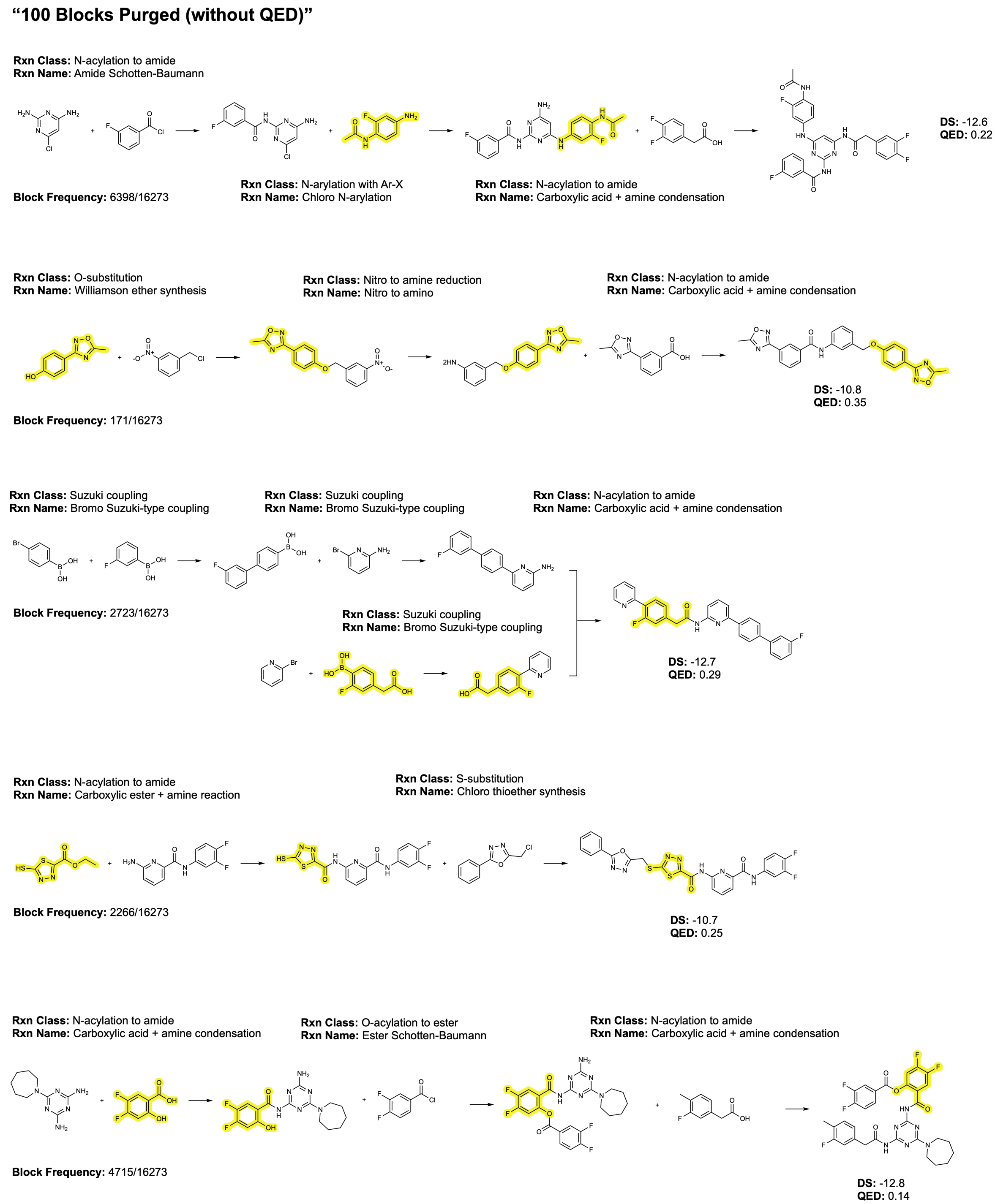

Supplementary Figure 7: "100 Blocks Purged (without QED)" synthesis routes of the best docking score molecule for each unique enforced building block in the pooled set. Reaction classes and names are annotated. The frequency of each block is also annotated. The specific enforced block is also highlighted throughout the synthesis, tracking its evolution.

Supplementary Figure 8: 5 Enforced Building Blocks Set

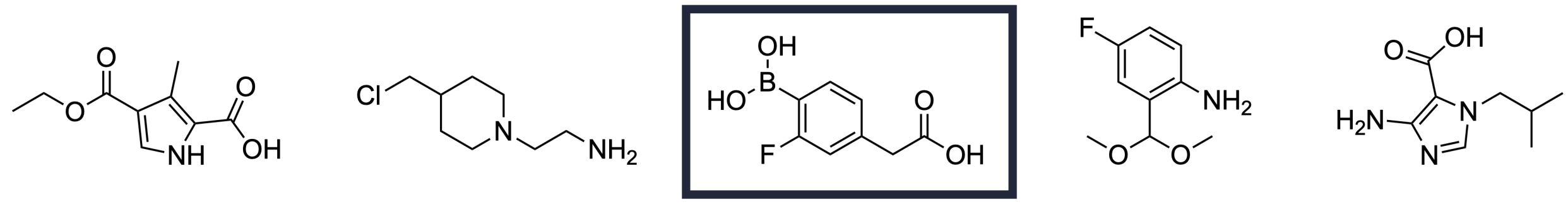

Supplementary Figure 8: 5 Enforced Building Blocks Set. The circled block is the Suzuki coupling reagent used in all the successful runs without QED (N=2/10 seeds).

Supplementary Figure 9: 100 Enforced Building Blocks Example Synthesis Routes

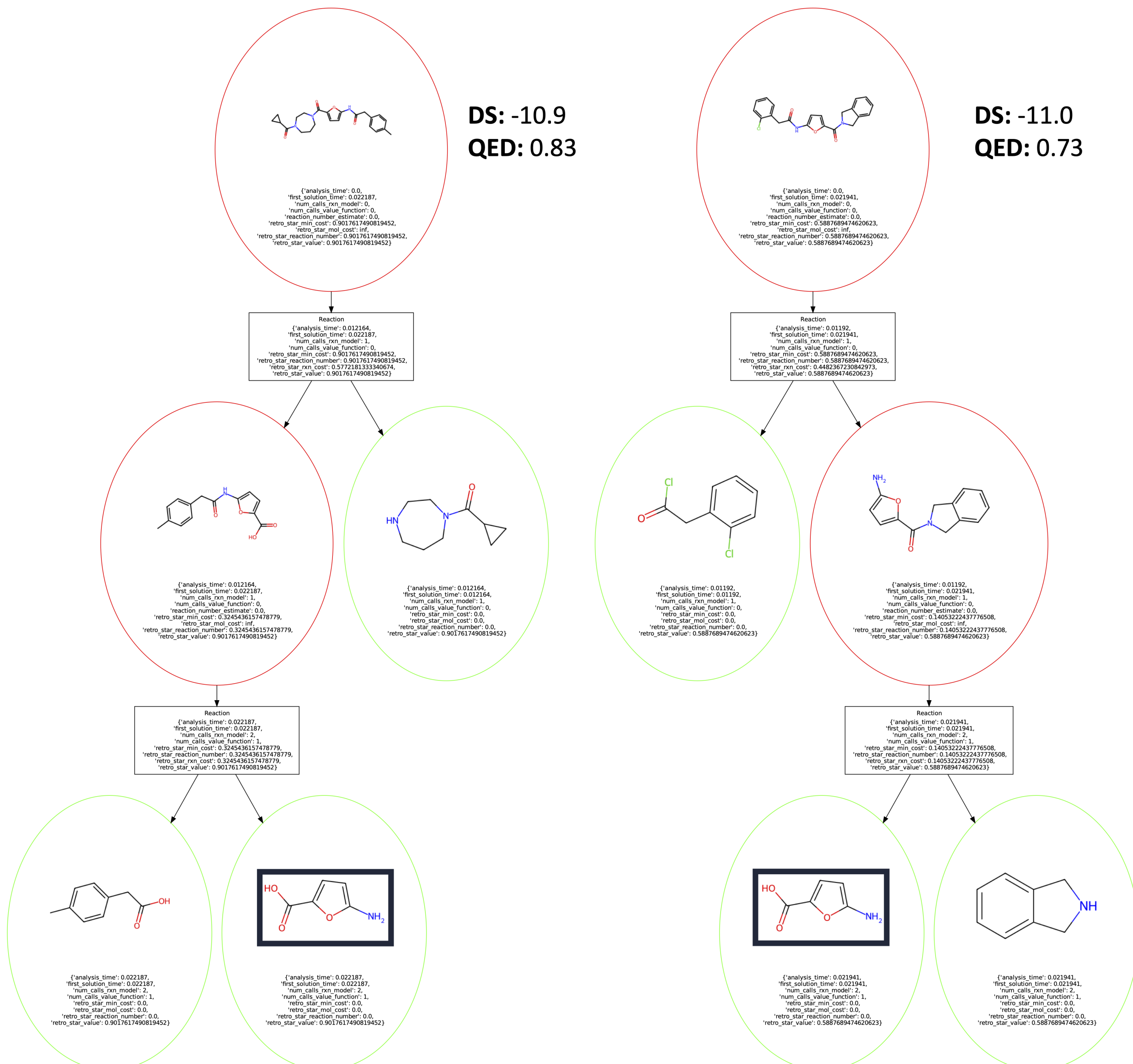

Supplementary Figure 9: 100 Enforced Blocks example routes. The enforced block is boxed. The annotated text in the boxes are the raw Syntheseus output and not related to the molecule structures.

Supplementary Figure 10: 100 Enforced Building Blocks Purged Example Synthesis Routes

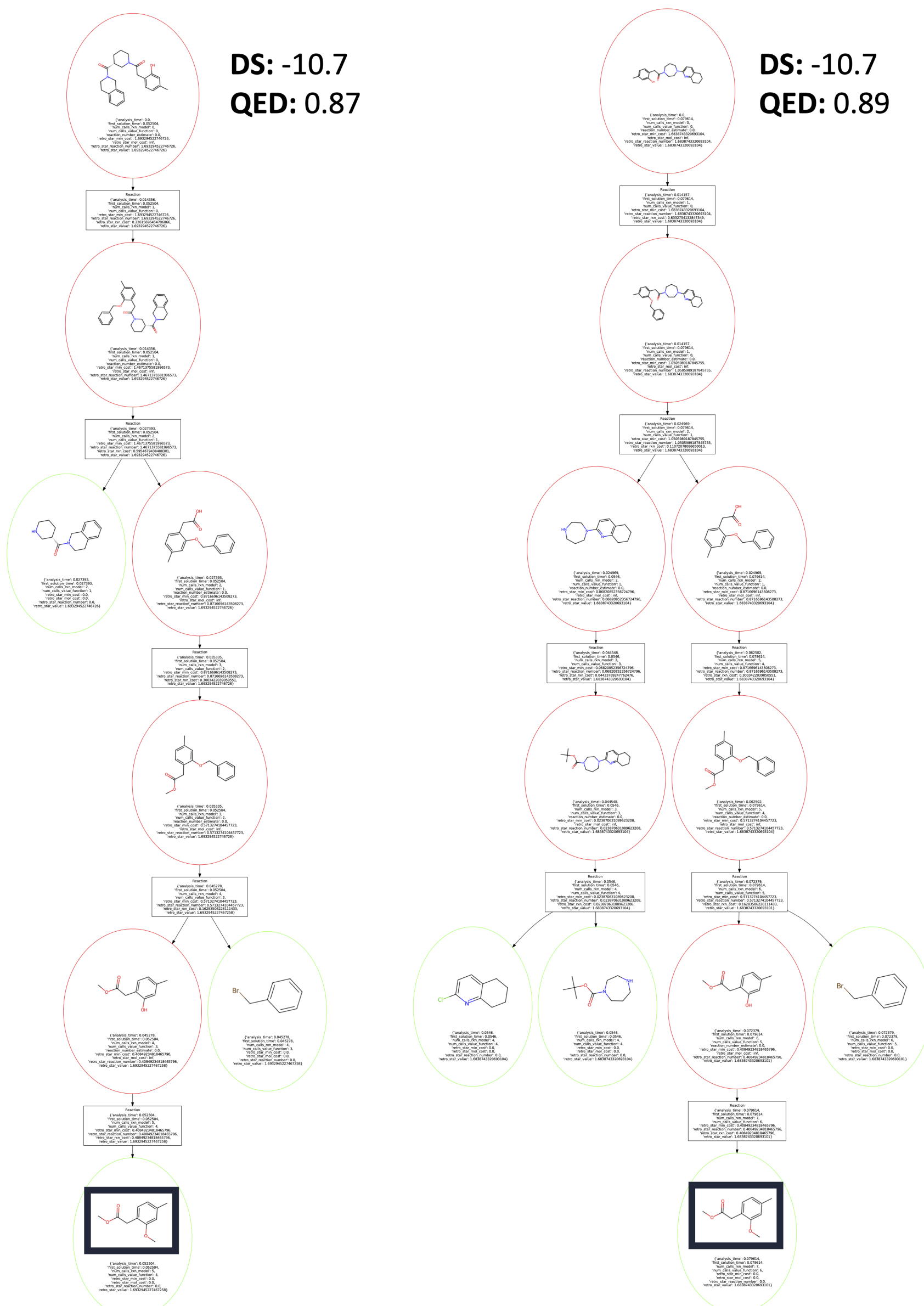

Supplementary Figure 10: 100 Enforced Blocks Purged example routes. The enforced block is boxed. The annotated text in the boxes are the raw Syntheseus output and not related to the molecule structures.

Supplementary Figure 11: 10 Enforced Building Blocks Example Synthesis Routes

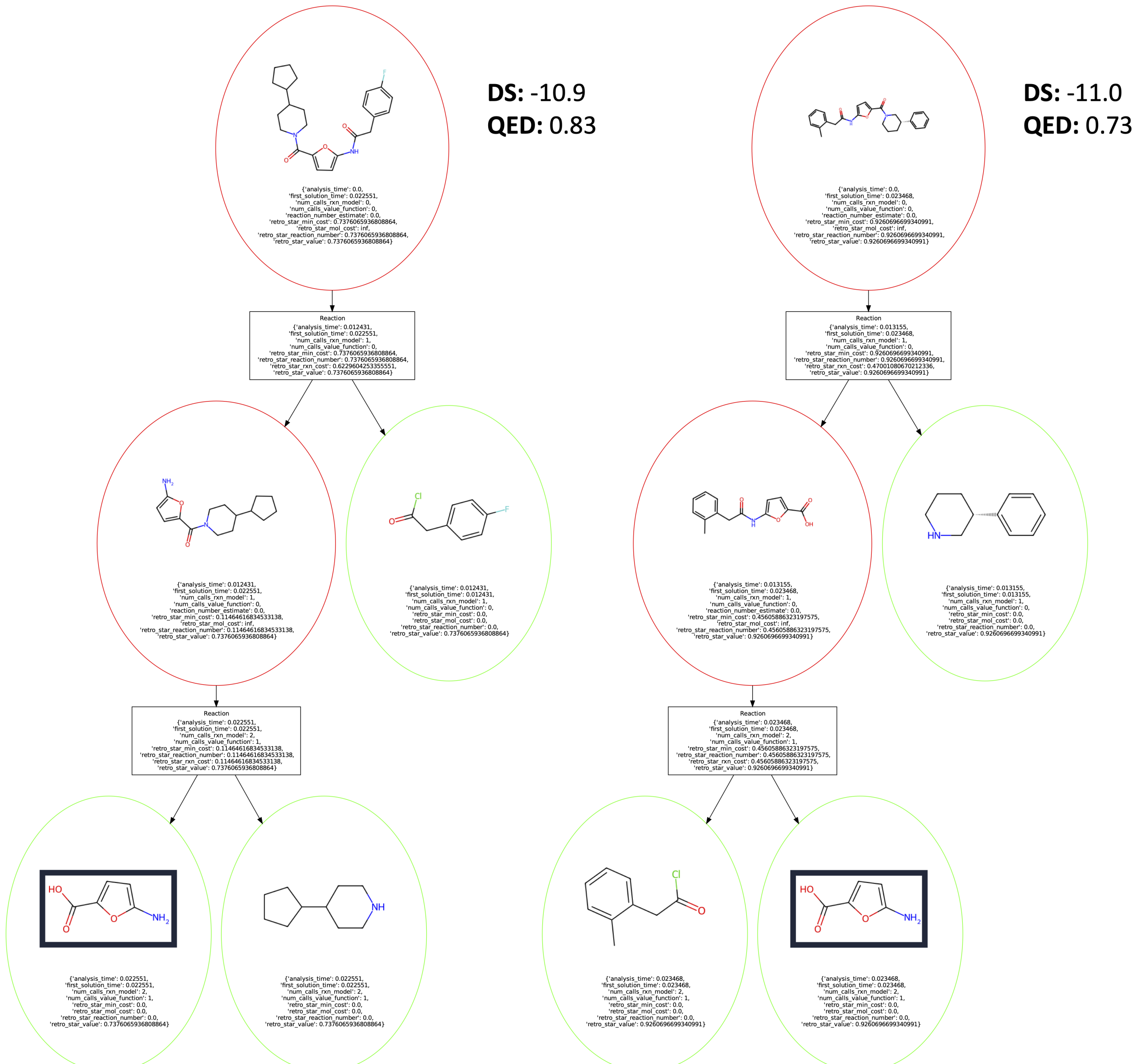

Supplementary Figure 11: 10 Enforced Blocks example routes. The enforced block is boxed. The annotated text in the boxes are the raw Syntheseus output and not related to the molecule structures.

Supplementary Figure 12: 5 Enforced Building Blocks Example Synthesis Routes

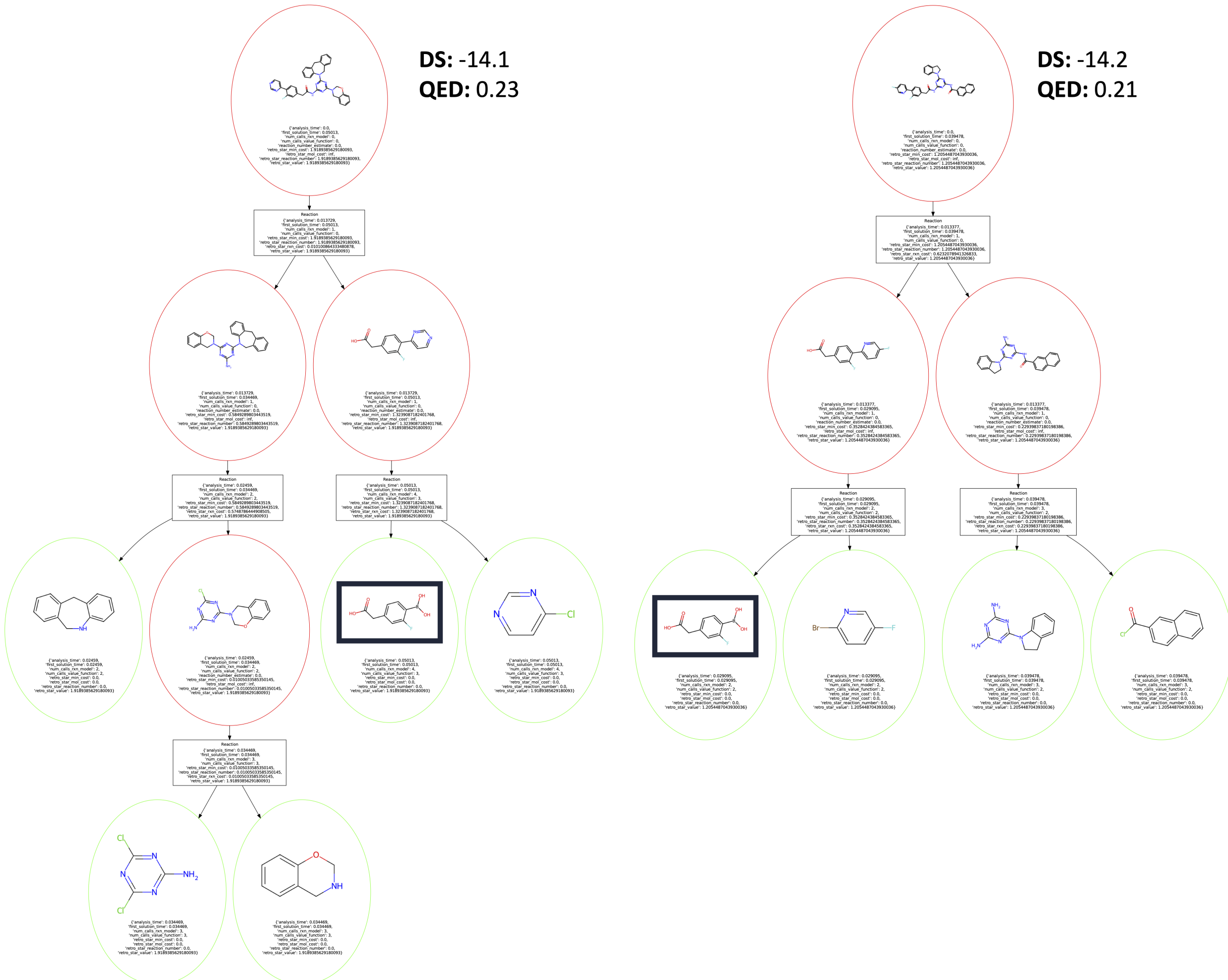

Supplementary Figure 12: 5 Enforced Blocks example routes. Note that QED was not enforced here as the QED experiments did not successful generate any enforced blocks under the oracle budget. The enforced block is boxed. The annotated text in the boxes are the raw Syntheseus output and not related to the molecule structures.

Supplementary Figure 13: Divergent Enforced Building Blocks Example Synthesis Routes

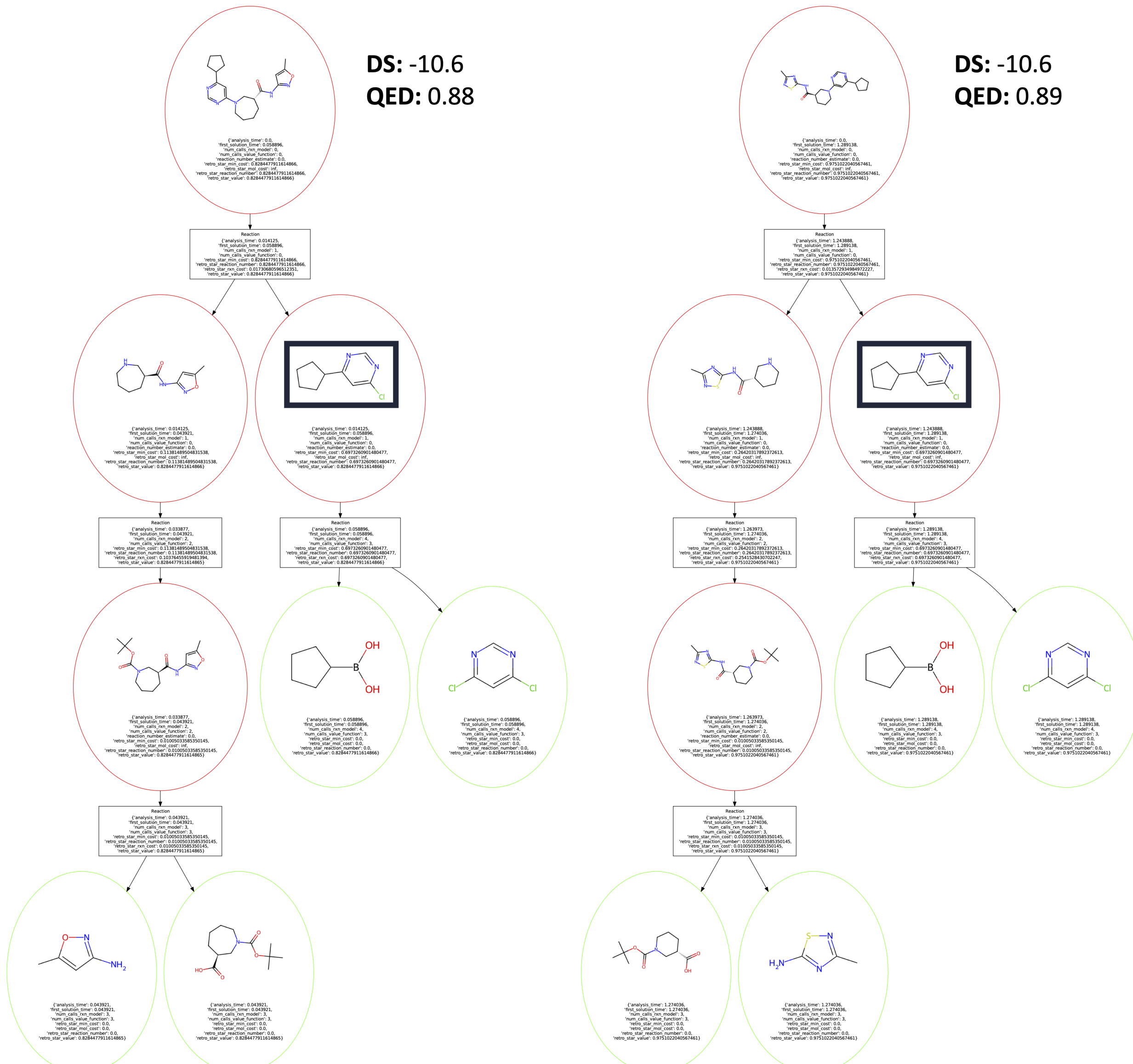

Supplementary Figure 13: Divergent Enforced Blocks example routes. The enforced block is boxed. The annotated text in the boxes are the raw Syntheseus output and not related to the molecule structures.

Supplementary Figure 14: Reaction Class Frequencies

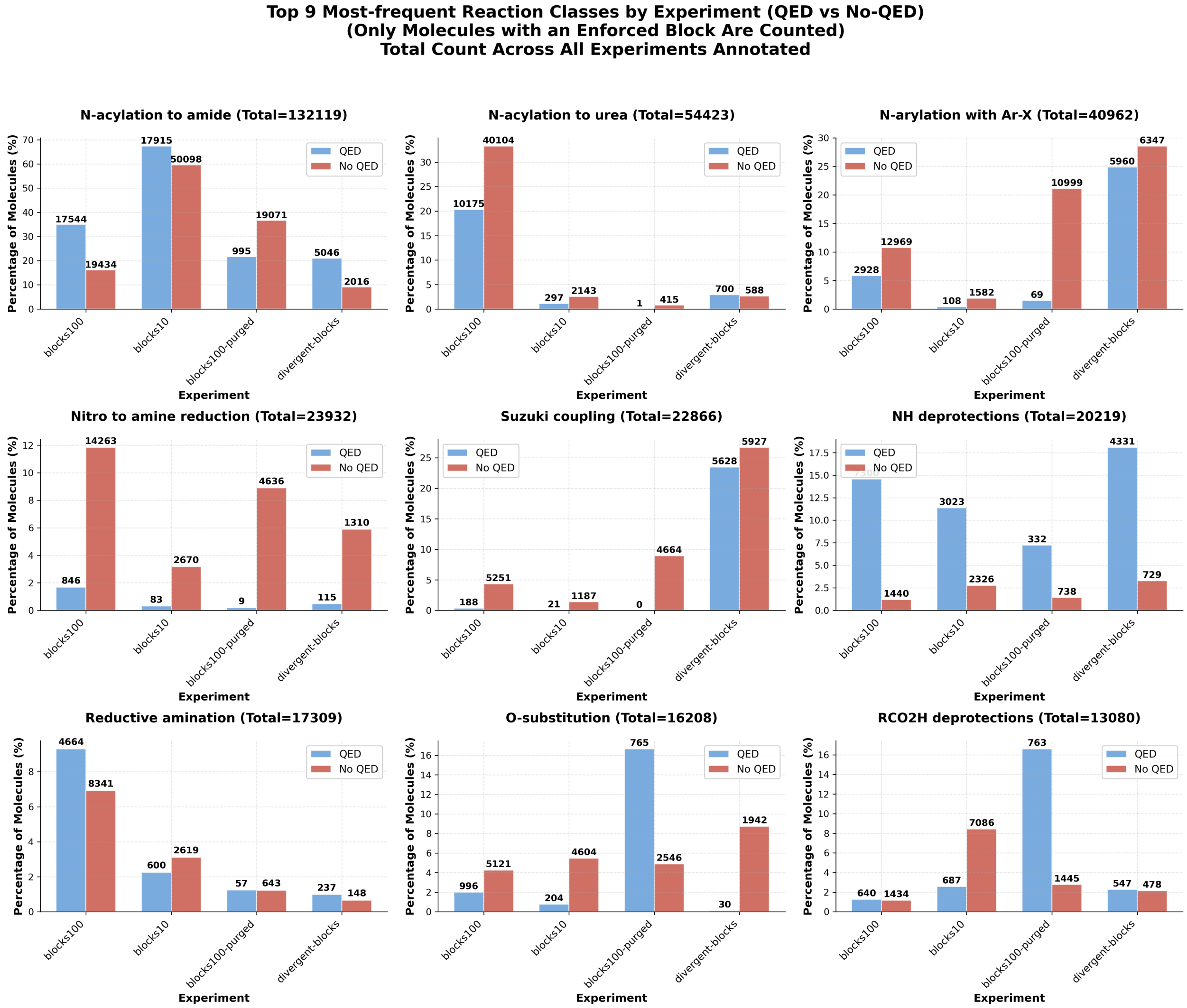

Supplementary Figure 14: Top-9 (across all experiments) reaction classes. For each experiment (with and without QED), the absolute number and percentage of the reaction's occurrence is shown. Reactions were labeled using NameRXN. Only molecules with a synthesis route containing an enforced building block are shown.

Supplementary Figure 15: Example Synthesis Routes with Two Enforced Building Blocks

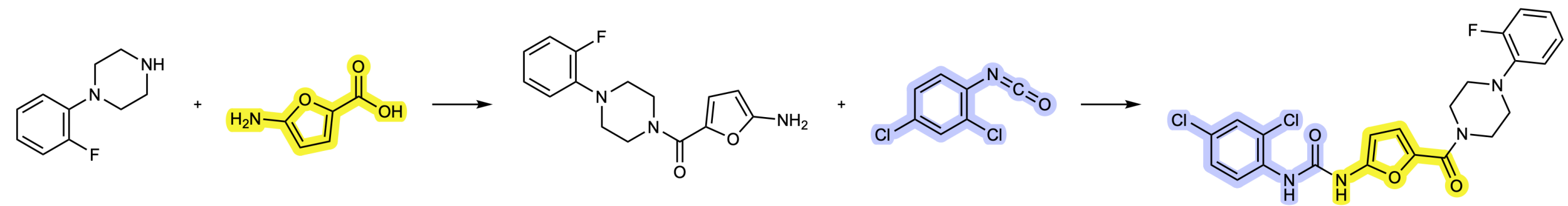

Supplementary Figure 15: An example generated molecule with a synthesis route enforced 2 building blocks (highlighted in yellow and peach).

References

[1] Guo, J., Sabanza Gil, V., *et al.* Generative Molecular Design with Steerable and Granular Synthesizability Control. *arXiv preprint arXiv:2505.08774* (2025).

[2] Guo, J. & Schwaller, P. Saturn: Sample-efficient generative molecular design using memory manipulation. *arXiv preprint arXiv:2405.17066* (2024).

[3] Gu, A. & Dao, T. Mamba: Linear-Time Sequence Modeling with Selective State Spaces. *COLM* (2024).

[4] Bjerrum, E. J. Smiles enumeration as data augmentation for neural network modeling of molecules. *arXiv preprint arXiv:1703.07076* (2017).

[5] Maziarz, K., *et al.* Re-evaluating retrosynthesis algorithms with syntheseus. *Faraday Discussions* **31** (2025).

[6] Sacha, M., *et al.* Molecule edit graph attention network: modeling chemical reactions as sequences of graph edits. *Journal of Chemical Information and Modeling* **61**, 3273–3284 (2021).

[7] Chen, B., *et al. RRetro*: Learning Retrosynthetic Planning with Neural Guided A* Search*, (Proc. 37th International Conference on Machine Learning, 2020).

[8] Sterling, T. & Irwin, J. J. Zinc 15–ligand discovery for everyone. *Journal of chemical information and modeling* **55**, 2324–2337 (2015).

[9] Genheden, S., *et al.* Aizynthfinder: a fast, robust and flexible open-source software for retrosynthetic planning. *Journal of cheminformatics* **12**, 70 (2020).

[10] Saigiridharan, L., *et al.* Aizynthfinder 4.0: developments based on learnings from 3 years of industrial application. *Journal of Cheminformatics* **16**, 57 (2024).

[11] Trott, O. & Olson, A. J. Autodock vina: improving the speed and accuracy of docking with a new scoring function, efficient optimization, and multithreading. *Journal of computational chemistry* **31**, 455–461 (2010).

[12] Alhossary, A., *et al.* Fast, accurate, and reliable molecular docking with quickvina 2. *Bioinformatics* **31**, 2214–2216 (2015).

[13] Tang, S., *et al.* Vina-gpu 2.1: towards further optimizing docking speed and precision of autodock vina and its derivatives. *IEEE/ACM Transactions on Computational Biology and Bioinformatics* (2024).

[14] Mabanglo, M. F., *et al.* Potent clpp agonists with anticancer properties bind with improved structural complementarity and alter the mitochondrial n-terminome. *Structure* **31**, 185–200 (2023).

[15] Bickerton, G. R., *et al.* Quantifying the chemical beauty of drugs. *Nature chemistry* **4**, 90–98 (2012).

[16] Coley, C. W., *et al.* Computer-assisted retrosynthesis based on molecular similarity. *ACS central science* **3**, 1237–1245 (2017).

[17] Zhang, Y., *et al.* Evolutionary retrosynthetic route planning. *IEEE Computational Intelligence Magazine* **19**, 58–72 (2024).

[18] Li, L., *et al.* Divergent strategy in natural product total synthesis. *Chemical reviews* **118**, 3752–3832 (2018).

[19] Castellino, N. J., *et al.* Late-stage functionalization for improving drug-like molecular properties. *Chemical reviews* **123**, 8127–8153 (2023).

[20] NextMove Software NameRxn 3.7.3.

[21] Benhenda, M. ChemGAN challenge for drug discovery: can AI reproduce natural chemical diversity?. *arXiv* (2017).

[22] Xie, Y., *et al.* How Much Space Has Been Explored? Measuring the Chemical Space Covered by Databases and Machine-Generated Molecules. *Proc. 11th International Conference on Learning Representations* (2023).

[23] Bemis, G. W. & Murcko, M. A. The Properties of Known Drugs. 1. Molecular Frameworks. *Journal of Medicinal Chemistry* **39**, 2887-2893 (1996).
