# Supplementary material for: TANGO: direct optimization of constrained synthesizability for generative molecular design
Source: Nat Comput Sci. 2026 Mar 3;6(3):260–70. doi: 10.1038/s43588-026-00959-1 (PMC13658032; doi:10.1038/s43588-026-00959-1)
Supplement: Supplementary file 2 [file 43588_2026_959_MOESM2_ESM.pdf]

nature portfolio

Peer Review File

It Takes Two to Tango: Directly Optimizing for Synthesizability in Generative Molecular Design

Corresponding Author: Professor Philippe Schwaller

This file contains all editorial decision letters in order by version, followed by all author rebuttals in order by version.

Version 0:

Decision Letter:

** Please ensure you delete the link to your author homepage in this e-mail if you wish to forward it to your co-authors. **

Dear Professor Schwaller,

Your manuscript "It Takes Two to Tango: Directly Optimizing for Synthesizability in Generative Molecular Design" has now been seen by 3 referees, whose comments are appended below. You will see that while they find your work of interest, they have raised points that need to be addressed before we can make a decision on publication.

The referees' reports seem to be quite clear. Naturally, we will need you to address *all* of the points raised.

While we ask you to address all of the points raised, the following points need to be substantially worked on:

1) Please provide clarifications and additional details as requested by reviewers.

Please use the following link to submit your revised manuscript and a point-by-point response to the referees' comments (which should be in a separate document to any cover letter):

Link Redacted

** This url links to your confidential homepage and associated information about manuscripts you may have submitted or be reviewing for us. If you wish to forward this e-mail to co-authors, please delete this link to your homepage first. **

To aid in the review process, we would appreciate it if you could also provide a copy of your manuscript files that indicates your revisions by making use of Track Changes or similar mark-up tools. Please also ensure that all correspondence is marked with your Nature Computational Science reference number in the subject line.

In addition, please make sure to upload a Word Document or LaTeX version of your text, to assist us in the editorial stage.

To improve transparency in authorship, we request that all authors identified as 'corresponding author' on published papers create and link their Open Researcher and Contributor Identifier (ORCID) with their account on the Manuscript Tracking System (MTS), prior to acceptance. ORCID helps the scientific community achieve unambiguous attribution of all scholarly contributions. You can create and link your ORCID from the home page of the MTS by clicking on 'Modify my Springer Nature account'. For more information please visit please visit www.springernature.com/orcid.

We hope to receive your revised paper within three weeks. If you cannot send it within this time, please let us know.

We look forward to hearing from you soon.

Best regards,

Kaitlin McCardle, PhD
Senior Editor

Nature Computational Science

Reviewers comments:

Reviewer #1 (Remarks to the Author):

The manuscript by Guo and Schwaller describes TANGO – a reward function to constrain synthesizability in automated molecular design.
The manuscript is very well written, extensively cites the relevant literature and I am convinced it is of high importance to the drug discovery sciences, in particular medicinal chemistry. The authors have comprehensively tested their tool, under various settings, and I would support publication of this fine study in Nat Comput Sci. However, I do have some minor suggestions that the authors might want to consider:
1) Possibly including instructions for a medicinal chemistry audience would be beneficial
2) I might have missed, but I did not find a clear motivation for using QED (a proxy for an ensemble of molecular properties) rather than a metric with a clear(er) physicochemical meaning (e.g. solubility)
3) The authors present a case study, but again, I missed for which target and can the authors contextualise their designed molecules relative to known ligands. Basically, I would like to understand if they make sense from a medicinal chemistry vantage point.

Again, this is a very nice study. Congratulations to the team!
Tiago

Reviewer #2 (Remarks to the Author):

The paper is a continuation of authors' work on generative molecular AI utilizing saturn software. In this contribution authors proposed a new reward functon(TANGO) which promotes synthesizable molecules. In their previous work, they use SAscore and AIZynth as a part of reward function which enforce synthesizability (R All MPO and R Double MPO in Chem. Sci., 2025, 16, 6943–6956). In this paper, they introduce a different approach that utilizes similarity to selected starting materials. I think the paper addresses a highly significant topic and is worth publishing; however, it requires substantial revisions in its current form.
I recommend that the authors rewrite the paper, taking into account the following comments and suggestions:

1. The QED abbreviation is not explained in maintext

2. Table and Figure from SI should be numbered in a way that does not overlapping with main text (e.g. S1, S2 and so on)

3. The TANGO function consists of two parts: FMS and TS (or TanSym). The FMS is not defined in main text, based on SI and code seems to me the FMS definition is: FMS = MCSS / n_heavy_block for MCSS / n_heavy_block < 1 or FMS = MCSS / n_heavy_query otherwise, where MCSS is number of heavy atoms in Max. Common SubStructure whereas n_heavy_block and n_heavy_query are the number of heavy atoms in building block and query molecule respectively. In Figure 3 from SI, first molecule: only 6 atoms are highlighted in yellow, As far as I understand 8 should be highlighted. In table 3 from SI there are "TANGO-FMS" which I am not sure is the same as "TANGO-FMS" from table 1 from SI and "TANGO-FMS Asymmetric-FMS" which seems to me is the same as "TANGO" in main text.

4. The second part of TANGO is TS (this abbreviation is used in equation 1) or TanSym (used in Fig 4), I guess these two names refer to the same term which is not fully defined neither in maintext nor in SI. Based on the source code it seems to me the tanimoto similarity is between the Morgan fingerprint with radius=2.

5. At this point, I would like to emphasize that it is extremely difficult to follow the authors' ideas when they do not define consistent terminology that is used throughout both the main text and the supplementary information (SI).

6. Morgan fingerprint and Tanimoto similarity are the most popular solution but there are many other options which may produce significantly different results. There are many other fingerprint types and method based of graph-convolution for molecular representation as well as similarity is not limited to Tanimoto (e.g. I have very promising experience with Tversky with carefully tuned parameters). I am really curious if changing these settings will improve the results for the TanSim reward function.

7. The definition of "Intermediate constraint" is unclear. Figure 1 suggests that it refers to starting materials used in the second or later steps of synthesis, whereas the definition in "Case 2" implies it can be a starting material used at any stage of synthesis. The term 'intermediate' in organic synthesis typically refers to compounds that are not starting materials but are produced in one step and utilized in another. Therefore, regardless of which definition is correct, the term is misleading.

8. The definition of "Divergent synthesis" is not clear to me as well. Fig1 presents one synthetic graph to 4 products which has one common compound and each product requires at least one commercial building block for preparation. In 'Case 3' is a defined graph which has an intermediate for which all building blocks (leafs in sub-graph) are non-commercial. From 'Case 3' it is not clear for me how the constraint produces a graph with several products, as presented in Fig. 1.

9. The authors focus on enforcing the presence of selected building blocks (Benf) in synthetic graphs and claim, based on references [20-22], that in real-world applications, Benf is small. In the cited papers, the set of allowed building blocks was indeed limited, but this corresponds to B (the set of all allowed initial building blocks) rather than Benf. From my understanding of the real-world application of Computer-Aided Synthesis Planning (CASP), the enforcement of selected compound(s) in a synthetic pathway is target-dependent, and this information is provided to guide the algorithm when general searches (without restrictions) yield unsatisfactory results. Selecting Benf as a random set of simple molecules seems illogical. Instead, I suggest defining Benf as a set of compounds that possess important structural motifs relevant to the target or promote specific reactions along the pathway. For instance, Benf could consist of natural amino acids as a cheap source of chiral centers, allowing for the analysis of chirality sources in the resulting products.

10. The FMS part of TANGO requires calculation of MCSS between all generated molecules and all enforced building blocks (EBB) which suggest it will be really time consuming for bigger EBB sets or compounds with many atoms. Could you please comment how this method scales with respect to molecule size and number of EBB?

11. Configurations from SI Table 3 (especially FG and brutal-force) should be defined.

12. In most cases obtained paths are short (1-4 steps) and based on Commonly Enforced Blocks. It is worth discussing the diversity of obtained products in the sense of chemical structure (e.g. using fingerprint similarity and Murcko scaffolds), physical property (mass, logP, total PSA and so on) and synthetic paths (what type of reaction is used).

13. Results from “100 Blocks Purged“ experiments are very interesting, obtained paths are longer (3-7 steps) which suggests obtained compounds can be more diverse. I think authors should also analyze obtained compounds and paths. Is there a new “lucky” EBB?

Reviewer #2 (Remarks on code availability):

I see the source code and it is more understandable than both the main text and the supplementary information (SI).

Reviewer #3 (Remarks to the Author):

Summary of the contributions:
Designing molecules that satisfy multi-parameter optimization objectives, while being synthesizable and exhibiting desirable properties (e.g., binding affinity to targets), remains a critical challenge in drug discovery. To address this, this work introduces a novel continuous reward function, TANGO, and integrates it into molecular generative models (e.g., SATURN) to discover molecules that meet multi-objective requirements. TANGO is versatile and can handle various constraints, such as starting materials, intermediates, and divergent synthesis pathways. Experimental results demonstrate that: 1) TANGO achieves better and more robust performance compared to simple Tanimoto Similarity (TanSim) and Fuzzy Matching Substructure (FMS), and 2) reinforcement learning with the TANGO reward enables the generative model to explicitly learn a desirable molecular distribution.

Questions:
1) In Figure 1, the roles of M and N (especially how it relates to the reward model's performance) are not entirely clear to me. Could the authors reorganize the content to present it in a clearer and more comprehensible manner?
2) Why is an oracle budget of 10,000 chosen for running the retrosynthesis search library? Does varying the oracle budget significantly impact the reward function? I believe that with different oracle budgets, the synthesis graph of the generated molecule would differ, which would, in turn, affect the TANGO reward. Is this correct?
3) Probably wrong usage of the terms 'Sparse' and 'Dense' in the context of reinforcement learning. In this domain, a Dense reward typically refers to scenarios where the reward function provides detailed feedback at every step or action in an episode. However, in this case, the authors might mean that TANGO is more continuous rather than binary.

Reviewer #3 (Remarks on code availability):

Yes, the codebase is clearly structured and well-documented. I was able to install and run the code successfully.

Version 1:

Decision Letter:

Our ref: NATCOMPUTSCI-25-1818A

22nd October 2025

Dear Dr. Schwaller,

Thank you for submitting your revised manuscript "It Takes Two to Tango: Directly Optimizing for Synthesizability in Generative Molecular Design" (NATCOMPUTSCI-25-1818A). It has now been seen by the original referees and their

comments are below. The reviewers find that the paper has improved in revision, and therefore we'll be happy in principle to publish it in Nature Computational Science, pending minor revisions to satisfy the referees' final requests and to comply with our editorial and formatting guidelines.

We are now performing detailed checks on your paper and will send you a checklist detailing our editorial and formatting requirements in about a week. Please do not upload the final materials and make any revisions until you receive this additional information from us.

TRANSPARENT PEER REVIEW
Nature Computational Science offers a transparent peer review option for original research manuscripts. We encourage increased transparency in peer review by publishing the reviewer comments, author rebuttal letters and editorial decision letters if the authors agree. Such peer review material is made available as a supplementary peer review file. **Please remember to choose, using the manuscript system, whether or not you want to participate in transparent peer review.**
Please note: we allow redactions to authors' rebuttal and reviewer comments in the interest of confidentiality. If you are concerned about the release of confidential data, please let us know specifically what information you would like to have removed. Please note that we cannot incorporate redactions for any other reasons. Reviewer names will be published in the peer review files if the reviewer signed the comments to authors, or if reviewers explicitly agree to release their name. For more information, please refer to our FAQ page.

Thank you again for your interest in Nature Computational Science. Please do not hesitate to contact me if you have any questions.

Sincerely,

Kaitlin McCardle, PhD
Senior Editor
Nature Computational Science

ORCID
IMPORTANT: Non-corresponding authors do not have to link their ORCIDs but are encouraged to do so. Please note that it will not be possible to add/modify ORCIDs at proof. Thus, please let your co-authors know that if they wish to have their ORCID added to the paper they must follow the procedure described in the following link prior to acceptance:
https://www.springernature.com/gp/researchers/orcid/orcid-for-nature-research

Author names using non-Roman characters
Nature Portfolio journals can support presentation of author names using non-Roman characters in the HTML version of the article. If you wish to, please include author names in parentheses after the Roman-character spelling; see example online here. Currently supported scripts are: Arabic, Chinese, Cyrillic, Devanagari, Greek, Hebrew, Hangul, Japanese and Persian. You will be asked to verify the rendering is correct at proof stage.

Reviewer #1 (Remarks to the Author):

The authors have revised their manuscript quite comprehensively. This is indeed a very nice study that, in my opinion, is not only very well executed but is also relevant to a wide audience in the chemical sciences. Therefore, I recommend publication in Nat Comput Sci.

Reviewer #2 (Remarks to the Author):

I sincerely appreciate the authors' efforts to address all of my comments and questions. I would like to clarify a misunderstanding regarding Figure S4: the left-hand side molecule has marked four atoms. Each line covers two atoms: the first represents the methyl group and aromatic carbon, and the second includes the hydroxyl group and the carbon from the carbonyl group.

Regarding the new content, I have a few minor points to discuss:

1) For the intDiv1 measure, the paper by Aspuru-Guzik et al. is cited. However, the paper cite arxiv.org/pdf/1708.08227 as source of intDiv1.

2) The definition of the #Circles measure is given as #Circles(S,d,t), where S is the set of molecules, d is the distance measure (I guess in the paper it is Tanimoto), and t represents the threshold. It seems that t can be arbitrarily chosen, but this is not explicitly stated in the text. For clarity, it would be better to include the definitions for both intDiv1 and #Circles in the supplementary information.

3) I recommend that the authors double-check the references, as there are some inconsistencies. For example, intDiv1 is mentioned for the first time on page 5 but is cited on page 24. Additionally, while reference 91 lists 16 authors, all of whom

are explicitly named, reference 92 only has four authors but is cited as Xie, Y. et al..

Thank you for considering these points.

Reviewer #2 (Remarks on code availability):

I did see the code during review of 1st version of the paper

Reviewer #3 (Remarks to the Author):

Thank you to the authors for addressing all my questions. The draft is in good shape now, and I recommend acceptance.

Reviewer #3 (Remarks on code availability):

The codebase provides a clear README file, and I am able to install the code.

Open Access This Peer Review File is licensed under a Creative Commons Attribution 4.0 International License, which permits use, sharing, adaptation, distribution and reproduction in any medium or format, as long as you give appropriate credit to the original author(s) and the source, provide a link to the Creative Commons license, and indicate if changes were made.
In cases where reviewers are anonymous, credit should be given to 'Anonymous Referee' and the source.
The images or other third party material in this Peer Review File are included in the article's Creative Commons license, unless indicated otherwise in a credit line to the material. If material is not included in the article's Creative Commons license and your intended use is not permitted by statutory regulation or exceeds the permitted use, you will need to obtain permission directly from the copyright holder.
To view a copy of this license, visit https://creativecommons.org/licenses/by/4.0/

General Response to reviewers

We would like to thank the reviewers for their constructive feedback, especially around ambiguities and the practicality of the proposed model. We first highlight all general changes and additions to the manuscript. Whenever we cross-reference excerpts/sentences we wrote from the manuscript, we add gray highlighting.

General Changes

- Previously, the results tables reported the # generated molecules across different docking thresholds, where **M** was the number of pooled molecules (across seeds) meeting each threshold. Following reviewer 3's feedback, we now improve clarity by reporting:
    1. The mean and standard deviation across the seeds for all metrics
    2. Pool all molecules (still across all seeds) and report the same metrics on this pooled set
- Following reviewer 2's feedback on including diversity metrics, we now add the **Internal Diversity (IntDiv1)** using Morgan fingerprints (radius=2, nBits=1024), **#Circles** (sphere packing), and the **# Bemis-Murcko scaffolds**. We additionally add the **Ligand Efficiency** metric defined by the docking score / # heavy atoms.
- Below, we copy a snippet of this new table format. While there is now more information in the table, we hope the reviewers find the information useful, thorough, and clear. All tables consist of a "top" and "bottom". The top is always the following format:

**Configuration**<br>**N:** # seeds/5 generating $\geq$ 1 Solved (Enforced)	**Synthesizability**			**Solved (Enforced) Molecules**<br>**Reaction Values:** Pooled (**M** Pooled Molecules)<br>**Unique Enforced Block Values:** Per-seed		**Oracle Budget**<br>**(Wall Time)**
	**Non-solved**	**Solved**	**Solved (Enforced)**	**# Reaction Steps**	**# Unique Enforced Blocks**	
TanSim (N=3)	2275 $\pm$ 143	7653 $\pm$ 142	1864 $\pm$ 1828	2.21 $\pm$ 1.16 (M=8731)	1.33 $\pm$ 0.47	10,000<br>(9h 11m $\pm$ 48m)

    - The metrics are unchanged but we are now more clear that:
      * **N:** # seeds generating at least 1 molecule with an enforced block (Solved (Enforced) metric)
      * **M:** # of pooled molecules across all seeds

The bottom table is always the following format:

**Configuration** **N:** # seeds/5 generating $\geq$ 1 Solved (Enforced)	**Molecule Metrics for Solved (Enforced) Molecules** **Top Values:** Per-seed **Bottom Values:** Pooled (**M** Pooled Molecules)					
	**Docking Score**	**QED**	**Ligand Efficiency**	**IntDiv1**	**#Circles**	**# Scaffolds**
TanSim (N=3)	-7.87 $\pm$ 0.47	0.81 $\pm$ 0.09	0.32 $\pm$ 0.01	0.60 $\pm$ 0.01	1.33 $\pm$ 0.58	2477 $\pm$ 1488
	-7.74 $\pm$ 0.81 (M=8731)	0.81 $\pm$ 0.08	0.31 $\pm$ 0.04	0.67	3	6840

- The table makes it clear that the top row of values are per-seed and bottom are pooled. We report both for thorough statistics

- During the revision period, we found that in rare cases, a generated molecule can contain more than 1 enforced block in its synthesis route, despite not being rewarded to do so. This occurs very infrequently but we report the frequencies in the new Supplementary Information Section "Generated Molecules With > 1 Enforced Building Block". We show an example route of such a molecule below with the enforced blocks highlighted in yellow and peach:

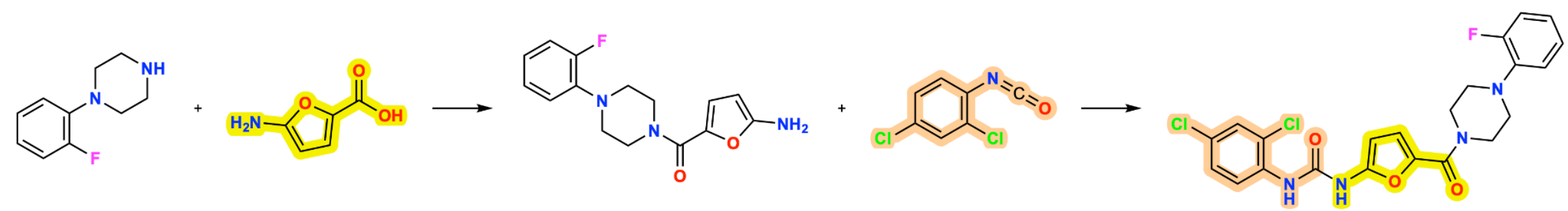

This figure has also been added to the Supplementary Information):

<u>New Results Section</u>

COX2 Case Study: Enforcing Phenylsulfonamide Building Blocks

A new set of 139 phenylsulfonamide-containing enforced building blocks was used in this new results section. We have updated our data share with the SMILES list:
<u>https://figshare.com/s/07867a1e1025c19 2e6a9</u>

This new section was added based on feedback from reviewers 1 and 2:

- Contextualizing the plausibility of generated molecules as binders
- The utility of the specific enforced blocks as in our development of TANGO, we randomly sampled building blocks and designated them as enforced building blocks. We did this for method development and we agree there are use cases where we want to be more intentional with the exact enforced building blocks

Experimental Context
(bullet-points as full section is in the updated manuscript):

- Selective COX2 (over COX1) inhibitors can be used to treat inflammation
- Existing known drugs and binders commonly feature a phenylsulfonamide moiety which has been shown to contribute to selectivity in binding COX2 over COX1
- We extract all 139 phenylsulfonamide-containing building blocks from our commercial building blocks stock (based on ZINC)
- We use TANGO to design potential selective COX2 inhibitors by enforcing phenylsulfonamide-containing blocks

Results (bullet-points as full section is in the updated manuscript):

- We first copy the new figure below:

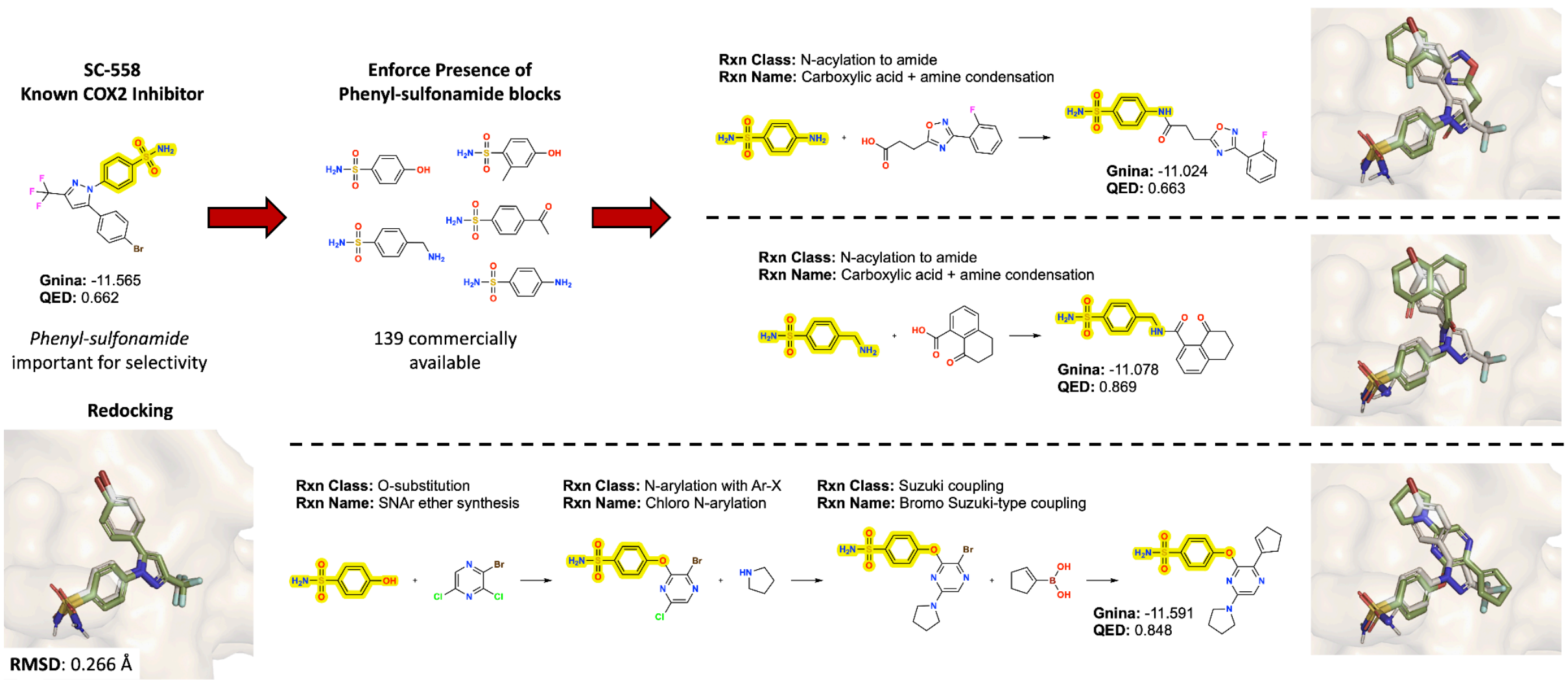

- First, re-docking with Gnina of SC-558 (reference ligand) reasonably reproduces the binding pose
- Generated molecules enforcing the phenylsulfonamide blocks contain phenylsulfonamide, as expected
- The docking pose of these generated molecules keep the phenylsulfonamide in a similar orientation as SC-558 and are plausible binders
- Further analysis of the reactions (labeled using NameRXN) shows that there are sometimes even 1-step amide coupling reactions

We thank the reviewers for their interest and critical feedback on the utility of the enforced building blocks. We hope this new experiment demonstrates a practical application.

Reviewer #1:

We want to first thank the reviewer for their positive assessment of our work and are grateful for their interest in TANGO. We reply to each question point-by-point.

Q1: Possibly including instructions for a medicinal chemistry audience would be beneficial.

A1: Thank you for this suggestion! We are planning to release a graphical user interface (GUI) in the near future. Our goal is to (1) Allow chemists to run the TANGO framework (*and more capabilities*) with minimal coding required and (2) Facilitate easier synthesis route visualization.

Q2: I might have missed, but I did not find a clear motivation for using QED (a proxy for an ensemble of molecular properties) rather than a metric with a clear(er) physicochemical meaning (e.g. solubility)

A2: Thank you for this critical comment. In the original manuscript submission, we focused all experiments on demonstrating method development. We chose QED mainly as a straightforward approach to constrain the molecular weight and lipophilicity. In this way, "good" docking molecules cannot be extremely large, greasy molecules, which would have a higher likelihood of being promiscuous binders. We agree with the reviewer that decomposing QED into specific constraints can be more meaningful but we believe this could be project-specific. For this reason, we focused only on QED.

Q3: The authors present a case study, but again, I missed for which target and can the authors contextualise their designed molecules relative to known ligands. Basically, I would like to understand if they make sense from a medicinal chemistry vantage point.

A3: In the original manuscript submission, we performed docking using QuickVina2-GPU against ATP-dependent Clp protease proteolytic subunit (ClpP). We chose this target solely based on previous work on synthesizable molecular generative design that used this case study:
https://arxiv.org/abs/2406.08506v2

We adapted their docking objective as the reward function for method development.

Here, to respond to the reviewer's question, we decided to run a new real-world-inspired case study and thank the reviewer for their comment. We mimicked a design campaign to fix a specific chemical moiety. The new results demonstrate how TANGO can design synthesis routes using building blocks that install this exact moiety. We kindly refer the reviewer to the General Response here.

Reviewer #2

We first thank the reviewer for their critical feedback. The additional experiments we ran to address their comments was a rewarding learning experience for us!

Before we begin our point-by-point response to the reviwer, we wanted to emphasize that this TANGO work is distinct from our previous work using AiZynth as the reward function:
https://pubs.rsc.org/en/content/articlehtml/2025/sc/d5sc01476j
In that work, we were focused on generating molecules with ***any*** synthesis route. TANGO tackles the ***constrained synthesizability*** setting which is much more challenging, as the synthesis route ***must also contain a specific enforced block***.

Q1: The QED abbreviation is not explained in the main text.

A1: Thank you for highlighting this. We now introduce QED as the quantitative estimate of drug-likeness on first mention in the text. This change affects the **Results** and **Methods** sections and is highlighted.

Q2: Table and Figure from SI should be numbered in a way that does not overlapping with main text (e.g. S1, S2 and so on)

A2: We have implemented this change.

Q3: The TANGO function consists of two parts: FMS and TS (or TanSym). The FMS is not defined in main text, based on SI and code seems to me the FMS definition is: FMS = MCSS / n_heavy_block for MCSS / n_heavy_block < 1 or FMS = MCSS / n_heavy_query otherwise, where MCSS is number of heavy atoms in Max. Common SubStructure whereas n_heavy_block and n_heavy_query are the number of heavy atoms in building block and query molecule respectively. In Figure 3 from SI, first molecule: only 6 atoms are highlighted in yellow, As far as I understand 8 should be highlighted. In table 3 from SI there are "TANGO-FMS" which I am not sure is the same as "TANGO-FMS" from table 1 from SI and "TANGO-FMS Asymmetric-FMS" which seems to me is the same as "TANGO" in main text.

A3: Thank you to the reviewer for their thorough analysis of the code. We apologize for our inconsistent naming and insufficient clarity. We respond to this comment in 2 parts:

1. What we have done to improve the clarity of what FMS is

We acknowledge that the meaning of "TANGO-FMS" and "TANGO-FMS Asymmetric-FMS" was convoluted. To improve clarity, we now explicitly define FMS at the start of the Results section. Moreover, "TANGO-FMS" is now **TANGO-FMS_Block** and "TANGO-FMS Asymmetric-FMS" is now **TANGO-FMS_Query**. We copy the newly added excerpt below (it is highlighted in the updated manuscript):

"We define Fuzzy Matching Substructure (FMS) as the number of heavy atoms overlap between a query molecule (molecule node in a synthesis graph) and an enforced building block (EBB). FMS_Block and FMS_Query denote whether the heavy atoms overlap is normalized by the EBB or query molecule, i.e., divided by the number of heavy atoms in the EBB or query molecule, respectively. Both FMS formulations yield a continuous signal of similarity just like Tanimoto similarity. In early experiments, we found that FMS_Block can lead to false positives in the edge case that the query molecule contains the EBB as a substructure but has other additional atoms, i.e., the query molecule is a superset of the EBB (see the Supplementary Information for an illustrative example). This edge case is handled by using FMS_Query which normalizes the heavy atoms overlap by the query molecule. Note that the converse is possible, i.e., FMS_Query leads to false positives over FMS_Block, but we did not observe this given our building block sets (see Methods for a more detailed discussion)."

2. What is “FMS_Block” and “FMS_Query”?

As the reviewer writes, FMS is implemented as follows:
“FMS = MCSS / n_heavy_block for MCSS / n_heavy_block < 1 or FMS = MCSS / n_heavy_query otherwise, where MCSS is number of heavy atoms in Max. Common SubStructure whereas n_heavy_block and n_heavy_query are the number of heavy atoms in building block and query molecule respectively.”

We will clarify why we distinguish between **FMS_Block** and **FMS_Query** and apologize again for our insufficient clarity in our original submission.

We start our response with copying the figure from our SI that the reviewer commented on. *We have updated this figure slightly to convey **FMS_Block** vs. **FMS_Query**:*

Fuzzy Matching Substructure (*Asymmetric Relationship*)

Intermediate Node
10 heavy atoms

Enforced Building Block (EBB)
8 heavy atoms

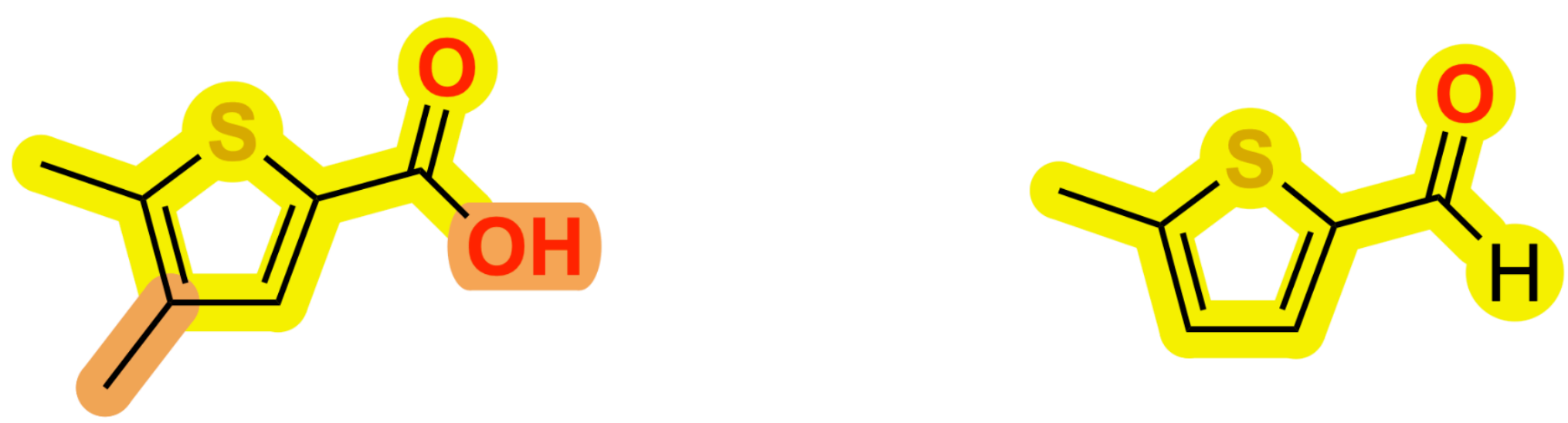

Number of Overlapping Heavy Atoms: 8

***FMS**$_{Block}$* **Normalized by EBB:** $\frac{8}{8} = 1.0$

***FMS**$_{Query}$* **Normalized by Node:** $\frac{8}{10} = 0.8$

We follow the reviewer’s notation:
n_heavy_block = # heavy atoms in the enforced building block
n_heavy_query = # heavy atoms in the query node (molecule node in a synthesis graph)

Building on the above notation, we define:

FMS_Block = MCSS / n_heavy_block
In the above picture, this is 8/8 = 1 (perfect match). However, the query (Intermediate Node) is not exactly the enforced block. This is the edge case we wanted to convey. The query is a larger structure that ***contains*** the enforced block as a substructure. ***The two bonds that differ are highlighted in red***.

FMS_Query = MCSS / n_heavy_query
In the above picture, this is 8/10 = 0.8

The implications of this asymmetric relationship is that if **FMS_Block** were used as a stand-alone reward function, it can result in false positives because **FMS_Block** = 1 does not necessarily mean equivalent structures. For this reason, we use the **FMS_Query** formulation when using matching substructure as a standalone reward function. We found that with the specific enforced block set we used, **FMS_Query** did not lead to false positives but one could imagine a scenario where this is also possible: For example if the “Intermediate Node” and “Enforced Building Block” in the figure above were swapped.

*We hope that re-defining “FMS” and “Asymmetric-FMS” as **FMS_Block** and **FMS_Query**, respectively, improves the manuscript’s clarity.*

Next, when using TANGO (TanSim with either **FMS_Block** or **FMS_Query**), even if **FMS_Block** gave a false positive, TANGO cannot equal 1. This is because the TanSim of non-equivalent molecules cannot be 1. We hypothesized that in the TANGO setting, this false positives behavior could actually be beneficial as a guiding signal. We empirically validate that this "potentially false positive" signal can be beneficial by testing "TANGO-**FMS_Block**" and "TANGO-**FMS_Query"** to denote using the **FMS_Block** and **FMS_Query** definition, in TANGO, respectively. We now show all these results in the main text and copy a part of the table here:

**Configuration** **N:** # seeds/5 generating $\geq$ 1 Solved (Enforced)	**Synthesizability**			**Solved (Enforced) Molecules** **Reaction Values:** Pooled (**M** Pooled Molecules) **Unique Enforced Block Values:** Per-seed		**Oracle Budget (Wall Time)**
	**Non-solved**	**Solved**	**Solved (Enforced)**	**# Reaction Steps**	**# Unique Enforced Blocks**	
TanSim (N=3)	2275 $\pm$ 143	7653 $\pm$ 142	1864 $\pm$ 1828	2.21 $\pm$ 1.16 (M=8731)	1.33 $\pm$ 0.47	10,000 (9h 11m $\pm$ 48m)
FMS_{Block} (N=4)	2166 $\pm$ 293	7792 $\pm$ 304	1 $\pm$ 0	2.14 $\pm$ 1.46 (M=7)	1.50 $\pm$ 0.50	10,000 (7h 35m $\pm$ 22m)
FMS_{Query} (N=3)	1967 $\pm$ 226	8002 $\pm$ 241	315 $\pm$ 563	2.67 $\pm$ 1.13 (M=1546)	1.33 $\pm$ 0.47	10,000 (8h 6m $\pm$ 23m)
TANGO-FMS_{Block} (N=5)	2229 $\pm$ 325	7711 $\pm$ 333	1744 $\pm$ 715	2.38 $\pm$ 1.25 (M=8315)	2.20 $\pm$ 0.75	10,000 (8h 12m $\pm$ 15m)
TANGO-FMS_{Query} (N=5)	2000 $\pm$ 211	7945 $\pm$ 217	1864 $\pm$ 1066	2.24 $\pm$ 1.18 (M=8472)	1.20 $\pm$ 0.40	10,000 (8h 14m $\pm$ 11m)

We highlight some key points but keep it brief as the full table is in the updated manuscript:

- **FMS_Block** has N=4/5 successful yet almost 0 molecules with an EBB (see **M**=7 which means there are 7 of these molecules across the 4 seeds). This is due to the false positive behavior
- **FMS_Query** rectifies this and results in more molecules with an EBB
- However, in the context of TANGO-**FMS_Block** vs TANGO-**FMS_Query**, the latter has higher variance and we observe the multi-parameter optimization to be worse. We copy a very small snippet of the table to support this statement:

TANGO-FMS_{Block} (N=5)	-8.29 $\pm$ 0.77<br>-8.12 $\pm$ 1.12 (M=8315)
TANGO-FMS_{Query} (N=5)	-7.80 $\pm$ 0.27<br>-7.84 $\pm$ 0.98 (M=8472)

* The table snippet reports the docking scores statistics
* Top values = per-seed average
* Bottom values = Pooled molecules across all seeds
* TANGO-FMS has better docking scores

Therefore, ***in the final implementation of TANGO***, we use **FMS_Block** *by default*. Only if a false positive is reached, then the code falls back to the **FMS_Query**. This implementation is what we refer to as TANGO and we added a sentence in the main text to convey this:

"Comparing the use of FMS_Block vs. FMS_Query in TANGO, we find that FMS_Block is more performant, with better docking scores, ligand efficiency, and diversity. Considering these empirical results, the final implementation of TANGO defaults to computing FMS_Block. Only in the event of a false positive signal is FMS_Query computed instead. From here on, we refer to TANGO-FMS_Block as TANGO."

Q4: The second part of TANGO is TS (this abbreviation is used in equation 1) or TanSym (used in Fig 4), I guess these two names refer to the same term which is not fully defined neither in maintext nor in SI. Based on the source code it seems to me the tanimoto similarity is between the Morgan fingerprint with radius=2.

A4: We apologize for the inconsistency in our naming. We now use "TanSim" to denote Tanimoto similarity both in Fig. 4 and in Equation 1. Additionally, we have added an explicit excerpt in the **Methods** section and copy this text below:

"To compute Tanimoto similarity, this work uses Morgan fingerprints with radius 2 and 1024 bits."

Q5: At this point, I would like to emphasize that it is extremely difficult to follow the authors' ideas when they do not define consistent terminology that is used throughout both the main text and the supplementary information (SI).

A5: We apologize again for the inconsistent terminology. We maintain consistency in the updated manuscript.

Q6: Morgan fingerprint and Tanimoto similarity are the most popular solution but there are many other options which may produce significantly different results. There are many other fingerprint types and method based of graph-convolution for molecular representation as well as similarity is not limited to Tanimoto (e.g. I have very promising experience with Tversky with carefully tuned parameters). I am really curious if changing these settings will improve the results for the TanSim reward function.

A6: Thank you for this suggestion. We ran additional experiments using Tversky with the following configurations:

- Tversky (a=0.5, b=0.5)
- Tversky (a=0.75, b=0.25)
- Tversky (a=0.25, b=0.75)
- TANGO-Tversky (0.50 * **FMS_Block** + 0.50 * Tversky a=0.5, b=0.5)

Note, we define FMS_Block in A3 to the reviewer's Q3:
FMS_Block = MCSS / n_heavy_block

This new results table is presented in the Supplementary Information Table S4. We copy a portion of the table here with brief points:

**Configuration**<br>**N:** # seeds/5 generating<br>$\geq$ 1 Solved (Enforced)	**Synthesizability**			**Solved (Enforced) Molecules**<br>**Reaction Values:** Pooled (**M** Pooled Molecules)<br>**Unique Enforced Block Values:** Per-seed		**Oracle Budget**<br>**(Wall Time)**
	**Non-solved**	**Solved**	**Solved (Enforced)**	**# Reaction Steps**	**# Unique Enforced Blocks**	
TanSim (N=3)	2275 ± 143	7653 ± 142	1864 ± 1828	2.21 ± 1.16 (M=8731)	1.33 ± 0.47	10,000<br>(9h 11m ± 48m)
Tversky<br>a=0.50, b=0.50 (N=5)	2324 ± 289	7615 ± 293	1595 ± 1088	2.33 ± 1.22 (M=7533)	1.20 ± 0.40	10,000<br>(9h 46m ± 26m)
Tversky<br>a=0.75, b=0.25 (N=4)	2295 ± 209	7635 ± 217	724 ± 869	2.34 ± 1.34 (M=3615)	1.75 ± 0.83	10,000<br>(9h 47m ± 33m)
Tversky<br>a=0.25, b=0.75 (N=4)	2190 ± 190	7740 ± 201	1243 ± 1031	2.35 ± 1.29 (M=5863)	1.50 ± 0.50	10,000<br>(9h 27m ± 20m)
TANGO<br>FMS_{Block}=0.50, TanSim=0.50 (N=5)	2229 ± 325	7711 ± 333	1744 ± 715	2.38 ± 1.25 (M=8315)	2.20 ± 0.75	10,000<br>(8h 12m ± 15m)
TANGO-Tversky<br>FMS_{Block}=0.50, Tversky=0.50<br>(a=0.50, b=0.50) (N=5)	2094 ± 352	7852 ± 358	976 ± 778	2.24 ± 1.24 (M=4793)	2.20 ± 0.98	10,000<br>(10h 3m ± 50m)

- Tversky (a=0.50, b=0.50) is comparable to TanSim and sometimes better
- However, in the TANGO case, TanSim is better

Q7: The definition of “Intermediate constraint” is unclear. Figure 1 suggests that it refers to starting materials used in the second or later steps of synthesis, whereas the definition in “Case 2” implies it can be a starting material used at any stage of synthesis. The term ‘intermediate’ in organic synthesis typically refers to compounds that are not starting materials but are produced in one step and utilized in another. Therefore, regardless of which definition is correct, the term is misleading.

A7: In our definition of “Intermediate Constraint”, we consider the case where one of the enforced building blocks is used at any step of the synthesis, except the very first step, which is reserved for “Starting Material Constraint”.

We apologize for our unclear writing, which as the reviewer correctly points out, implied that the enforced block can also be used as the starting material (very first step of the synthesis). We have improved the *preciseness* of our statement by explicitly writing that in the “Intermediate Constraint” case, the enforced block’s depth *is not* the max depth of the synthesis graph (first step of synthesis).

Q8: The definition of “Divergent synthesis” is not clear to me as well. Fig1 presents one synthetic graph to 4 products which has one common compound and each product requires at least one commercial building block for preparation. In ‘Case 3’ is a defined graph which has an intermediate for which all building blocks (leafs in sub-graph) are non-commercial. From ‘Case 3’ it is not clear for me how the constraint produces a graph with several products, as presented in Fig. 1.

A8: We define “Divergent Synthesis” as the case where a non-commercial molecule (made itself by commercially available precursors) is a common node used to synthesize numerous products. In Fig. 1, we previously denoted this by the presence of the orange node that represents a non-purchasable molecule. We agree with the reviewer that this lacked clarity and we have now modified the figure to show that the non-purchasable molecule itself is synthesized by commercially available building blocks. We copy this figure (*the only change is the Case 3: Divergent Synthesis graph visualization*)

[redacted]

In the **Methods** section describing “Case 3”, we write the following constraint:

$$\exists m \in G(M, R) \text{ s.t. } \forall EBB \in B_{enf},\ EBB \textit{ is non-commercial},\ \text{and } m = EBB \in B_{enf}$$

Stating that there exists a node ***m*** in the synthesis graph such that all enforced building blocks (EBBs) are non-commercial and that ***m*** is one of these non-commercial EBBs. As the constraint only states that *there exists a node **m***, the other nodes can be purchasable molecules used to arrive at the final generated product (shown as the blue circles in Fig. 1).

Our intended use case for this “Divergent Synthesis” definition is for users to be able to enforce scaffolds/proprietary reagents that are not necessarily in a commercial building block stock (e.g., ZINC). To mimic this for method development, we took some synthesis routes and extracted intermediate, non-commercial nodes to enforce. Cross-referencing a portion of Fig. 2:

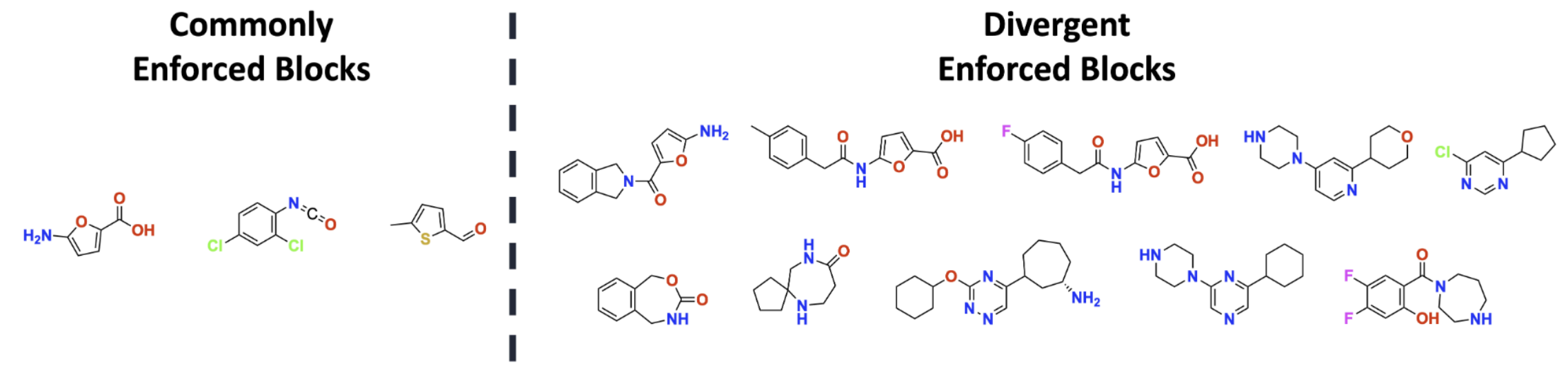

“Commonly Enforced Blocks” here are in the commercial building blocks stock. The “Divergent” blocks here are much larger and can represent core scaffolds of molecules. In practice, non-commercial nodes do not need to be derived from the commercial stock, and could be proprietary scaffolds/blocks to a lab.

Q9: The authors focus on enforcing the presence of selected building blocks (Benf) in synthetic graphs and claim, based on references [20-22], that in real-world applications, Benf is small. In the cited papers, the set of allowed building blocks was indeed limited, but this corresponds to B (the set of all allowed initial building blocks) rather than Benf. From my understanding of the real-world application of Computer-Aided Synthesis Planning (CASP), the enforcement of selected compound(s) in a synthetic pathway is target-dependent, and this information is provided to guide the algorithm when general searches (without restrictions) yield unsatisfactory results. Selecting Benf as a random set of simple molecules seems illogical. Instead, I suggest defining Benf as a set of compounds that possess important structural motifs relevant to the target or promote specific reactions along the pathway. For instance, Benf could consist of natural amino acids as a cheap source of chiral centers, allowing for the analysis of chirality sources in the resulting products.

A9: Thank you to the reviewer for the critical feedback. We begin our response by first clarifying our rationale for citations [20-22]. Previously, we cited these studies to support our statement that syntheses in general may wish to enforce specific building blocks (for example, to valorize waste as in citation 22). We did not include mention of **B_enf** (the set of enforced building blocks) and **B** (all allowed building blocks) here as we only wanted to motivate the idea of “enforced building blocks”. We agree with the reviewer that our writing was vague and imprecise. Accordingly, we have rewritten this section to the following:

“More generally, existing works have introduced the notion of starting-material constrained synthesis, where a small set of fixed reagents is used in the first step of reaction pathways to probe reactivity. These reagents are specifically selected due to their reactivity, as primordial substrates, or as toxic wastes to be degraded. Drawing the connection to synthesis planning, existing works have explored reacting a small set of fixed reagents with a larger set of general reagents to achieve valorization. For example, Wołos et al. demonstrated that industrial waste reacted with "basic" reagents can be valorized into known drug molecules. Similarly, the concept of "bioprivileged molecules" was introduced where molecules specifically derived from biomass could be used to synthesize novel and useful chemicals. Therefore, exploring the ability to valorize a small set of specific reagents is a pertinent problem. More recently, constrained retrosynthesis algorithms have been proposed that guide the identification of synthesis pathways towards specific reagents. However, to date, there are no molecular generative models that can enforce specific building blocks in the proposed routes.”

In the new passage, we first motivate the idea of starting-material constrained and then draw the connection to synthesis planning and explicitly state that there are use cases enforcing a small set of building blocks (**B_enf**) together with general building blocks (**B**). We also better distinguish citations 20-22 and thank the reviewer for their critical assessment. The reviewer is correct that citations 20:
https://www.nature.com/articles/s41586-018-0307-8
and 21:
https://www.science.org/doi/10.1126/science.aaw1955
use a small set of building blocks, and that these building blocks are actually the full set of allowed building blocks, i.e., **B_enf** = **B**. In the updated passage, we have separated these citations from 22:
https://www.nature.com/articles/s41586-022-04503-9
which does have a separate **B_enf** and **B**. In addition to the enforced industrial byproducts, the authors use 1,000 common reagents which are listed in their code repository:
https://github.com/rmrmg/wasteRepo/blob/main/popular_reagents.smi

The reviewer highlights that CASP applications usually specify selected blocks that are *target-dependent* and that selecting 100 random blocks like in our work, may be misaligned. We first agree with the reviewer and affirm that *constrained* retrosynthesis CASP applications commonly select specific, target-dependent building blocks, like in the Double-Ended Synthesis Planning (DESP) work:
https://arxiv.org/abs/2407.06334

However, we believe the application of our work has a genuine use case, even if it deviates (intentionally) from traditional constrained ***retrosynthesis*** CASP applications. Our intention with TANGO is to demonstrate another abstraction of the

constrained synthesizability problem: ***using enforced building blocks in de novo molecule design***. In the updated passage above, we provided another motivation for this problem by introducing "bio-privileged molecules", which encompass molecules derived from biomass. Since biomass can be abundant and is a renewable resource, there is interest in valorizing them into useful molecules. Subsequently, we do agree with the reviewer that randomly sampling 100 molecules as **B_enf**, may not ***always*** be applicable. However, we first discuss why we *do* believe this can be applicable before highlighting the new experiment we ran, that is aligned with the reviewer's suggestion. Firstly, these 100 blocks were used for method development, to demonstrate that the TANGO reward function can actually guide the generative model to satisfy constrained synthesizability. Next, we envision that labs possessing specific reagents in abundance and/or reagents that are inexpensive may be interested in repurposing them into novel molecules. In this scenario, the building blocks need not necessarily have any more specific attributes.

However, we agree with the reviewer that defining **B_enf** with specific structural motifs represents another use case. Accordingly, we have run a new real-world-inspired experiment and kindly refer the reviewer to our General Response here.

Q10: The FMS part of TANGO requires calculation of MCSS between all generated molecules and all enforced building blocks (EBB) which suggest it will be really time consuming for bigger EBB sets or compounds with many atoms. Could you please comment how this method scales with respect to molecule size and number of EBB?

A10: We run a scaling experiment to demonstrate the compute time for Tanimoto similarity and MCSS across varying enforced building blocks (EBB) set sizes. The experimental set-up was as follows:

1. Randomly sample {1, 10, 100, 1000, 10000, 100000} EBBs with molecular weight > 200 Da from the commercial stock we use (ZINC-based)
2. Randomly take a moderate length synthesis route (3 steps with 5 molecule nodes)
3. Compute Tanimoto similarity and MCSS comparing all 5 molecule nodes to all blocks in the EBB sets

We copy the new figure below and summarize key points:

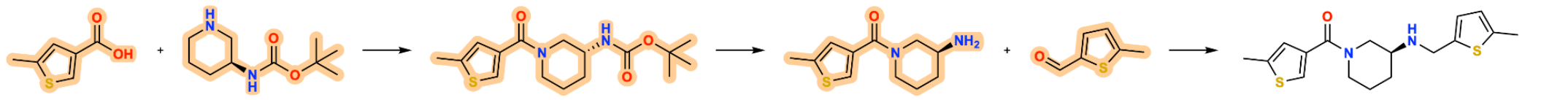

Time to compute Tanimoto similarity and FMS for the 5 highlighted molecules against *N* enforced building blocks

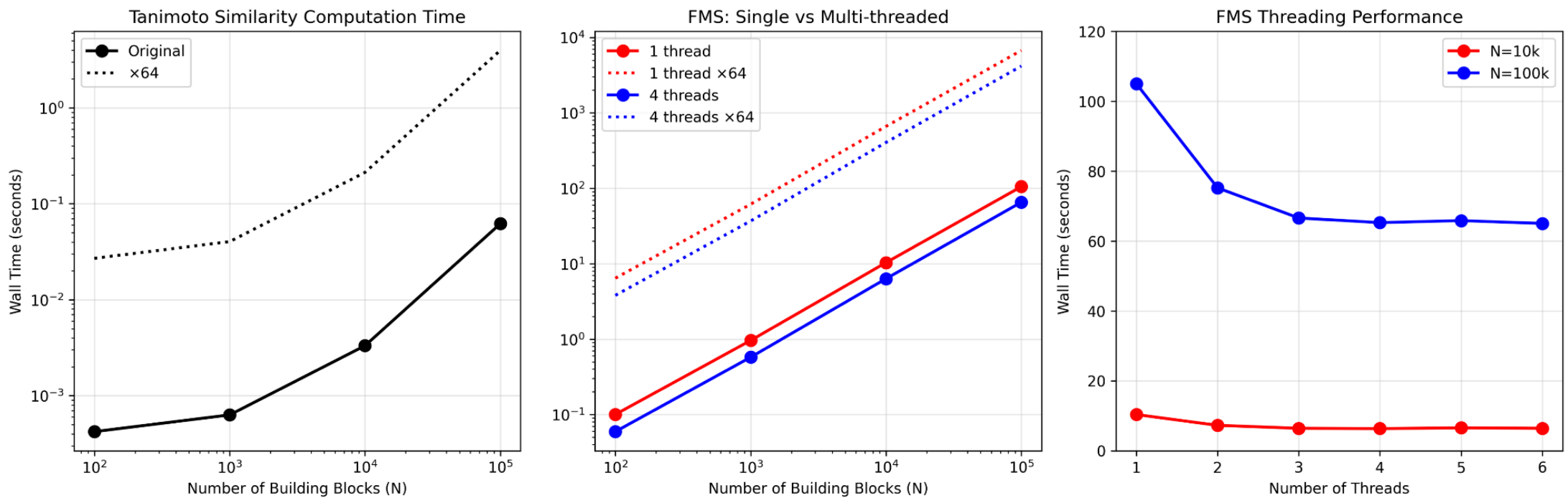

Left plot: TanSim wall time
Middle plot: FMS wall time
Right plot: Impact of multi-threading on wall time for the 10k and 100k EBB sets (4 was optimal in our tests)

- Tanimoto similarity is inexpensive
- MCSS does indeed take time for larger EBB sets, as the reviewer comments
- For example, with 10,000 EBBs, comparing the above route to all of these blocks takes about 10 seconds and less with 4 threads. **"x64"** denotes multiplying the time by 64 to mimic a max batch of 64 generated molecules per epoch (*assuming they all have a synthesis route requiring MCSS computation*). This can equate to 15-16 minutes

We have added a new section in the Supplementary Information (including the figure) commenting on this.

We do not envision most use cases to have > 1000 EBBs as we often want to enforce a "small" set because the building blocks serve a specific purpose (e.g., availability or curated set as proprietary blocks and/or enforcing a scaffold). Therefore, we believe the current implementation is fast enough for most practical applications. In the event that MCSS computation time ***does*** become the bottleneck, multi-processing across CPUs can be a straightforward solution.

Q11: Configurations from SI Table 3 (especially FG and brutal-force) should be defined.

A11: We apologize for missing this information. In the updated Supplementary Information, we have added a new excerpt describing these configurations and copy this text below:

*"**Brute-force** entails only optimizing for retrosynthesis model solvability, i.e., whether the retrosynthesis model returns a synthesis route (solved) or not. In this case, no notion of similarity between blocks in the synthesis route and the enforced building blocks is used as a learning signal. Therefore, if any enforced building blocks are indeed used, it would be by chance. The functional groups overlap (**FG**) reward function extracts the functional groups present in the synthesis route nodes and measures the overlap with those in the enforced building blocks. Concretely, for a given synthesis route node, compute: # shared functional groups between the node and an enforced building block, divided by the total number of functional groups in the enforced building block. Do this for every enforced building block and then return the mean. The functional groups themselves are defined by SMARTS patterns encoding common groups adapted from:*
https://www.daylight.com/dayhtml_tutorials/languages/smarts/smarts_examples.html
"

Q12: In most cases obtained paths are short (1-4 steps) and based on Commonly Enforced Blocks. It is worth discussing the diversity of obtained products in the sense of chemical structure (e.g. using fingerprint similarity and Murcko scaffolds), physical property (mass, logP, total PSA and so on) and synthetic paths (what type of reaction is used).

A12: We have augmented every results table with diversity metrics (**IntDiv1**, **#Circles**, and **# Bemis-Murcko scaffolds**). To also add some information on the physical properties while keeping the size of the tables manageable, we additionally augment the tables with **Ligand Efficiency** which we compute by taking the absolute value of docking scores divided by the number of heavy atoms. A molecule with less heavy atoms achieving a better docking score will receive a higher **Ligand Efficiency**.

We add analysis of the synthetic paths by pooling (across seeds) all generated molecules and showing the common reactions. We analyze the experiments presented in the main text and also the "100 Blocks Purged" experiment as the reviewer asks about this in their next question. All reactions were labeled using NameRXN. We copy the new figure below (it has been added to the Supplementary Information):

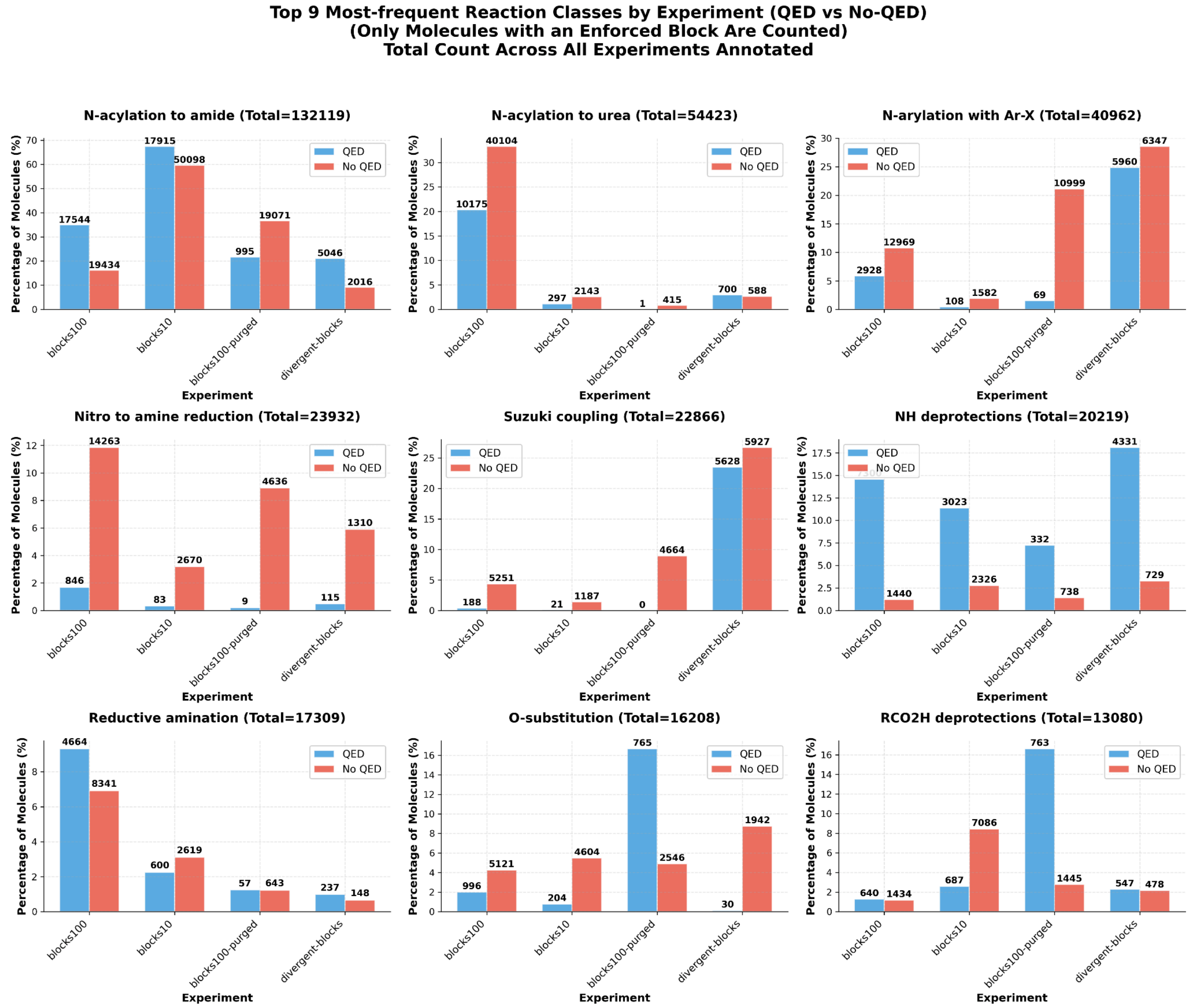

The plot extracts the top-9 most frequent reaction classes amongst all experiments. For each experiment, the number of occurrences of that reaction class across the 10 seeds is annotated. The percentage of that reaction relative to all the reactions used is the bar plot height. Experimental configurations with and without the QED constraint are reported side-by-side.

- Amide forming reactions were by far the most common
- Deprotection reactions are common
- "Nitro to amine reductions" were used almost exclusively in the non-QED experiments

TANGO does not explicitly consider reaction classes and is only focused on guiding the generative model towards enforced building blocks (EBBs). Depending on the specific building EBB, it will inherently be more compatible with specific reactions, e.g., a EBB containing an acyl chloride is particularly amenable to an amide bond forming reaction. Therefore, we only empirically comment on the reaction frequencies.

This information has been added to a new section in the Supplementary Information titled "Common Reaction Types".

Q13: Results from “100 Blocks Purged“ experiments are very interesting, obtained paths are longer (3-7 steps) which suggests obtained compounds can be more diverse. I think authors should also analyze obtained compounds and paths. Is there a new “lucky” EBB?

A13: Thank you to the reviewer for this interesting question. The compounds from the **“100 Blocks Purged”** are not necessarily more diverse and we support this statement by comparing the diversity metrics between enforcing “**100 Blocks**” (with QED) and **“100 Blocks Purged**” (with QED).

We pool all unique generated molecules enforcing at least one block and report the diversity metrics below (this information is reported in the Supplementary Information):

“100 Blocks”
(19,221 pooled molecules, 10/10 seeds successfully generated at least one molecule with an enforced block)
IntDiv1: 0.762 (higher is more diverse)
#Circles: 7 (higher is more diverse)
# Bemis-Murcko Scaffold: 12672

“100 Blocks Purged”
(8,562 pooled molecules, 4/10 seeds successfully generated at least one molecule with an enforced block)
IntDiv1: 0.533
#Circles: 3
# Bemis-Murcko Scaffold: 682

This can be partly explained by the **“100 Blocks Purged”** now converging to a new common block. We had previously further interrogated whether this was another “lucky” block by also running the experiment **with QED omitted**. Our hypothesis was that the multi-parameter optimization function also notably affects the block that is enforced. In the original manuscript submission, we did not explicitly comment on this and we add this information in this updated revision. We have added an additional section in the Supplementary Information titled “A New Lucky Block?” with two accompanying figures. We first copy this new section below:

“In Table <Purged Block Experiments>, the results show that the generated molecules converge to a small number of unique enforced building blocks. This suggests the possibility of another "lucky block". We show that while "100 Blocks Purged" (with QED constraint) does converge mostly to another specific building block, removing the QED constraint results in different and more blocks (with some overlap compared to the QED constraint experiment). This supports the hypothesis that certain blocks can be more favorable for the objective function. To show that

there are many unique blocks enforced, we visualize the synthesis route of the best docking score molecule for each unique enforced block across the "100 Blocks Purged (with QED)" and "100 Blocks Purged (without QED)" experiments in Figures <New Figure 1> and <New Figure 2>, respectively. Importantly, we also annotate the frequency of the specific enforced building block compared to the full pooled set of molecules, i.e., out of all generated molecules across the successful seeds, how many times was a particular enforced block used? Note that if a particular generated molecule contains > 1 enforced building block, all are counted in the frequencies."

The "100 Blocks Purged (with QED)" experiment has 3 unique blocks but 1 is overly represented. Conversely "100 Blocks Purged (without QED) has 5 unique blocks with a more even distribution. We copy the new figures below (they are in the Supplementary Information):

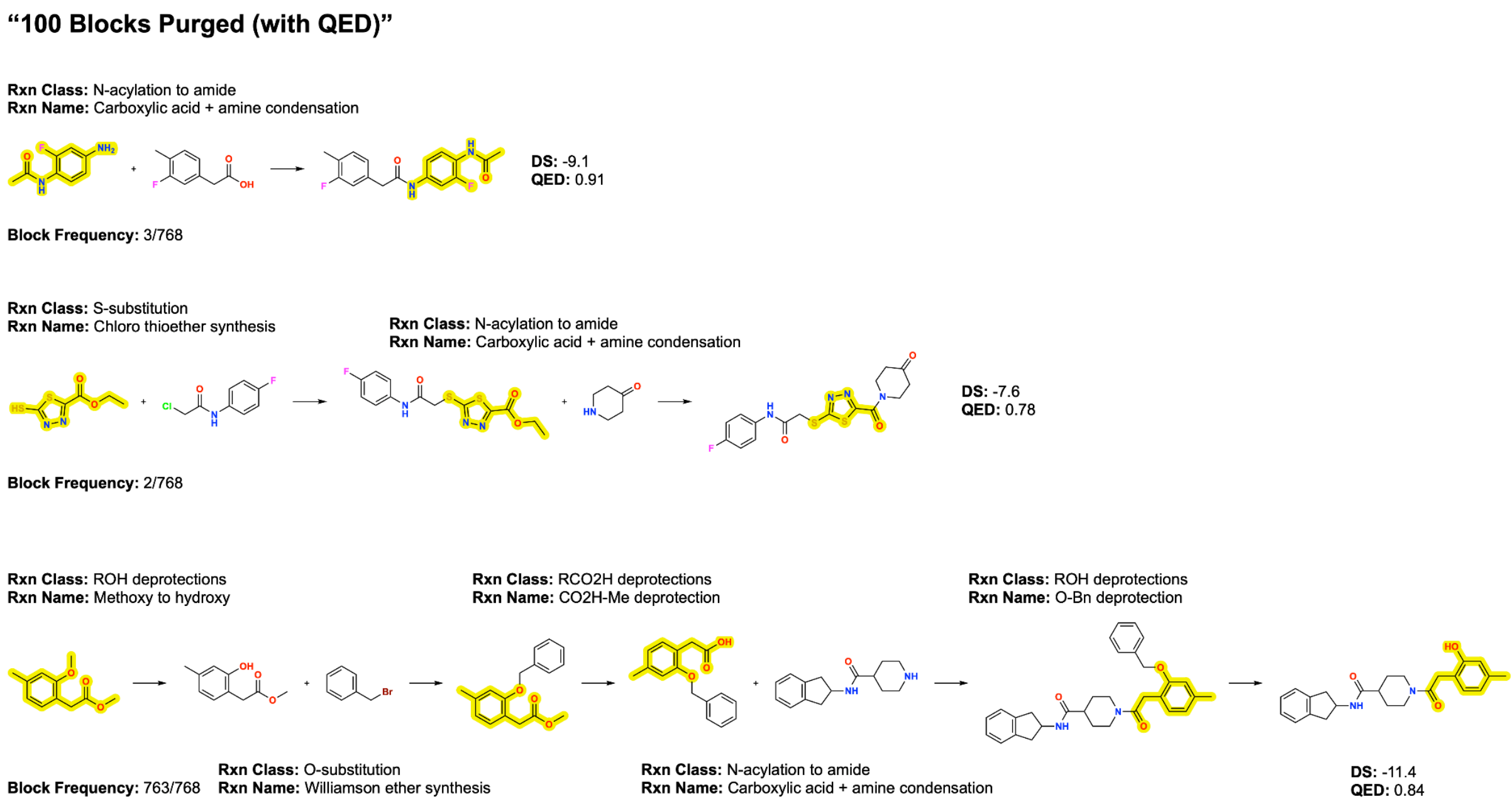

“100 Blocks Purged (without QED)”

Rxn Class: N-acylation to amide
Rxn Name: Amide Schotten-Baumann

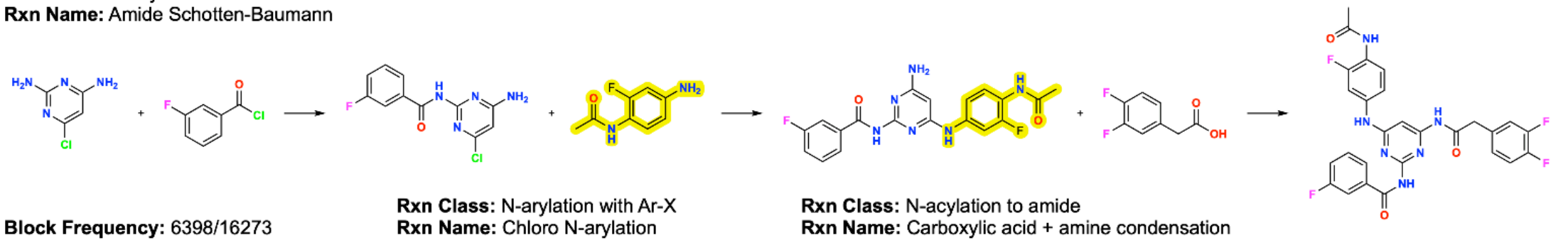

Rxn Class: N-arylation with Ar-X
Rxn Name: Chloro N-arylation

Rxn Class: N-acylation to amide
Rxn Name: Carboxylic acid + amine condensation

DS: -12.6
QED: 0.22

Block Frequency: 6398/16273

Rxn Class: O-substitution
Rxn Name: Williamson ether synthesis

Rxn Class: Nitro to amine reduction
Rxn Name: Nitro to amino

Rxn Class: N-acylation to amide
Rxn Name: Carboxylic acid + amine condensation

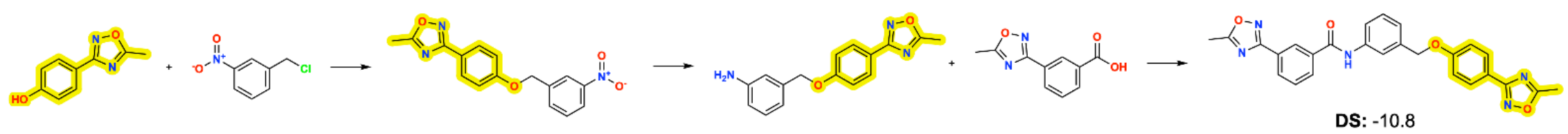

DS: -10.8
QED: 0.35

Block Frequency: 171/16273

Rxn Class: Suzuki coupling
Rxn Name: Bromo Suzuki-type coupling

Rxn Class: Suzuki coupling
Rxn Name: Bromo Suzuki-type coupling

Rxn Class: N-acylation to amide
Rxn Name: Carboxylic acid + amine condensation

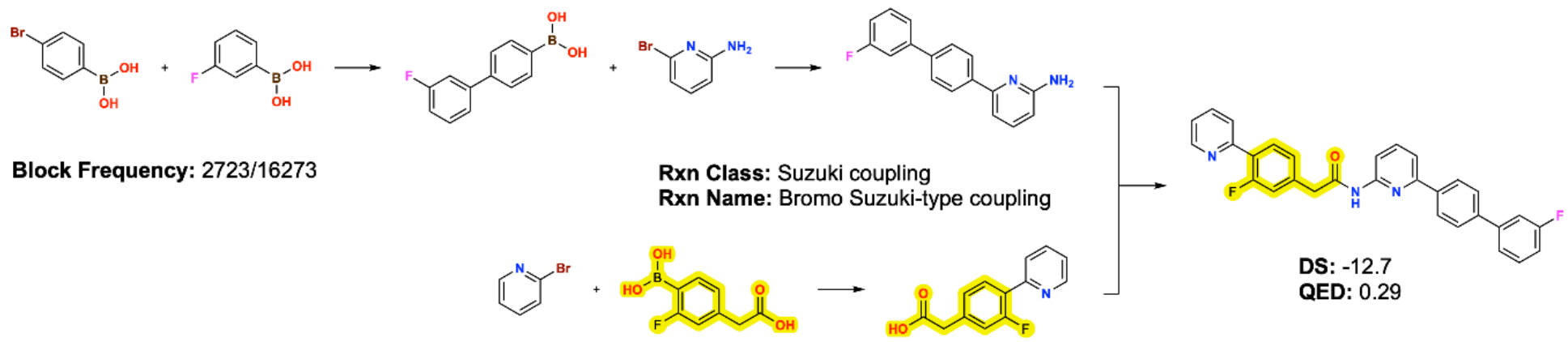

Block Frequency: 2723/16273

Rxn Class: Suzuki coupling
Rxn Name: Bromo Suzuki-type coupling

DS: -12.7
QED: 0.29

Rxn Class: N-acylation to amide
Rxn Name: Carboxylic ester + amine reaction

Rxn Class: S-substitution
Rxn Name: Chloro thioether synthesis

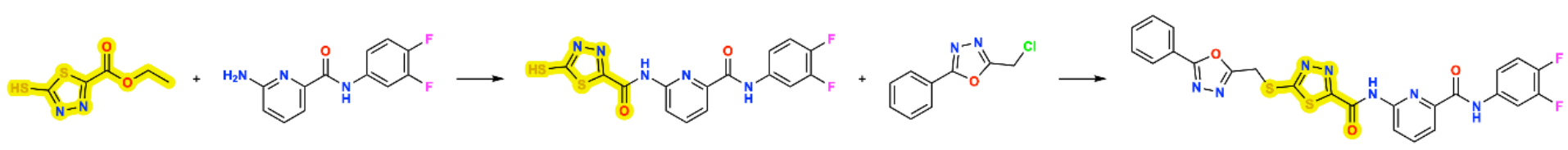

Block Frequency: 2266/16273

DS: -10.7
QED: 0.25

Rxn Class: N-acylation to amide
Rxn Name: Carboxylic acid + amine condensation

Rxn Class: O-acylation to ester
Rxn Name: Ester Schotten-Baumann

Rxn Class: N-acylation to amide
Rxn Name: Carboxylic acid + amine condensation

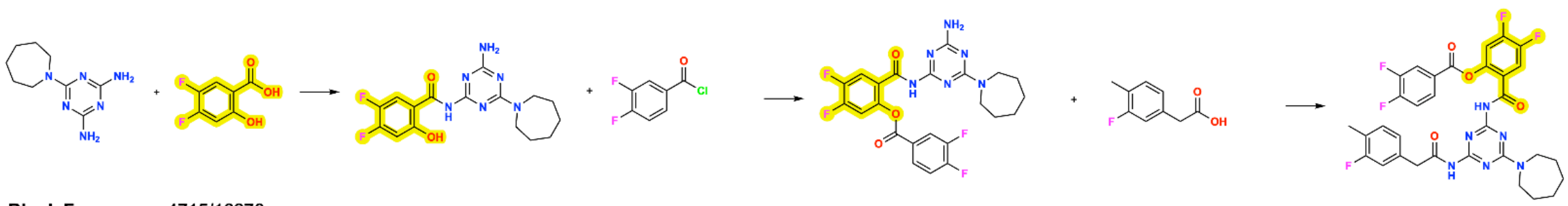

Block Frequency: 4715/16273

DS: -12.8
QED: 0.14

Reviewer #3:

Q1: In Figure 1, the roles of M and N (especially how it relates to the reward model's performance) are not entirely clear to me. Could the authors reorganize the content to present it in a clearer and more comprehensible manner?

A1: We apologize for the insufficient clarity. We have changed all the tables and included new molecule metrics based on feedback from reviewer 2. To address the comment here, the tables now also explicitly state that:

- **N:** # seeds generating at least one molecule with an enforced block, i.e. it was a successful run
- **M:** # pooled molecules that have an enforced block across all seeds.

We kindly refer the reviewer to our General Response.

Q2: Why is an oracle budget of 10,000 chosen for running the retrosynthesis search library? Does varying the oracle budget significantly impact the reward function? I believe that with different oracle budgets, the synthesis graph of the generated molecule would differ, which would, in turn, affect the TANGO reward. Is this correct?

A2: Yes, the reviewer is correct that varying the oracle budget would impact the reward function, as a higher budget would, in general, allow one to optimize the reward function to a greater degree. We set the computational budget to 10,000 retrosynthesis search calls for three reasons:

1. A 10,000 oracle calls budget is common in benchmarks on efficiency of optimization, such as the Practical Molecular Optimization (PMO) benchmark: https://openreview.net/forum?id=yCZRdI0Y7G

2. With a 10,000 oracle budget, it enables us to run more experiments under limited compute as each experiment takes about 8 hours currently. This enabled us to run over 300 experiments (including replicates) in this paper while under the constraints of a GPU queuing cluster.

3. For most experimental set-ups in this paper, many generated molecules already receive perfect TANGO reward (reward=1 is perfect and indicates that there is an enforced block in the synthesis graph) under 10,000 oracle calls. This is affirmed by the results showing that generated molecules satisfy constrained synthesizability, i.e., their synthesis routes contain an enforced building block.

Early in our investigation, we tried lower oracle budgets (3000 and 5000). These results are in Supplementary Information Sections 5.1 and 5.2. We found that a 10,000 oracle budget enables most reward function formulations to successfully guide the generation of molecules satisfying constrained synthesizability.

Q3: Probably wrong usage of the terms 'Sparse' and 'Dense' in the context of reinforcement learning. In this domain, a Dense reward typically refers to scenarios where the reward function provides detailed feedback at every step or action in an episode. However, in this case, the authors might mean that TANGO is more continuous rather than binary.

A3: We thank the reviewer for highlighting this and we have replaced all mentions of 'Sparse' and 'Dense' with 'Binary' and 'Continuous', respectively. TANGO still operates in a sparse reward setting as only the final molecule has an associated reward, i.e., all intermediates rewards are 0.

We would like to thank the reviewers for reading our updated manuscript and for their constructive criticism in the previous revision round. We respond to reviewer 2's comments below.

Reviewer #2:

Q1: SI figure marked too many atoms.

A1: We apologize for this oversight from our original response. We have corrected this mistake and copy the figure below:

Fuzzy Matching Substructure (*Asymmetric Relationship*)

Intermediate Node
10 heavy atoms

Enforced Building Block (EBB)
8 heavy atoms

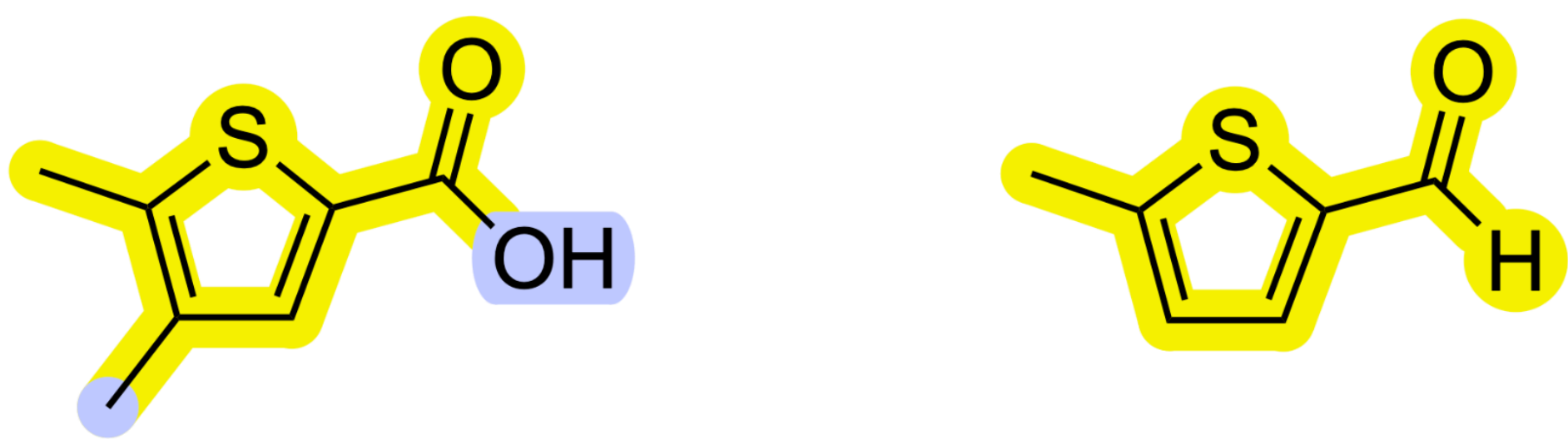

Number of Overlapping Heavy Atoms: 8

***FMS*$_{Block}$ Normalized by EBB:** $\frac{8}{8} = 1.0$

***FMS*$_{Query}$ Normalized by Node:** $\frac{8}{10} = 0.8$

The left molecule now correctly shows that 2/10 atoms are not matched.

Q2: Incorrect IntDiv1 citation.

A2: Thank you for this comment, we have modified the citation to the paper linked by the reviewer: arxiv.org/pdf/1708.08227.

Q3: Include definitions of IntDiv1 and #Circles.

A3: We have included definitions of IntDiv1 and #Circles in the Methods section "Metrics" sub-section and copy the new text below:

"Both IntDiv1 and #Circles use Morgan fingerprints with radius=2 and nBits=1024. IntDiv1 is defined as 1-(average pair-wise Tanimoto similarity of the generated set). #Circles is defined as the largest sub-set of generated molecules, where all molecules are dissimilar to each by a distance metric and threshold (0.75 in this work). Accordingly, all molecules in the sub-set are < 0.25 Tanimoto similarity."

Q4: Citation consistency and ordering.

A4: We now cite IntDiv1 on its first appearance and make sure citations appear chronologically in the order they appear in the main text. We have also ensured the citations are consistent: For papers with one or two authors, we include all authors' names. In cases of more authors, we use *et al.* after all co-first authors' names. We use *et al.* to manage the length of the references.
